# Population Genomics Analysis Revealed Origin and High-altitude Adaptation of Tibetan Pigs

**DOI:** 10.1038/s41598-019-47711-6

**Published:** 2019-08-07

**Authors:** Yun-Fei Ma, Xu-Man Han, Cui-Ping Huang, Li Zhong, Adeniyi C. Adeola, David M. Irwin, Hai-Bing Xie, Ya-Ping Zhang

**Affiliations:** 1 State Key Laboratory of Genetic Resources and Evolution, and Yunnan Laboratory of Molecular Biology of Domestic Animals, Kunming Institute of Zoology, Chinese Academy of Sciences, Kunming, 650223 China; 2 Kunming College of Life Science, University of Chinese Academy of Sciences, Kunming, 650204 China; 3 University of Chinese Academy of Sciences, Beijing, 100049 China; 4 Laboratory for Conservation and Utilization of Bio-resource, and Key Laboratory for Animal Genetic Diversity and Evolution of High Education in Yunnan Province, Yunnan University, Kunming, 650091 China; 5 Department of Laboratory Medicine and Pathobiology, University of Toronto, Ontario, M5S 1A8 Canada

**Keywords:** Genomics, Evolutionary genetics, Phylogenetics, Population genetics

## Abstract

Tibetan pig is native to the Qinghai-Tibet Plateau and has adapted to the high-altitude environmental condition such as hypoxia. However, its origin and genetic mechanisms underlying high-altitude adaptation still remain controversial and enigmatic. Herein, we analyze 229 genomes of wild and domestic pigs from Eurasia, including 63 Tibetan pigs, and detect 49.6 million high-quality variants. Phylogenomic and structure analyses show that Tibetan pigs have a close relationship with low-land domestic pigs in China, implying a common domestication origin. Positively selected genes in Tibetan pigs involved in high-altitude physiology, such as hypoxia, cardiovascular systems, UV damage, DNA repair. Three of loci with strong signals of selection are associated with *EPAS1*, *CYP4F2*, and *THSD7A* genes, related to hypoxia and circulation. We validated four non-coding mutations nearby *EPAS1* and *CYP4F2* showing reduced transcriptional activity in Tibetan pigs. A high-frequency missense mutation is found in *THSD7A* (Lys561Arg) in Tibetan pigs. The selective sweeps in Tibetan pigs was found in association with selection against non-coding variants, indicating an important role of regulatory mutations in Tibetan pig evolution. This study is important in understanding the evolution of Tibetan pigs and advancing our knowledge on animal adaptation to high-altitude environments.

## Introduction

The Qinghai-Tibet Plateau is a hotspot for high-altitude adaptation studies in diverse native organisms, including humans^1–4^, domestic animals^5–7^, and wild life^8,9^. Tibetan pig is the indigenous pig (*Sus scrofa domesticus*) breed native to the Qinghai-Tibet Plateau, providing Tibetans with stable source of meat. Earliest records from the book of Tang show that Tibetans raised domestic pig in the 7th century^10^. Tibetan pig adapts well to harsh plateau environments and extensive feeding condition, mainly in search of food by themselves. Physiological studies show that the Tibetan pig have evolved physiological adaptations to the high-altitude hypoxia, such as a thicker alveolar septum with more developed capillaries^11^, larger and strong heart^12^.

The origin of Tibetan pigs is still under debate. Earlier studies have proposed different origin models. The earliest study based on phylogenomic analysis of mitochondrial DNA (mtDNA) sequence variations in 567 domestic pigs (including 29 Tibetan pigs) and 155 wild boars across Asia conducted by Wu *et al*. (2007) showed that majority of Tibetan pigs shared haplogroups with domestic pigs from Yangtze River and northern China^13^. Later, Yang *et al*. (2011) suggested a local origin of Tibetan pigs from Tibetan highlands by analyzing mtDNA variants in more pig samples from Asia^14^. In a recent nuclear genome research, Li *et al*.(2013) considered them as wild boars that have evolved without artificial selection^12^. However, Ai *et al*. (2014) defined the Tibetan pig as a domestic breed and found the essential role of admixture with neighboring Chinese domestic pigs during their breeding^15^.

Clarifying the relationship between Tibetan and other Chinese wild and domestic pigs will provide important information to guide the choice of research approach used to reveal genetic mechanism underlying high-altitude adaptation in Tibetan pigs. Whole genome nuclear variants would provide more comprehensive information for studying the origin of Tibetan pigs than nuclear chip study and a complementary perspective to mtDNA evidence. In this study, we conducted whole genome analysis of 229 pigs, including Tibetan as well as other pig populations across Eurasia. First, we focused on the origin of Tibetan pigs by conducting phylogenomic and population structure analysis. Then, we compared the genomes of Tibetan pigs with those of low-land pigs that showed the closest relationship with Tibetan pigs in the phylogenomic analysis. Finally, we screened the signatures in Tibetan pig genomes that experienced selection since their arrival in Tibet. This study will provide useful information in resolving the origin and mechanism underlying high-altitude adaptation in Tibetan pigs and give signals on the importance of clear origin history before conducting evolutionary adaptation analysis of special population or species in the future study.

## Results

### Whole genome resequencing and identification of sequence variants

In this study, we sampled 48 domestic pigs and wild boars (Tibetan pig: 11, lowland pigs: 28 samples from 11 breeds, wild boar: 9) across China for whole genome resequencing (Supplementary Table s1). A total of 601 Gb of raw paired-end reads were generated. In order to produce more comprehensive and more reliable results, 181 genomes of Eurasian wild boars and domestic pigs and four other outgroup species from SRA database (http://www.ncbi.nlm.nih.gov/Traces/sra/) were also incorporated in our analysis (Supplementary Table s1).

The combined dataset contained a total of 3.2Tb of sequences that were mapped to the pig reference genome (*Sus scrofa* 10.2) after trimming low quality regions with QcReads^16^. The average sequencing depths for the different breeds ranged from 2.35× to 22.29× (Supplementary Tables s1 and s2). Over 49.6 million SNPs were identified among the 229 Eurasian pig samples.

### Genetic structure analysis

**Figure 1:**
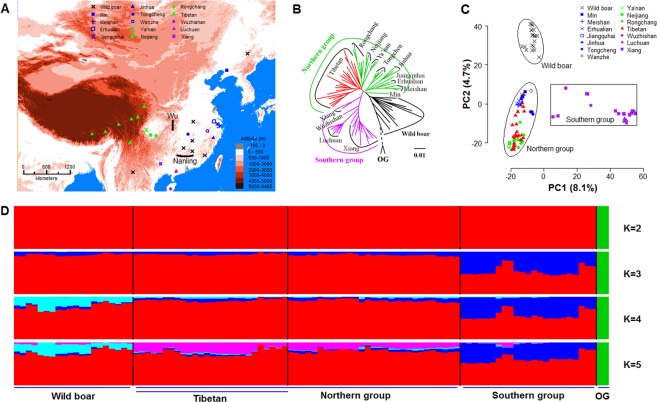
Geographic distribution and population genetic analysis of the Tibetan pigs and other Chinese pigs. (**A**) Sites origin for the different Chinese breeds and wild boars. Sampling sites of the wild boar only include newly sequenced samples. (**B**) Whole-genome Neighbor-Joining tree of pigs in this study. The branch length of OG artificially shortened and is shown as a dashed line. (**C**) Principal component analysis. PC1 is the first principal component. PC2 is the second principal component. (**D**) Population structure analysis with K from 2 to 5. Abbreviations (**B**,**D**) defined as follow. OG: outgroup (Sumatra wild boar).

First, we studied the genetic structure of Tibetan pigs and their relationship with other Chinese wild and domestic pigs. The relationship between the pig populations might have been affected by recent gene flows. It is common to observe the introduction of European commercial pig breeds into China in order to improve the performance of local pigs^17^. To exclude the effect of recently intercontinental gene flows, we examined the population structure^18^ of all the samples to estimate the influence of European commercial pigs on Chinese pigs. Unexpectedly, we observed that over 30% of the 183 Asian individuals had European genetic components, with a proportion ranging from 10% to 99% (Supplementary Fig. s1 and Table s3). To reduce the effects from undisclosed gene flows, only 98 Asian pig samples (26 Tibetan pigs, 20 Chinese wild boars, 52 Chinese domestic pigs from 13 breeds) (Fig. 1A) that showed European genetic component fraction less than 5% were included in the subsequent analysis (Supplementary Table s3).

Three genetic methods were employed to aid in interpreting the evolutionary history of Tibetan pigs. Wild boars from Sumatra in Indonesia, proposed site for wild boar origin^19^, were used as outgroup, as they had more genetic similarity with pigs as compared to *Sus cebifrons*, *Sus celebensis*, *Sus verrucosus* and *Sus barbatus* (Supplementary Fig. s1 and Table s3). The rooted Neighbor-Joining (NJ) phylogenetic tree of the pig genomes across China was constructed^20^ and the wild boars were located near the root of the tree (Fig. 1B). All domestic pigs diverged from the Chinese wild boars and outgroup (wild boars from Sumatra), and formed two different clades separated by Nanling Mountains (Fig. 1A), namely northern group and southern group (Fig. 1B). The principal component analysis (PCA) demonstrated that the domestic pigs from southern group separated from the wild boars and other domestic pigs from northern group in PC1 (8.1%), while Chinese wild boars and domestic pigs from northern group separated in PC2 (4.7%) (Fig. 1C). This result suggests that the genetic structure of the southern group differs greatly from the wild boar and domestic pigs from northern group in China. Tibetan pigs were interspersed among domestic pigs from northern group. We also employed STRUCTURE^18^ to analyze the population structure among the samples with different values for K (from 2 to 5). The southern group genetic component was the first to separate from wild boar and other domestic pigs from the northern group (K = 3, Fig. 1D). The Tibetan pigs shared similar genetic components with other Chinese domestic pigs from northern group and differed from the Chinese wild boar (CWB) (K = 4 to 5, Fig. 1D). The STRUCTURE results showed a similar genetic pattern with PCA analysis.

### Analysis of selective signatures in the Tibetan pig genomes

The above analysis indicated a close relationship between Tibetan pigs and low-land domestic pigs from northern group in China. To investigate on the genetic adaptation to high altitude in Tibetan pigs, we only compared the genomes of Tibetan pigs with those of low-land domestic pigs from the northern China. Furthermore, Chinese pigs with more than 5% European genetic component were also removed to avoid the influence from recent gene flows between Eurasia populations. Finally, our dataset included 26 Tibetan pigs (average altitude >3,000 m) and 29 low-land pigs from northern group (average altitude of no more than 800 m) (Supplementary Table s1) that were used as the control population.

**Figure 2:**
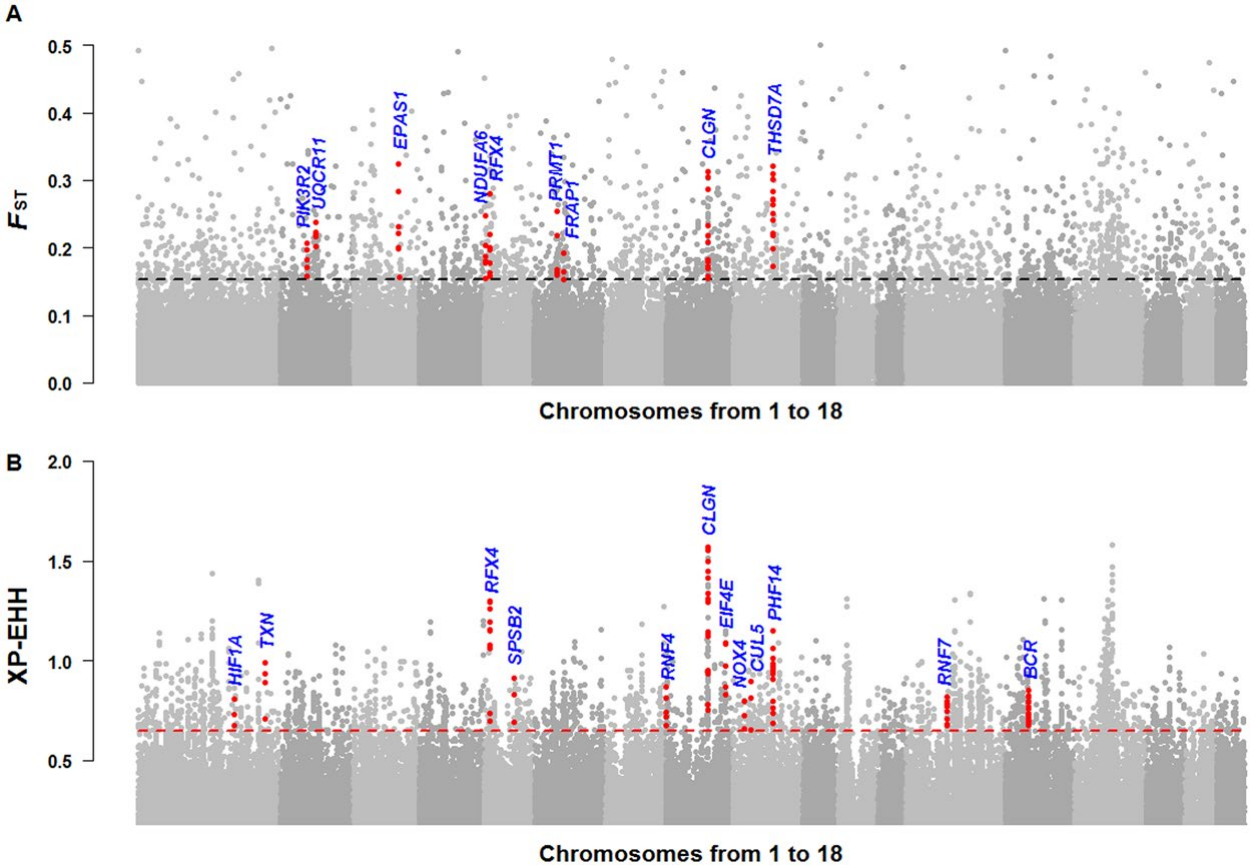
Selection signals on the autosomes of the Tibetan pigs. (**A**) Genome-wide *F*_ST_ values in the Tibetan pigs. The black dotted line represents top 99% threshold of *F*_ST_ at whole-genome level. (**B**) XP-EHH values of the autosomes in the Tibetan pigs. Each dot represents the average value of a10-kb sliding window. The red dotted line represents top 99% threshold of XP-EHH at whole-genome level.

Genomic regions that have experienced selection show specific signatures, such as diverging allele frequencies between populations^21^ and extended haplotype homozygosity^22^. We scanned for selective sweeps using two comparative genomic methods, *F*_ST_ (fixation index) and XP-EHH^23^, in 10 kb siding windows. A total of 33,432,165 autosomal SNPs were identified within the genomic sequences of Tibetan and control populations. In differentiation analysis, high *F*_ST_ values with elevated derived allele frequency was used to detect genomic regions in the Tibetan pigs highly differentiated from other low-land pigs. Candidate sweeps were identified as a clustering of at least three consecutive (except for undetermined genomic gaps) 10-kb sliding windows with genome-wide top 1% *F*_ST_ or XP-EHH values. A total of 4.68 Mb (the longest sweep: 270 kb, average length: 45 kb) and 14.07 Mb (the longest sweep: 220 kb, average length: 54 kb) of genomic sequence were defined as selective sweeps in genome of Tibetan pig by *F*_ST_ and XP-EHH analyses, respectively (Fig. 2, Supplementary Tables s4 and s5). Within the sequences identified by *F*_ST_ and XP-EHH, 70 and 211 potentially positively selected genes (PSGs) were identified, respectively, with eight genes identified by both approaches (Supplementary Table s6). Majority of our 273 candidate PSGs were found for the first time, only these four are in common (*SERGEF*, *RAPGEF2*, *LEF1*, *HIF1A*) with the 215 candidate PSGs reported by Li *et al*.^12^, only six genes (*THSD7A*, *SEC*. *63*, *OSBPL1A*, *MFSD2A*, *FAM149A*, *DPPA4*) were in common when compared with the 489 genes identified by Ai *et al*.’s^15^.

Literature mining of the 273 PSGs biological function indicated that many PSGs participated in physiological processes, such as response to hypoxia, cardiovascular system, lung and gas exchange, mitochondria or respiratory chain, DNA damage repair, spermatogenesis, embryo development, tumor/cancer, neural development, immunity and apoptosis (Supplementary Table s7).

**Figure 3:**
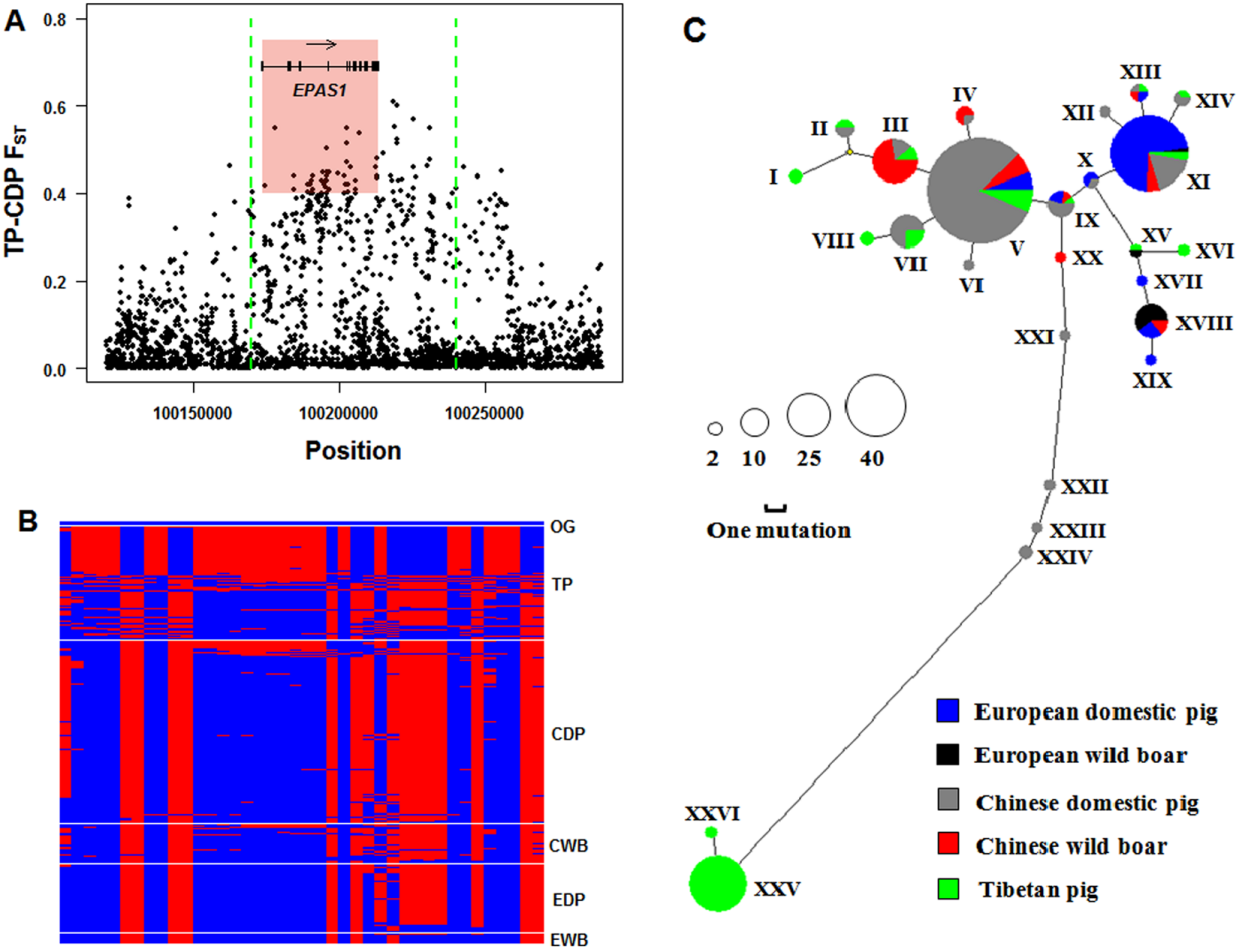
Selective signals and haplotypes of *EPAS1*. (**A**) *F*_ST_ values of each SNP between the Tibetan and control populations. The x axis is the physical position on chromosome 3 (*Sus scrofa* 10.2 build). The region between the two green dashed lines is the candidate sweep. The red box defines the SNPs with the largest genetic differentiation, used to analyze haplotypes. (**B**) Haplotype pattern of highly differentiated SNPs between the Tibetan and control pig populations using 227 pigs from East Asia and Europe. Each column is a polymorphic genomic location and each row is a phased haplotype. The blue cell represents the ancestor allele and the red cell represents the derived allele. OG: outgroup, TP: Tibetan pigs, CDP: Chinese domestic pig, CWB: Chinese wild boar, EDP: European domestic pig, EWB: European wild boar. (**C**) The Median-Joining network of haplotypes within *EPAS1*. Only haplotypes with frequency more than 1 were used to draw the network.

Six PSGs (*EPAS1*, *HIF1A*, *RNF4*, *TNFSF10*, *PDE1A*, *PDE3*) were related to “response to hypoxia”. *EPAS1*, encoding hypoxia-inducible factor 2-alpha subunit, is well known for its role in adaptation to hypoxia in humans and animals native to high-altitude levels^24–26^. *EPAS1* is the only gene located within a 70 kb sweep region on chromosome 3 in Tibetan pigs (chr3: 100, 170, 001–100, 240, 000) (Fig. 2A). To analyze the haplotypes, the 40 most differentiated SNPs (*F*_ST_ > = 0.4, red box in Fig. 3A) across the entire gene region of *EPAS1* (39.7 kb) were phased in all of the Eurasian samples. Intriguingly, we found that the Tibetan pigs contained a medium-frequency haplotype (haplotype XXV), that was extremely differentiated from the other Eurasian haplotypes (Fig. 3B,C), this differentiated pattern was similar to previous observation of *EPAS1* between Tibetans and Han Chinese^27^.

Genes involved in circulatory and respiratory systems were also detected under positive selection in Tibetan pigs. 34 candidate PSGs (Supplementary Table s7) were detected in association with the cardiovascular system and five genes (*CYSLTR2*, *PHF14*, *RNF150*, *TIMELESS*, *SCAP*) in association with lung and gas exchange. *BCR* (Breakpoint Cluster Region), which was found within a 180 kb sweep region (Chr14: 52, 720, 001–52, 900, 000) (Fig. 2B) formed the fusion protein BCR-ABL with *ABL* that affects hypoxia-induced pulmonary hypertension^28^ and the expression of vascular endothelial growth factor^29^. *THSD7A*, across two adjacent discontinuous sweeps (Fig. 2A and Supplementary Fig. s2A), is a conserved gene in vertebrates that is known to be involved in endothelial cell migration and embryonic angiogenesis^30,31^. A missense mutation (Lys561Arg) in *THSD7A* was observed with elevated derived allele frequency (DAF) in the Tibetan pigs (Tibetan pigs: DAF = 0.79, low-land pigs: DAF = 0.25), at a site that is highly conserved among vertebrates (Supplementary Fig. s3). This missense mutation site and 21 additional most differentiated SNPs between the Tibetan and control populations (*F*_ST_ > = 0.4, red box in Supplementary Fig. 2A) were used to perform haplotype analysis. We discovered that Haplotype III (containing the Lys561Arg mutation) was observed only in domestic pigs and showed an increased frequency (frequency = 0.4) in the Tibetan pigs (Supplementary Fig. s2B,C). The variants of *THSD7A* might have assisted Tibetan pigs to overcome the effects of hypoxia during pregnancy^32^.

Many of the genes related to spermatogenesis (*CLGN*, *RFX4*, *MORC1*, *TXNDC8*, *GGN*, *CATSPERG*, *DPPA4*, *DHX36*, *PPP1R2*, *GALNT3*, *NRG3*, *DKK3*) were also identified in sweep regions of Tibetan pig genomes, that might have aided counteracting deleteriously hypoxic effects in reproduction process^33,34^. *CLGN* (Calmegin) and *RFX4* (Regulatory factor X, 4) are involved in the spermatogenesis process^35,36^, and both were the only genes found in a long sweep regions (*CLGN*: Chr8: 92,110,001–92,230,000, 120 kb; *RFX4*: Chr5: 13,450,001–13,540,000, 90 kb) with very strong *F*_ST_ and XP-EHH signals (Fig. 2A,B, Supplementary Table s4 and s5).

The hypoxia-inducible factor (HIF), including HIF1 and HIF2, signaling pathway plays an essential role during the response to hypoxia in many organisms^37,38^. We found that multiple genes from the HIF pathways (including 12 candidate PSGs: *EPAS1*, *HIF1A*, *PRMT1*, *PIK3R2*, *FRAP1*, *RNF7*, *RNF4*, *EIF4E*, *TXN*, *SPSB2*, *CUL5*, and *NOX4*) were under selection in the Tibetan pigs (Fig. 2A,B, Supplementary Fig. s4). HIFs are heterodimers transcriptional factors, composed of a distinct oxygen-sensitive α subunit and a constitutive β subunit^39^. While HIF-α proteins (encoded by *HIF1A* or *HIF2A*) are constitutively synthesized and degraded under normoxic conditions, hypoxia inhibits α protein degradation by various genes regulation^40–42^. The 12 candidate PSGs (observed in our findings) mainly regulated the stability of HIF-α under different steps (gene names in red in Supplementary Fig. s4). Finally, HIFs activates the transcription of a series of genes, to increase oxygen delivery and reduce oxygen consumption, by recognizing and binding to hypoxic response elements (HREs) in the promoters of these genes^43,44^.

### Enrichment distribution of variants with different divergence levels

In our observation, no genetic differentiated (*F*_ST_ > = 0.05) missense variant was identified from 91 of the total 104 selective sweeps (*F*_ST_ analysis) in the Tibetan pig genomes, indicating that coding variants might have not accounted for majority of selective sweeps. Previous reports have implied an role of regulatory elements in pig domestication^45–47^. This raises an interesting question on the role of noncoding variants from the selective sweeps in Tibetan pig genomes. To analyze the role of coding and noncoding variants during the evolution of Tibetan pig genomes, we performed enrichment analysis of variants with different divergence levels between Tibetan and lowland pigs at the whole genome and within-sweep levels.

**Figure 4:**
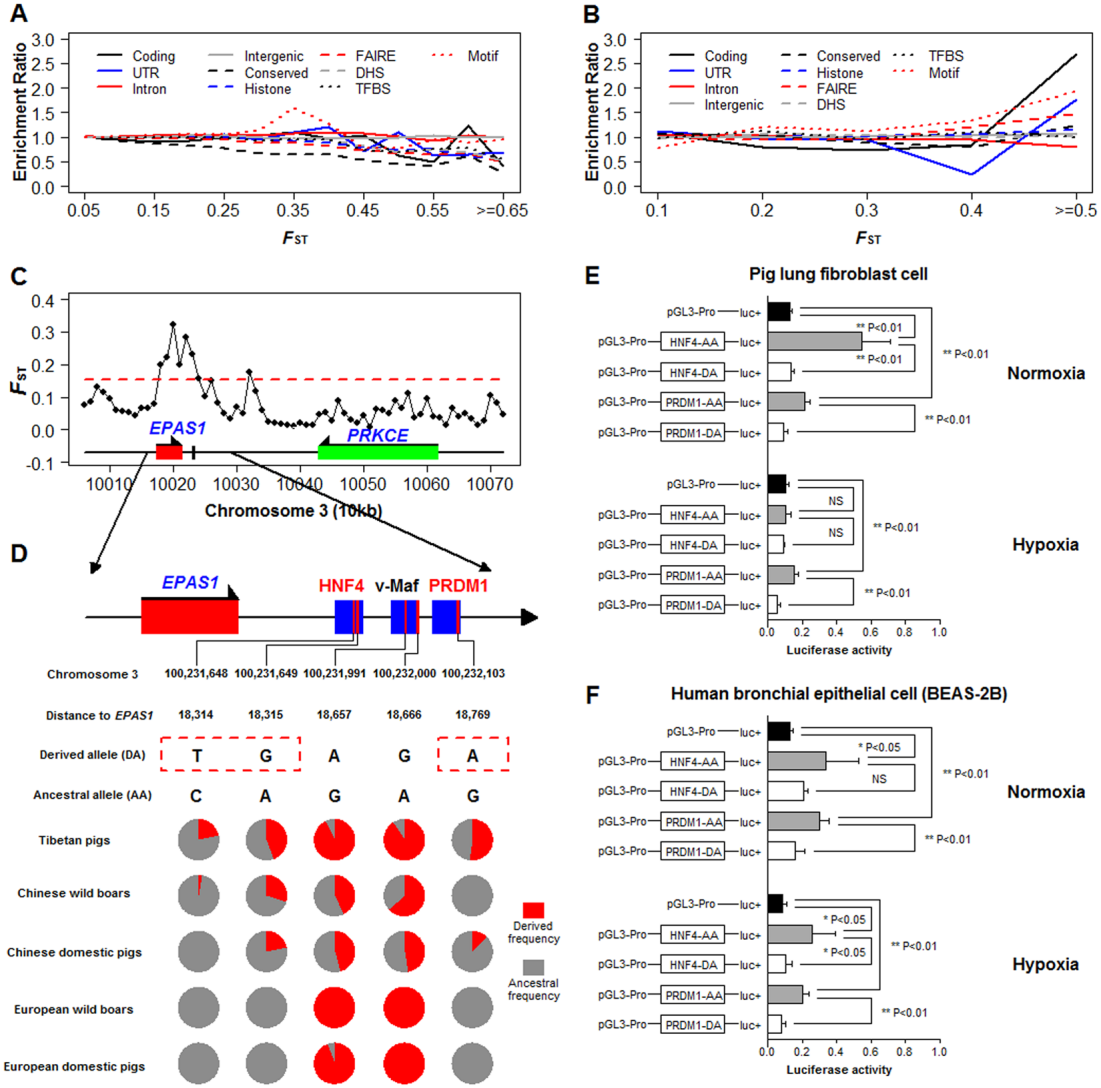
Enrichment of variants under different divergence levels and transcription activity assay of alleles in predicted motifs near *EPAS1*. (**A**) Enrichment pattern of SNPs at whole genome level. SNPs were divided to different bins by *F*_ST_ between Tibetan and lowland pigs. (**B**) Enrichment pattern of SNPs from selected sweep regions in Tibetan pigs. (**C**) Selective signals of *EPAS1*. The black vertical bar in downstream of *EPAS1* indicated differentiated SNP positions between Tibetan and lowland pigs. The red dotted line represents top 99% threshold of *F*_ST_ at whole-genome level. (**D**) Predicted motifs with differentiated SNPs in downstream of *EPAS1*. (**E**) Transcription activity assay of different alleles within predicted motifs near *EPAS1* in pig lung fibroblast cell. AA names of different pGLS3 vectors means ancestral allele and DA means derived allele. (**F**) Transcription activity assay of different alleles within predicted motifs near *EPAS1* in BEAS-2B cell. The two-tailed t test was used for statistical assessment of transcription activity change.

We first analyzed the enrichment pattern of 13,461,622 autosomal SNPs in Tibetan pig genomes. SNPs were classified into coding and different noncoding categories for enrichment analysis and linear regression analysis was used to measure the relationship between enrichment ratio and *F*_ST_ value (see Materials and Methods section for details). We found that enrichment ratios for SNPs from all functional regions decreased along with increasing differentiation levels (Fig. 4A), indicative of evolution under purifying selection. SNPs from “UTR”, “Conserved”, “Histone”, “FAIRE”, “DHS” and “TFBS” showed statistically significant negative correlation with *F*_ST_ order (Supplementary Table s8). However, the enrichment ratios for intergenic and intronic SNPs were not related to *F*_ST_ value and remained at a near constant level that centered at 1 (Fig. 4A and Supplementary Table s8), indicating a process of neutral evolution. The pattern of enrichment ratio decrease with increasing *F*_ST_ order for variants from functional regions implied that functional variants at the whole genome level have evolved under strong functional constraints and have experienced purifying selections during the evolution of Tibetan pigs.

To compare the enrichment pattern of variants from selective sweeps and the whole genome, we conducted the enrichment ratio analysis on SNPs from selective sweeps in Tibetan pig genomes. In total, we obtained 16,140 SNPs from 4.68 Mb sweep regions in *F*_ST_ outlier windows. Interestingly, the related pattern between enrichment ratio and *F*_ST_ value for SNPs in selective sweeps is contrary to the pattern observed in whole genome level (Fig. 4B). We found that the enrichment ratios showed positive correlation with *F*_ST_ order for all SNPs but SNPs from Intron. Here, only enrichment ratio for SNPs from “Motif” show statistically significant positive correlation with *F*_ST_ order and a high coefficient (*P* = 0.03; coefficient = 2.44) (Supplementary Table s8). Furthermore, the enrichment ratio of “Coding” and “UTR” increase dramatically when *F*_ST_ increase from 0.4 to > = 0.5, implying an important role of SNPs from “Coding” and “UTR” among the highly differentiated SNPs in Tibetan pig. The enrichment of highly differentiated nocoding-regulatory SNPs in selective sweeps indicated that the evolution of Tibetan pigs was related to a selection against regulatory SNPs, especially for SNPs from transcription factor (TF) recognizing motifs (“Motif”).

### Functional analysis of regulatory variants in selective sweeps of Tibetan pigs

The enrichment of highly differentiated mutations in transcription factor (TF) recognizing motifs from sweep regions may imply their important role during high-altitude adaptation in Tibetan pigs. TF recognizing motifs are important for transcriptional regulation^48^. Variants in these TF recognizing motifs from selective sweep regions in Tibetan pigs might have affected the binding affinity of transcription factors and altered gene expressional regulation. In total, 78 mutations from 97 TF recognizing motifs were detected in 4.68 Mb *F*_ST_ selected sweep regions in Tibetan pigs, and about 44 TF recognizing motifs contain SNPs showing high level of differentiation (*F*_ST_ > 0.15) between high-altitude Tibetan pigs and lowland pig populations (Supplementary Table s9). To examine the role of highly differentiated mutations in the TF recognizing motifs, we presented two cases by analyzing their genotypes and compared transcriptional activity difference between ancestral and derived alleles in the gene expressional regulation.

At first, we focused on the *EPAS1* locus since previous researches had revealed that selection on this gene is associated with high altitude adaptation in a variety of animal species^49,50^ and has also experienced strong selection in Tibetan pigs from our observation (Fig. 4C). In our analysis, no differentiated (*F*_ST_ > 0.05) missense mutation was observed in the *EPAS1* locus in Tibetan pigs. By aligning to the human orthologous sequence, we detected a clustering of three predicted TF recognizing motifs with five highly differentiated SNPs (*F*_ST_ > = 0.27) in 465 bp noncoding DNA fragment (chr3: 100, 231, 640–100, 232, 104) at 18.3 kb downstream of the *EPAS1* locus (Fig. 4D). The TF recognizing motif cluster was located in the *EPAS1* selective sweep region. The three TF recognizing motifs were predicted as the binding sites for transcription factors hepatocyte nuclear factor 4 (HNF4), V-maf musculoaponeurotic fibrosarcoma oncogene homolog (v-Maf), PR domain containing 1 (PRDM1), respectively (Fig. 4D). HNF4 is a hypoxia-responding transcription factor interacting with HIF-1 (hypoxia-inducible factor 1) to regulate the expression of erythropoietin (Epo) to compensate for reduced oxygen supply to organs by modulating erythropoiesis under hypoxia^51^. PRDM1 is a plasma cell-specific transcription factor and its expression decreased under hypoxia condition^52^. To reveal the linkage disequilibrium in *EPAS1* sweep, we combined the five differentiated SNPs from TF recognizing motifs and 40 most differentiated SNPs from *EPAS1* to analyze the haplotype structure in all Eurasia samples. We discovered that the haplotype XXV of *EPAS1* was linked to the derived alleles of all the five SNPs and this haplotype was only presented in Tibetan pigs (Supplementary Fig. s5). We further analyzed the derived allele frequency of these SNPs in all the 227 Eurasia pigs. We found that the derived alleles of the three SNPs among recognizing motifs of HNF4 and PRDM1 were observed only in Asian pig population and showed increased frequency in Tibetan pig (Fig. 4D), suggesting an important regulatory role for high-altitude adaptation of Tibetan pig.

To investigate regulatory effects on HNF4 and PRDM1 recognizing motifs in downstream of *EPAS1*, we clone ancestral and derived HNF4 and PRDM1 motifs (Supplementary Table s9) into luciferase reporter plasmids to test their transcriptional regulatory activity under different level of oxygen concentration (21% and 2%). After transfection into the pig lung fibroblast cell line (21% oxygen), both ancestral-type vectors (HNF4-AA and PRDM1-AA) show statistically significant increase in luciferase expression when compared with the empty vector (pGL3-promoter) implying enhancer activity of the two predicted TF recognizing motifs (Fig. 4E). However, a reduction of transcriptional activity was observed for both derived-type HNF4-DA and PRDM1-DA motifs when compared to their ancestral-types (Fig. 4E). We also investigated the luciferase activity of differentiated SNPs in HNF4-DA and PRDM1-DA reporter vectors after hypoxia incubation (2% oxygen) in 48 h. We found that the two ancestral reporter vectors showed increased luciferase expression (Fig. 4E). Interestingly, both derived-type HNF4-DA and PRDM1-DA motifs also showed decreased luciferase expression as compared to corresponding ancestral-types in hypoxia experimental replicates. Similar results were also observed in human bronchial epithelial cell line (BEAS-2B) (Fig. 4F). In a recent report, a down-regulated expression of *EPAS1* was detected in association with high altitude adaptation in Tibetans, implying that the adaption could be due to selection for a change in *EPAS1* expressional level^47^. Our experimental data indicated that the mutations in the *EPAS1* downstream noncoding regulatory sequence could have affected gene expression and have been putatively involved in the high altitude adaptation in Tibetan pigs.

We also detected another cluster of three TF recognizing motif cluster with three highly differentiated SNPs (*F*_ST_ > = 0.4) within a 408 bp fragment (chr2: 61,358,862 –61,359,269) in the immediate upstream (123 bp) of *CYP4F2* transcription start site (Supplementary Fig. s6A,B and Table s9). This gene encodes an omega-hydroxylase and synthesizes 20-hydroxyeicosatetraenoic acid (20-HETE) which plays an important role in blood pressure control^53,54^. Three TF recognizing motifs were predicted as the binding sites for transcription factors forkhead box protein A (FOXA), specificity protein 1 (SP1), nuclear factor erythroid 2 (NFE2), respectively. NFE2 is essential for regulating erythroid and megakaryocytic maturation and differentiation^54,55^, and mutation of NFE2 was associated with polycythemia^56^. The functional convergence of NFE2 and *CYP4F2*, and the observation of NFE2 binding site in the *CYP4F2* promoter region implied an expressional regulation of *CYP4F2* by NFE2. The derived alleles of three SNPs were also observed in Chinese wild boars with high frequency, implying their origin from wild boar (Supplementary Fig. s6B).

To test the mutation effect on the NFE2 recognizing motif in *CYP4F2* promoter, we conducted an experimental assay to test the transcriptional activity of NFE2 recoginizing motifs (Supplementary Table s9). We compared the luciferase activity of ancestral and derived NFE2 motifs under normoxia (21%) and hypoxia (2%) conditions. pGL3-Basic vector with ancestral/derived NFE2 motif fragments inserted in the promoter region were transfected into pig lung fibroblast and BEAS-2B cells. Anestral-type (NFE2-AA) showed statistically significant increase (P < 0.01) in luciferase expression when compared to the empty vector (pGL3-basic) in all cell lines and oxygen condition (Supplementary Fig. s6C,D), implying that the NFE2 motif is a promoter sequence for the *CYP4F2* gene. Furthermore, we found that the activity of derived-type vectors (NFE2-) decreased compared to ancestral-type in all conditions (Supplementary Fig. s6C,D). This result implied that the mutation in the NFE2 motif may have altered the promoter activity of *CYP4F2* resulting in different *CYP4F2* expression, which might have played a role in high-altitude adaptation in Tibetan pigs.

## Discussion

Clarifying history of the origin of Tibetan pigs is critical in guiding investigation of the genetic mechanism underlying their high-altitude adaptation and resolving previous controversy about their origin. In this study, we analyzed the genomic variants in 229 pig genomes across Eurasia through phylogenomic and population structure approach. We discovered that Tibetan pigs had a similar genetic structure with domestic pigs from northern group. Genome comparison between Tibetan pigs and low-land domestic pigs from northern group, unveiled signatures associated with high-altitude adaptation in Tibetan pig genomes. Furthermore, we also revealed the important role of noncoding regulatory SNPs in high-altitude adaptation of Tibetan pigs.

Our results suggests Tibetan pigs possibly share a common ancestor with other domestic pigs from low-land regions in north of Nanling Mountains. Phylogenomic and population structure analyses of whole genome variants revealed that Tibetan pigs showed close phylogenetic relationship and similar genetic background with other domestic pigs from low-land areas in north of Nanling Mountains, all of which diverged from wild boars from different regions of China and domestic pigs in south of Nanling Mountains. The genetic structure of Tibetan pigs observed from nuclear genomic analyses in our study is in accordance with the mtDNA pattern previously observed in Wu *et al*.’s report^13^. The Tibetan pigs phylogenetically clustered with domestic pigs from northern group, rather than with East Asian wild boars, indicating the less possibility of Tibetan pigs beings wild as previously reported by Li *et al*.^12^, and the less likelihood to be domesticated from Tibetan wild boars, based on evidences from partial mtDNA sequences, as an event paralleling to the domestication in low-land regions^14^. We inferred that the inconsistency between the whole genome and mtDNA analysis may possibly be due to partial maternal introgression of wild boars into Tibetan pigs that were included in Yang *et al*.’s. study, since the partial mtDNA sequence could provide only limited information. Our analysis confirmed close relationship between Tibetan and neighboring low-land pig populations from the chip data analysis^15^.

Tibetan pigs may have moved to Qinghai-Tibetan Plateau from middle Yellow River basin. Recent study based on ancient mtDNA found pig remains from middle Yellow River region (including the earliest archaeological sits of pigs, about 10,500–7,575 before present) contained the mtDNA haplotypes (haplotypes H2, H3, H4 and H10) that are dominant in both younger archaeological and modern populations and was thought as one of the centers for early Chinese pig domestication^57^. By further analyzing this report, we found that mtDNA haplotypes H2, H3, H4 and H10 also accounted for 296 of the 348 sequences in modern Tibetan pigs. Furthermore, domesticating or breeding pigs only might have happened after the shift in life style from hunter-gatherer to sustained settlement and development of agriculture. Pigs are not so skilled at migrating but usually captive fed, and relies upon humans for food. The earliest archaeology records of dometic plants and pigs in middle Yellow River region (Nanzhuangtou sit, about 10,500–9,700 years ago)^58^ is mucher earlier than that in Tibet (Karuo site, about 4,300–4,700 years ago)^59^. Tibetan began to plant millets and settle down about 5,200 years ago^60^. Thus, combining evidences from both our genetic analysis and previous archaeological reports, we hypothesized that Tibetan pigs were transported to the Qinghai-Tibetan Plateau from middle Yellow River region less than 5,200 years ago.

The PSGs identified (n = 273) in this analysis showed only a few overlaps with previous researches by Li *et al*.^12^ (Li *et al*., 2013) and Ai *et al*.^15^. We discovered that many missense mutations in the positively selected genes reported by Li *et al*. (2013) showed similar allele frequency in Tibetan pigs and low-land pigs in China (data not shown), indicating that these missense mutations were not specifically related to the evolution of Tibetan pigs. Furthermore, our results also have little common PSGs with Ai’s candidate gene list due to low SNP density and design bias of illumina porcine SNP60 chip. We found that illumina porcine SNP60 chip only covered 75 of 93,789 SNPs located in selected sweep regions of Tibetan pigs in this study, as the illumina porcine SNP60 chip was designed by SNPs from European pig populations^61^.

Our study revealed the genetic mechanism underlying the high-altitude adaptation in Tibetan pigs. PSGs involved in different physiological functions might have collaborated in aiding Tibetan pigs to overcome different physiological pressure caused by high-altitude environment, such as hypoxia, UV damage and impaired reproduction^62^. 12 PSGs related to HIF pathways were identified under selection in Tibetan pigs, indicative of the important role of HIF pathways in hypoxia adaptation process of Tibetan pigs. Here, the most common gene, *EPAS1*, was also detected in a sweep region with strong selection signal in Tibetan pigs. We found a specific medium-frequency haplotype XXV of *EPAS1* in Tibetan pigs. Further linkage disequilibrium analysis shows that haplotype XXV was linked to five derived-alleles in downstream of *EPAS1*, three of which has been proven to have repressed regulation activity. Recently, Peng *et al*. discovered an *EPAS1* adaptive haplotype in Tibetans down-regulates *EPAS1* transcription, which contributed to the genetic adaptation of Tibetans to high-altitude hypoxia^50^. Our results implied a similar adaptive molecular mechanism in *EPAS1* between Tibetans and Tibetan pigs. Furthermore, we also discovered an elevated derived-missense mutation (Lys561Arg) on a very conserved site of *THSD7A* in Tibetan pigs. *THSD7A* is involved in endothelial cell migration and embryonic angiogenesis^30,31^ and this missense mutation might have assisted Tibetan pigs to overcome impaired reproduction caused by high-altitude hypoxia.

Our analysis revealed an important function of highly differentiated regulatory SNPs between high-altitude Tibetan pigs and lowland pig populations during the evolution of Tibetan pigs. From whole genomic level view, highly differentiated regulatory SNPs have evolved under strong evolutionary constraints (purifying selection), possibly due to having important biological regulatory function. Comparatively, the enrichment of highly differentiated regulatory SNPs in selective sweeps of Tibetan pigs indicated that the selective sweeps were possibly due to positive selection against these regulatory SNPs. Since much larger number of regulatory SNPs than protein coding SNPs were observed in the selective sweeps, the regulatory SNPs might have played more role during the high altitude adaptation in Tibetan pigs as compared to the protein coding SNPs. From our observation, the sweeps in *EPAS1* and *CYP4F2* could be associated with regulatory SNPs at downstream of *EPAS1* and the promoter sequence of *CYP4F2*. Inferring from our observation, the regulatory variants might be associated with an important role during high-altitude adaptation in Tibetan pigs and more attention should be paid on it in future while studying evolution in other animals.

## Material and Methods

All methods were performed in accordance with the guidelines approved by the Kunming Institute of Zoology, Chinese Academy of Sciences. All experimental protocols were approved by the Kunming Institute of Zoology, Chinese Academy of Sciences.

### Sample collection and data download

Ear or muscle tissues from 48 samples (including wild boars and domestic pigs) from China (Supplementary Table s1) were collected and kept in 95% alcohol. Among this, 11 Tibetan pigs were sampled from the Qinghai-Tibet Plateau (average altitude >3,000 m, see Fig. 1A), and nine wild boars were collected from geographically isolated locations in China. The remaining 28 domestic pigs (average of altitude <600 m) comprises of 11 different pig breeds from China (Supplementary Table s1). We then downloaded genomes of 182 Eurasian pigs (including 138 Chinese samples and 44 European pigs) and six samples as outgroup (Supplementary Table s1) from the Sequence Read Archive (SRA) database (http://www.ncbi.nlm.nih.gov/Traces/sra/).

### Whole genome Re-sequencing, read mapping and SNP calling

The 48 pigs were used for the whole genome resequencing. Genomic DNA was extracted using a routing phenol-chloroform method and precipitated by 75% alcohol. The resequencing libraries were constructed with 500-bp inserts according to the Illumina library construction protocols. 100-bp paired-end reads were generated with the HiSeq2000 platform (Illumina).

To obtain reliable alignment results, low-quality sequences (phred quality score <20) from all data sets were trimmed by QcReads (http://sourceforge.net/projects/qcreads/). The controlled genomic reads were then mapped to the Duroc reference genome (NCBI build *Sscrofa* 10.2) with the BWA program^63^ (http://sourceforge.net/projects/bio-bwa/). Before SNPs calling, SAMtools^64^ was used to sorting, merging and removing PCR duplicates generated during genomic library construction. To ensure reliability of the downstream analyses, we selected only sequences with mapping quality greater than 20 for SNP calling. To call high quality SNPs, a high consensus quality (≥20) or a high SNP quality (≥20) required if homozygous at a genomic site; and an intermediate criterion (consensus quality≥10 and SNP quality≥10) required if heterozygous. SNPs of each individual were called using SAMtools. The sequence reads are available at GSA (Genome Sequence Archive) under accession CRA001606.

### Population genetic analysis

To exclude the effect of recently intercontinental gene flows between Eurasia pigs, we used STRUCTURE (2.3.4) to analyze the population structure of all the 233 samples (K set as 3) with 3 iterations. Finally, only 98 Chinese pig samples (26 Tibetan pigs, 20 Chinese wild boars, 52 Chinese domestic pigs from 13 breeds) showing European genetic component fraction less than 5% were included in the subsequent analysis (Supplementary Table s3).

Only SNPs with good sample coverage (above 89% individual) from autosomes were used for the analyses of the genetic structure of the 98 Chinese pigs. Furthermore, the physical distance between SNPs was greater than 100 kb to avoid any bias due to linkage disequilibrium (LD)^65^. Finally, 22,664 SNPs passed quality control test and were used for the population genetic analysis. Two wild boars from Sumatra, Indonesia, were used as the out-group. For constructing the Neighbor-Joining (NJ) phylogenetic tree, the miss ratio of the SNPs of each individual is less than 10% (80 out of the 98 Chinese pig qualified for this condition). MEGA6^20^ was used to construct the NJ tree. STRUCTURE 2.3.4^18^ was used to infer the population structure within the different Chinese pig populations with different K values (from 2 to 5). Principal component analysis (PCA) was accomplished with R language (http://www.r-project.org/).

### Selective sweep analysis

The fixation index (*F*_ST_)^66^ was used to measure the population differentiation between 26 Tibetan pigs and 29 Chinese low-altitude control pigs. Previous reports indicate that the pig ancestor emerged from South East Asia 5.3-3.5 Myr ago^61^. In addition, the genetic background of the wild boar from Sumatra is more similar to the Eurasian pigs than to *Sus cebifrons*, *Sus celebensis*, *Sus verrucosus* and *Sus barbatus* (Supplementary Fig. s1 and Table s3). Therefore, the wild boar from Sumatra in Indonesia is more suitable for defining the derived alleles within domesticated pigs. Only sites that were homozygous in two Sumatra wild boars were defined as the ancestral allele. First, 33,432,165 SNPs were called in the autosomes of 26 Tibetan pigs and 29 control pigs. Next, 28,283,678 SNPs were found to be homozygous in the two Sumatra wild boars. The allele frequencies within each population were calculated with a correction for sample size and sequencing depth at each SNP site (details described in supplementary note) and then used for the *F*_ST_ analysis. The range of *F*_ST_ is between 0 and 1. However, it is possible to get negative values, which have no biological interpretation; therefore, negative values were set to 0. Phased SNPs are needed for the XP-EHH analysis between 26 Tibetan pigs and 29 control pigs. 7,408,187 SNPs with good sample coverage (Tibetan pigs > = 24, control pigs > = 25) in the autosomes were phased by the program fastPHASE^67^ (http://stephenslab.uchicago.edu/software.html). Cross Population Extended Haplotype Homozogysity (XP-EHH) values for each SNP were calculated by the XP-EHH program (http://hgdp.uchicago.edu/Software/). Sliding windows were used for both the *F*_ST_ and the XP-EHH analyses to avoid the influence of genetic drift. As the extent of LD is no more than 10 kb in Chinese domestic pigs^68^, the window size was set as 10 kb. Average values were calculated for all SNPs within each sliding window. Regions with a clustering of at least three consecutive (except undetermined genomic gaps) sliding windows above the genome-wide top 1% of *F*_ST_ or XP-EHH values were defined as selected sweeps.

### Enrichment analysis of differentiated variants in Tibetan pigs

The SNPs were classified into different categories, coding, UTR, intronic, and intergenic, to compare their differentiation patterns. We also classified the SNPs into different regulatory categories by annotation of homologous sequence to human in our other unpublished work, such as transcription factor binding sites (“TFBS”), transcription factor recognizing motifs (“Motif”), DNase I hypersensitive sites (“DHS”), Formaldehyde-Assisted Isolation of Regulatory Elements (“FAIRE”), and histone chemical modification sites (“Histone”). Furthermore, conserved sequence (phastCons score > = 0.2) among 100 vertebrate genomes from UCSC database (http://genome.ucsc.edu) were also included in our analysis. The SNPs were divided into different subsets by *F*_ST_ values. An enrichment ratio was developed to measure the distribution of different categories of SNPs in *F*_ST_ bins that were ordered by increasing levels of genetic differentiation. For a specific group of SNPs in a *F*_ST_ bin, the enrichment ratio was denoted as the ratio of the number of observed SNPs (P_observed_) to the number of SNPs expected under random (P_expected_). A P_observed_/P_expected_ ratio greater than 1 indicates an enrichment of SNPs in a *F*_ST_ bin, while a ratio less than 1 indicates a deficiency of SNPs in the *F*_ST_ bin.

### Cell culture

Pig lung fibroblast cell line was purchased from the Kunming Cell Bank, Kunming Institute of Zoology, Chinese Academy of Sciences (Kunming, China). Human bronchial epithelial cell line (BEAS-2B) was obtained from the Yunnan University (Kunming, China). Cells were cultured in DMEM (Gibco, New York, USA) supplemented with 10% fetal bovine serum (Gibco, New York, USA), 100 U/mL penicillin, 100 μg/mL streptomycin (Beyotime Biotechnology, Hangzhou, China), and incubated at 37°C and 5% CO_2_.

### Plasmids construction and luciferase assays

DNA fragments (50 bp: chr2: 61,359,235-61,359,284; 49 bp: chr2: 61,368,465-61,368,513; 49 bp: chr3: 58,229,710-58,229,758; 50 bp: chr3: 100,231,621-100,231,670; 50 bp: chr3: 100,232,077-100,232,126) containing ancestral-type or derived-type Motif were synthesized and cloned into pGL3-basic or pGL3-promoter vector (Promega) in *BamH*I and *Sal*I digestion sites. Each construct was confirmed by sequencing.

For luciferase assays, cells were seeded onto 24-well plates corresponding to ∼80% confluency. Cells were transfected with various reporter construct along with pRL-TK Renilla luciferase plasmid (Promega) by using Lipofectamine 3000 Reagent (Invitrogen) according to the manufacturer’s instructions. For hypoxic assays, cells were incubated in an atmosphere of 2% O_2_, 93% N_2_ and 5% CO_2_ after transfection. After forty-eight hours, cells were collected for luciferase activity assays using the Dual-Luciferase Reporter Assay System (Promega) according to the manufacturer’s instructions. Luminescence signals were captured in a Varioskan Flash Multimode Reader (Thermo Scientific). Firefly signals were normalized with the Renilla luciferase internal control. Six independent transfection and assays were performed. *P*-values between ancestral-type and derived-type Motif were calculated using the two-tailed *t*-test.

## Supplementary information

Supplementary information
