# Supplementary material for: Population Genomics Analysis Revealed Origin and High-altitude Adaptation of Tibetan Pigs
Source: Sci Rep. 2019 Aug 7;9:11463. doi: 10.1038/s41598-019-47711-6 (PMC6685962; doi:10.1038/s41598-019-47711-6)
Supplement: Supplementary file 1 — Supplementary information [file 41598_2019_47711_MOESM1_ESM.docx]

**Article: Discoveries**

**Population Genomics Analysis Revealed Origin and High-altitude Adaptation of Tibetan Pigs**

Yun-Fei Ma^a,b,c,1^, Xu-Man Han^a,1^, Cui-Ping Huang^a,b,c^, Li Zhong^a,d^, Adeniyi C. Adeola^a^, David M. Irwin^a,e^, Hai-Bing Xie^a,1,^*, Ya-Ping Zhang^a,d,^*

1. State Key Laboratory of Genetic Resources and Evolution, and Yunnan Laboratory of Molecular Biology of Domestic Animals, Kunming Institute of Zoology, Chinese Academy of Sciences, Kunming 650223, China
2. Kunming College of Life Science, University of Chinese Academy of Sciences, Kunming 650204, China
3. University of Chinese Academy of Sciences, Beijing 100049, China
4. Laboratory for Conservation and Utilization of Bio-resource, and Key Laboratory for Animal Genetic Diversity and Evolution of High Education in Yunnan Province, Yunnan University, Kunming 650091, China
5. Department of Laboratory Medicine and Pathobiology, University of Toronto, Ontario M5S 1A8, Canada

^*^Corresponding authors. Tel: +86 871 6852 6518; Fax: +86 871 6852 6519

E-mail adresses: [xiehb@mail.kiz.ac.cn](mailto:xiehb@mail.kiz.ac.cn) (Hai-Bing Xie); [zhangyp@mail.kiz.ac.cn](mailto:zhangyp@mail.kiz.ac.cn) (Ya-Ping Zhang).

^1^These authors contributed equally to this work.

**Table of contents**

**Supplementary notes**

**Supplementary Fig. s1-6**

**Supplementary Fig. s1:** Population structure analysis of all the 233 samples in this study.

**Supplementary Fig. s2:** Selective signal and haplotypes of *THSD7A*.

**Supplementary Fig. s3:** Partial protein sequences encoded by *THSD7A* in different vertebrates.

**Supplementary Fig. s4:** Candidate PSGs involved in the HIF signaling pathway.

**Supplementary Fig. s5:** Haplotype pattern of 45 differentiated SNPs between the Tibetan and control pig populations using 227 pigs from East Asia and Europe.

**Supplementary Fig. s6:** The position and transcription activity assay of alleles within predicted motifs in upstream of *CYP4F2*.

**Supplementary Tables s1-s9**

**Supplementary Table s1:** Information on samples and sequencing.

**Supplementary Table s2:** Information for samples in this study.

**Supplementary Table s3:** Average genetic components of all the 233 samples.

**Supplementary Table s4:** Genetic regions under selection in the Tibetan pig by *F*_ST._

**Supplementary Table s5:** Genetic regions under selection in the Tibetan pig by XP-EHH.

**Supplementary Table s6:** List of 237 candidate PSGs.

**Supplementary Table s7:** Classification of physiological functions of some of the PSGs.

**Supplementary Table s8:** Related analysis between enrichment ratio and *F*_ST._

**Supplementary Table s9:** Predicted Motifs in selected sweep regions of Tibetan pigs.

**Supplementary Note:**

**Allele frequencies estimation**

To obtain reliable allele frequencies, we used maximum likelihood estimation to correct the sample size and sequencing depth at each SNP site.

In a population, at each SNP site, the allele frequencies are denoted as *p* (A allele) and *q* (B allele), while the sum of *p* and *q* equal to 1. We assume that the sequenced individuals are representative of the total population and that all SNP sites follow Hardy-Weinberg equilibrium. Hence, the probability to observe a genotype for an individual at a SNP site is conditional on the sequencing depth for that individual at the genomic site, and on the allele frequency in the total population. The probability can be deduced as:

*P*(*G_i_* | *p*, *q*, *D****_i_***) = $\left\{ \begin{aligned} p^{2}+2pq*\left( \frac{1}{2} \right)^{Di} , if Gi is homozygous for the A allele \\ 2pq-2*2pq*\left( \frac{1}{2} \right)^{Di} , if Gi is heterozygous \\ q^{2}+2pq*\left( \frac{1}{2} \right)^{Di} , if Gi is homozygous for the B allele \\ 1 , if Di is 0 \end{aligned} \right.$

Where *G_i_* is the observed genotype for the *i*th individual in the sequenced population; *D****_i_*** is the sequencing depth at the SNP site for the *i*th individual. The parameter $2pq*\left( \frac{1}{2} \right)^{Di}$ is the probability that a heterozygous genotype is sequenced as one homozygous given a sequencing depth *D****_i_***. For convenience in later analysis, the probability is set to 1 if the genotype is unknown when *D****_i_*** is zero.

The likelihood distribution for allele frequency *p* can be formulated as the product of the probability of the observed genotypes for the sequenced individuals:

*L*(*p*) = $\prod_{i=1}^{k} P(Gi | p, q, Di)$

Here, *k* is the sample size of the sequenced population. For different SNP sites, distinct likelihood distributions are deduced according to the observed genotypes and sequencing depths of the individuals, and the allele frequency *p* in a population.

For simplicity, we assume that the prior allele frequency *p* is uniformly distributed between 0 and 1. Therefore, the posterior distribution for the allele frequency *p* for a SNP can be deduced as:

*f*(p) = *U*(p)**L*(p)

Where, *U*(p) represents the prior uniform distribution.

The maximum likelihood estimate (MLE) of the allele frequency *p* is calculated when maximizing the *f*(p).

**Supplementary Fig. s1:** Population structure analysis of all the 233 samples in this study. K is set as 3. EWB: European wild boar, EDP: European domestic pig, CWB: Chinese wild boar, TP: Tibetan pig, CDP: Chinese domestic pig, OG: outgroup.


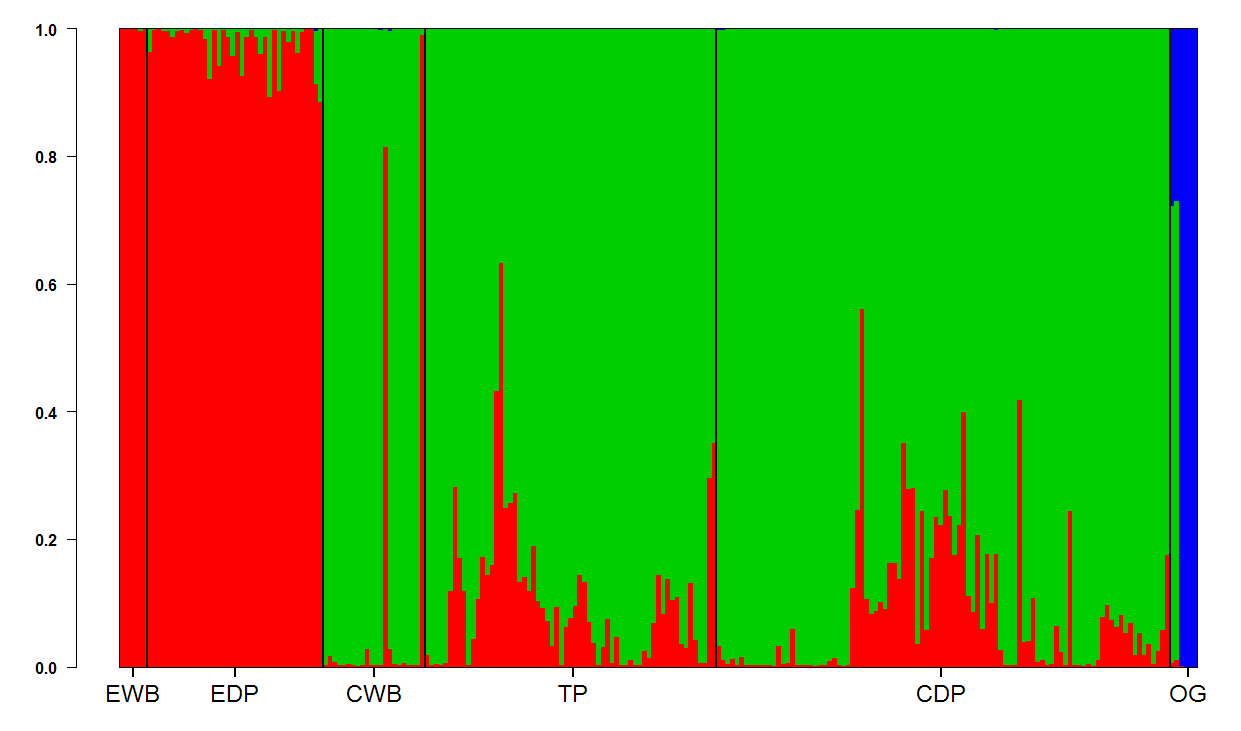


**Supplementary Fig. s2:** Selective signal and haplotypes of *THSD7A*. (*A*) *F*_ST_ values of each SNP between Tibetan and control populations. X axis is the physical position on chromosome 9 (*Sus scrofa* 10.2 build). Regions between the two green or purple dashed lines are candidate sweeps. The red box defines SNPs largest genetic differentiation, which were used to analyze the haplotypes. (*B*) Haplotype pattern of the high differentiation SNPs between Tibetan and control populations in all 227 pigs from East Asia and Europe. Each column is a polymorphic genomic location and each row is a phased haplotype. Blue cells represent ancestor alleles and red cells represent derived alleles. OG: outgroup, TP: Tibetan pig, CDP: Chinese domestic pig, CWB: Chinese wild boar, EDP: European domestic pig, EWB: European wild boar. (*C*) The Median-Joining network of the haplotypes within *THSD7A*. Only haplotypes with frequency more than 1 were used to draw the network. Asterisk (*B*, *C*) indicates position of the K516R missense mutation in *THSD7A*.


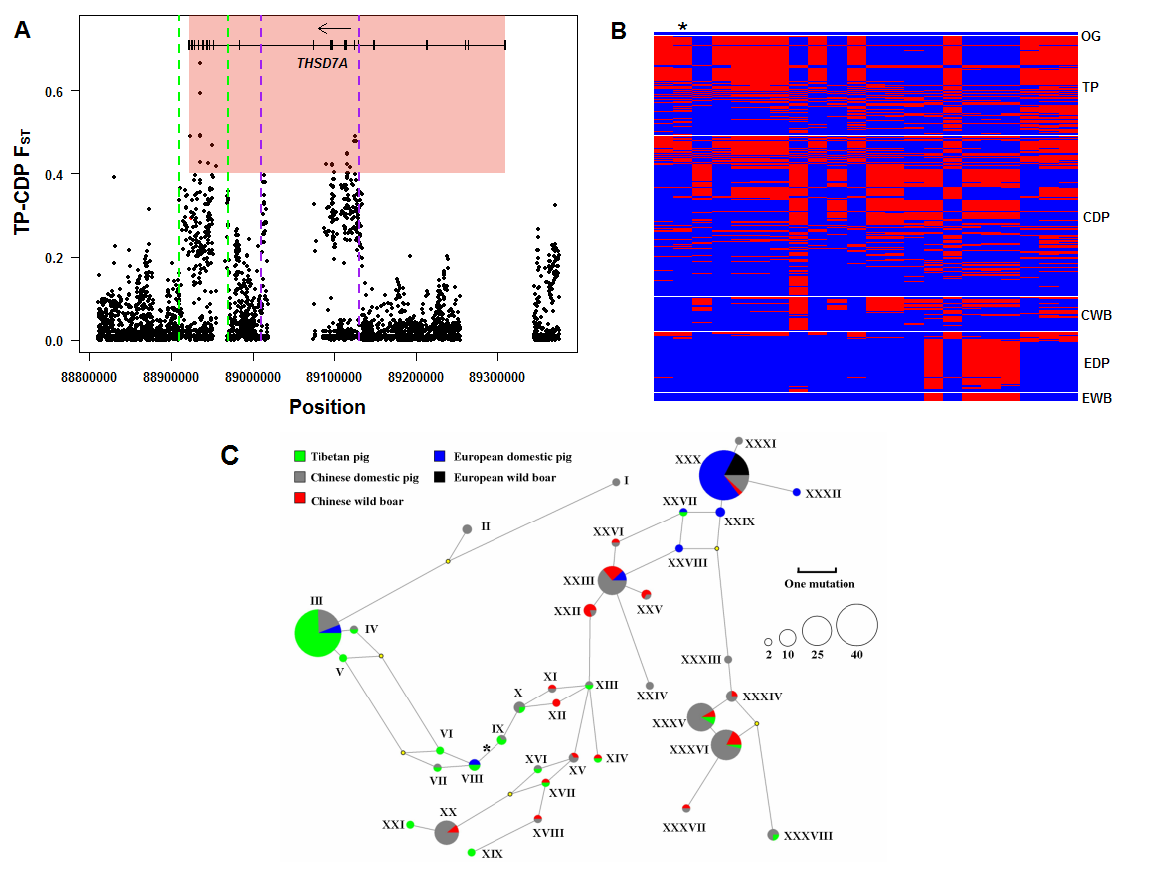


**Supplementary Fig. s3:** Partial protein sequences encoded by *THSD7A* in different vertebrates.


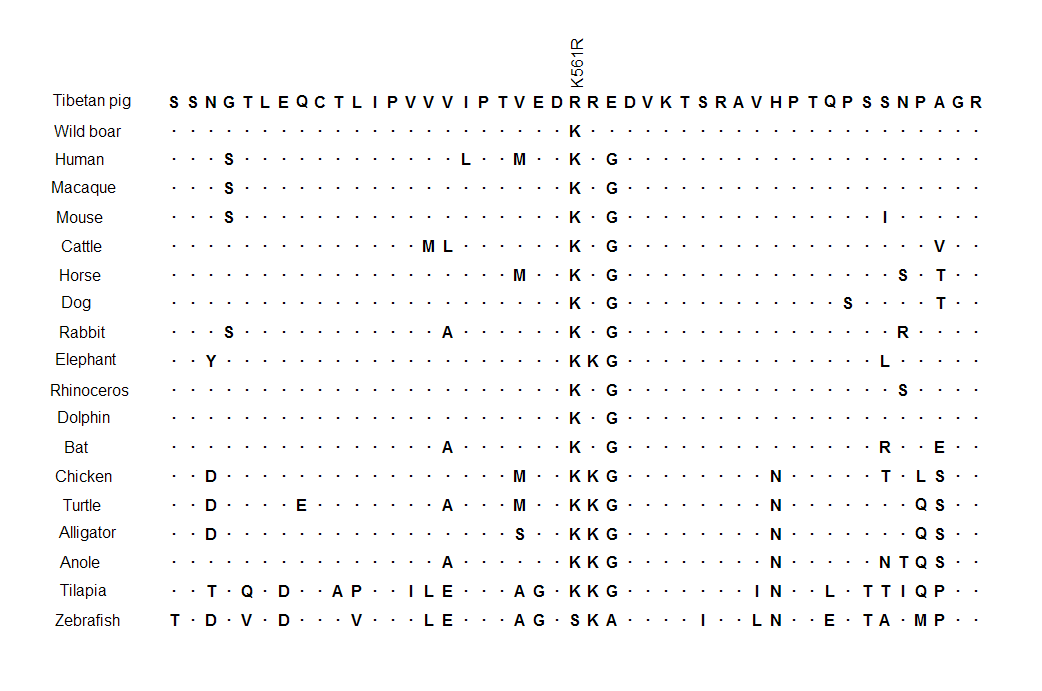


**Supplementary Fig. s4:** Candidate PSGs involved in the HIF signaling pathway. Gene names labeled in red showing evidence of selection in the Tibetan pigs

**
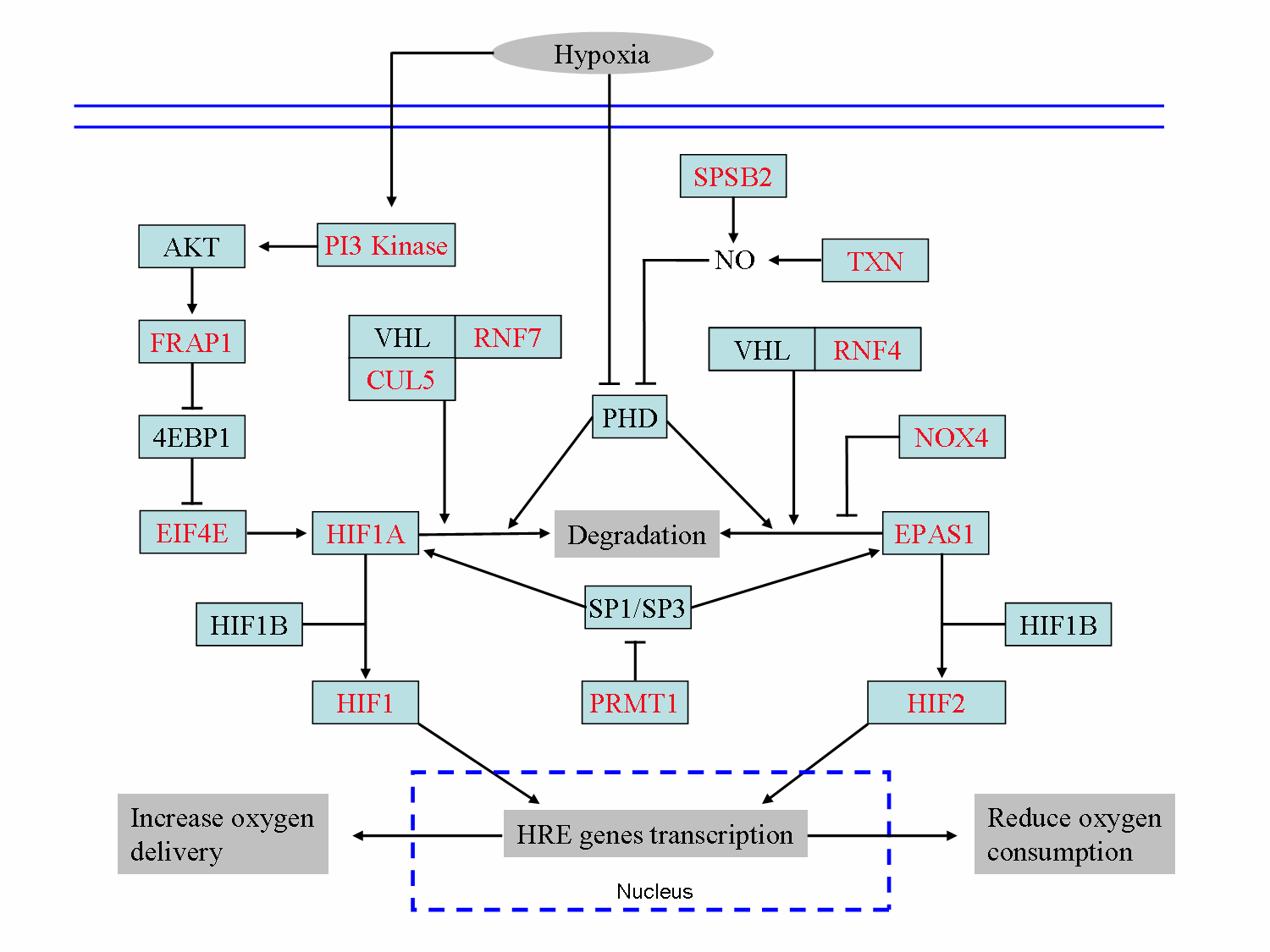
**

**Supplementary Fig. s5:** Haplotype pattern of 45 differentiated SNPs between the Tibetan and control pig populations using 227 pigs from East Asia and Europe. Each column is a polymorphic genomic location and each row is a phased haplotype. The blue cell represents the ancestor allele and the red cell represents the derived allele. OG: outgroup, TP: Tibetan pigs, CDP: Chinese domestic pig, CWB: Chinese wild boar, EDP: European domestic pig, EWB: European wild boar. 1:chr3:100,231,648; 2:chr3:100,231,649; 3:chr3:100,231,991; 4:chr3:100,232,000; 5:chr3:100,232,103.

**
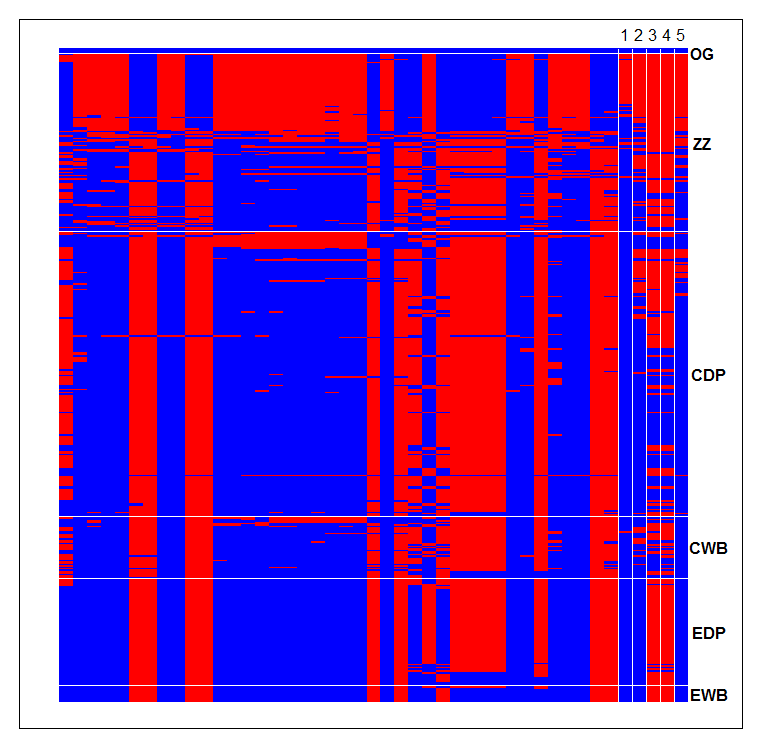
**

**Supplementary Fig. s6:** The position and transcription activity assay of alleles within predicted motifs in upstream of *CYP4F2*. (*A*) Selective signals of *CYP4F2*. The black vertical bar in upstream of *CYP4F2* indicated differentiated SNP positions between Tibetan pigs and lowland pigs. The red dotted line represents top 99% threshold of *F*_ST_ at whole-genome level. (*B*) Predicted motifs with differentiated SNPs in upstream of *CYP4F2*. (*C*) Transcription activity assay of alleles within NFE2 motif in pig lung fibroblast cell. AA in names of different pGLS3 vectors means ancestral allele and DA means derived allele. (*D*) Transcription activity assay of alleles within NFE2 motif in human bronchial epithelial cell line (BEAS-2B). The two-tailed t test was used for statistical assessment of transcription activity change.


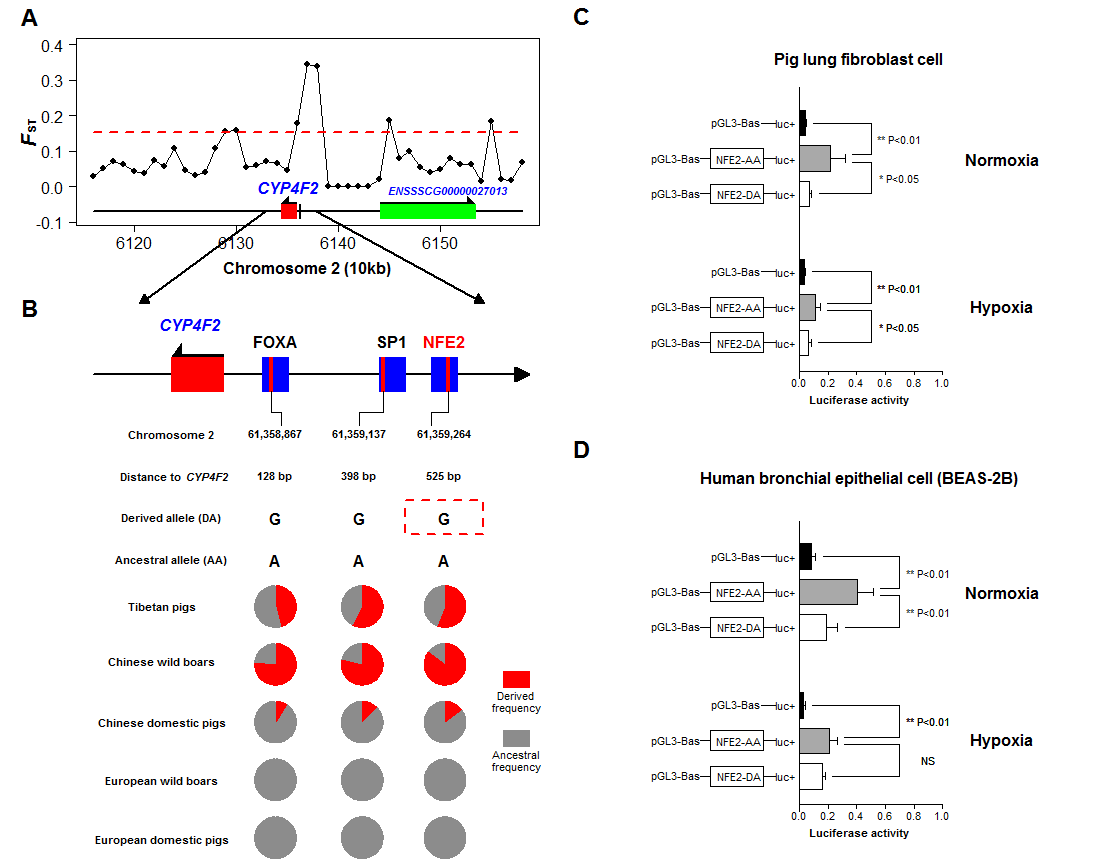


**Supplementary Table s1:** Information on samples and sequencing.

| **Group** | **Population** | **Origin** | **Average altitude (m)** | **New sequenced** | **Downloaded** | **Mapped data (Gbp)** | **Average depth (X)** | **SNP (M)** |
| --- | --- | --- | --- | --- | --- | --- | --- | --- |
| **East Asia** | Wild boar | China | 400 | 9 | 13 | 224.45 | 5.26 | 29.07 |
|  | Tibetan | Qinghai-Tibet Plateau, China | 3000 | 11 | 52 | 835.94 | 6.69 | 35.11 |
|  | Laiwu | Shandong province, China | 160 | 10 | 6 | 188.10 | 6.09 | 16.61 |
|  | Xiang | Guangxi Zhuang Autonomous Region, China | 220 | 9 | 8 | 218.04 | 6.64 | 20.90 |
|  | Rongchang | Chongqing Municipality, China | 350 | 1 | 5 | 76.24 | 6.61 | 14.40 |
|  | Jinhua | Zhejiang province, China | 260 | 1 | 3 | 30.44 | 3.99 | 8.59 |
|  | Wei | Anhui province,China | 20 | 1 | 0 | 8.65 | 4.75 | 2.28 |
|  | Wanzhe | Anhui province,China | 200 | 1 | 0 | 3.80 | 2.47 | 0.58 |
|  | Jiaxing | Zhejiang province, China | 8 | 1 | 0 | 5.87 | 3.41 | 0.84 |
|  | Large Black-white | Guangdong Province, China | 50 | 1 | 0 | 6.90 | 3.91 | 1.68 |
|  | Lantang | Guangdong Province, China | 300 | 1 | 0 | 7.06 | 3.98 | 1.76 |
|  | Hang | Jiangxi Province, China | 600 | 1 | 0 | 6.08 | 3.51 | 1.17 |
|  | Putian | Fujian province, China | 150 | 1 | 0 | 3.48 | 2.35 | 0.49 |
|  | Penzhou | Sichuan province, China | 800 | 0 | 3 | 25.78 | 4.53 | 8.77 |
|  | Ya'nan | Sichuan province, China | 800 | 0 | 3 | 25.24 | 4.45 | 5.95 |
|  | Neijiang | Sichuan province, China | 400 | 0 | 3 | 26.93 | 4.75 | 6.73 |
|  | Meishan | Jiangsu Province, China | 5 | 0 | 4 | 60.78 | 8.25 | 10.09 |
|  | Hetao | Inner Mongolia Autonomous Region, China | 1100 | 0 | 6 | 125.97 | 10.93 | 17.35 |
|  | Min | Northeast China | 150 | 0 | 6 | 137.67 | 11.92 | 15.60 |
|  | Jiangquhai | Jiangsu Province, China | 10 | 0 | 1 | 17.89 | 10.09 | 3.28 |
|  | Erhualian | Jiangsu Province, China | 5 | 0 | 5 | 81.36 | 8.48 | 13.17 |
|  | Luchuan | Guangxi Zhuang Autonomous Region, China | 200 | 0 | 6 | 135.58 | 11.79 | 14.22 |
|  | Wuzhishan | Hainan Province, China | 230 | 0 | 7 | 153.98 | 11.41 | 20.98 |
|  | Tongcheng | Hubei Province, China | 102 | 0 | 4 | 52.80 | 6.94 | 11.56 |
| **Europe** | Wild boar | Europe | / | 0 | 6 | 85.12 | 7.67 | 7.13 |
|  | Large White | England | / | 0 | 14 | 186.40 | 7.09 | 11.99 |
|  | Duroc | America | / | 0 | 6 | 81.12 | 7.07 | 8.10 |
|  | Landrace | Denmark | / | 0 | 5 | 71.23 | 7.70 | 9.51 |
|  | Pietrain | Belgium | / | 0 | 5 | 63.27 | 6..87 | 8.10 |
|  | Berkshire | England | / | 0 | 3 | 25.21 | 4.40 | 4.77 |
|  | Mangalitsa | Romania | / | 0 | 3 | 75.14 | 13.09 | 6.92 |
|  | Hampshire | America | / | 0 | 2 | 28.66 | 7.84 | 4.43 |
| **Outgroup** | *Sus scrofa* | Sumatera, Indonesia | / | 0 | 2 | 36.83 | 10.20 | 4.28 |
|  | *Sus cebifrons* | Philippines | / | 0 | 1 | 16.38 | 9.35 | 2.94 |
|  | *Sus celebensis* | Sulawesi, Indonesia | / | 0 | 1 | 13.05 | 22.29 | 0.66 |
|  | *Sus verrucosus* | Indonesia | / | 0 | 1 | 20.91 | 11.90 | 1.22 |
|  | *Sus barbatus* | Indonesia and Malaysia | / | 0 | 1 | 10.87 | 6.44 | 1.67 |
|  |  | Total | / | 48 | 185 | 3173.22 |  |  |

Note: The sequencing depth was counted basing on aligned reads.

**Supplementary Table s2:** Information for samples in this study.

| **Serial Number** | **Sample ID** | **Breed** | **SRA ID** | **Sequencing Depth (X)** |
| --- | --- | --- | --- | --- |
| 1 | 44 | European wild boar | ERR173213 | 9.0 |
| 2 | 45 | European wild boar | ERR173214 | 9.3 |
| 3 | 46 | European wild boar | ERR173215 | 5.7 |
| 4 | 47 | European wild boar | ERR173216 | 7.6 |
| 5 | 48 | European wild boar | ERR173217 | 9.4 |
| 6 | 49 | European wild boar | ERR173218 | 7.9 |
| 7 | 16 | Large White | ERR173185 | 5.9 |
| 8 | 17 | Large White | ERR173186 | 9.4 |
| 9 | 18 | Large White | ERR173187 | 9.4 |
| 10 | 19 | Large White | ERR173188 | 9.5 |
| 11 | 20 | Large White | ERR173189 | 8.9 |
| 12 | 21 | Large White | ERR173190 | 8.3 |
| 13 | 22 | Large White | ERR173191 | 9.1 |
| 14 | 23 | Large White | ERR173192 | 6.0 |
| 15 | 24 | Large White | ERR173193 | 9.4 |
| 16 | 25 | Large White | ERR173194 | 7.6 |
| 17 | 26 | Large White | ERR173195 | 7.7 |
| 18 | 27 | Large White | ERR173196 | 8.3 |
| 19 | 28 | Large White | ERR173197 | 7.8 |
| 20 | 29 | Large White | ERR173198 | 7.8 |
| 21 | 1 | Duroc | ERR173170 | 7.7 |
| 22 | 2 | Duroc | ERR173171 | 8.7 |
| 23 | 3 | Duroc | ERR173172 | 5.8 |
| 24 | 4 | Duroc | ERR173173 | 7.2 |
| 25 | 179 | Duroc | SRR030082 | 4.6 |
| 26 | 184 | Duroc | SRR1177806 | 11.9 |
| 27 | 11 | Landrace | ERR173180 | 9.0 |
| 28 | 12 | Landrace | ERR173181 | 10.1 |
| 29 | 13 | Landrace | ERR173182 | 7.0 |
| 30 | 14 | Landrace | ERR173183 | 7.1 |
| 31 | 15 | Landrace | ERR173184 | 7.4 |
| 32 | 35 | Pietrain | ERR173204 | 6.2 |
| 33 | 36 | Pietrain | ERR173205 | 10.3 |
| 34 | 37 | Pietrain | ERR173206 | 8.5 |
| 35 | 38 | Pietrain | ERR173207 | 6.0 |
| 36 | 39 | Pietrain | ERR173208 | 6.2 |
| 37 | 181 | Berkshire | SRR1004277 | 5.2 |
| 38 | 182 | Berkshire | SRR1004278 | 4.5 |
| 39 | 183 | Berkshire | SRR1004279 | 4.4 |
| 40 | 185 | Mangalitsa | SRR1178916 | 14.6 |
| 41 | 186 | Mangalitsa | SRR1178924 | 11.3 |
| 42 | 187 | Mangalitsa | SRR1178925 | 15.2 |
| 43 | 5 | Hampshire | ERR173174 | 8.6 |
| 44 | 6 | Hampshire | ERR173175 | 8.0 |
| 45 | 50 | Chinese wild boar | ERR173219 | 5.6 |
| 46 | 51 | Chinese wild boar | ERR173220 | 9.7 |
| 47 | 52 | Chinese wild boar | ERR173221 | 5.5 |
| 48 | 53 | Chinese wild boar | ERR173222 | 9.4 |
| 49 | 101 | Chinese wild boar | SRR652377 | 4.5 |
| 50 | 102 | Chinese wild boar | SRR652378 | 6.2 |
| 51 | 103 | Chinese wild boar | SRR652379 | 6.1 |
| 52 | 113 | Chinese wild boar | SRR949642 | 11.6 |
| 53 | 114 | Chinese wild boar | SRR949644 | 13.5 |
| 54 | 115 | Chinese wild boar | SRR949646 | 8.5 |
| 55 | 116 | Chinese wild boar | SRR949648 | 8.8 |
| 56 | 117 | Chinese wild boar | SRR949653 | 9.2 |
| 57 | 118 | Chinese wild boar | SRR949654 | 9.4 |
| 58 | 601 | Chinese wild boar | New sequenced | 2.3 |
| 59 | 602 | Chinese wild boar | New sequenced | 3.3 |
| 60 | 603 | Chinese wild boar | New sequenced | 3.1 |
| 61 | 604 | Chinese wild boar | New sequenced | 1.9 |
| 62 | 605 | Chinese wild boar | New sequenced | 3.1 |
| 63 | 606 | Chinese wild boar | New sequenced | 4.4 |
| 64 | 607 | Chinese wild boar | New sequenced | 3.1 |
| 65 | 608 | Chinese wild boar | New sequenced | 4.1 |
| 66 | 609 | Chinese wild boar | New sequenced | 1.8 |
| 67 | 56 | Tibetan | SRR652257 | 4.6 |
| 68 | 57 | Tibetan | SRR652258 | 4.7 |
| 69 | 58 | Tibetan | SRR652259 | 4.2 |
| 70 | 59 | Tibetan | SRR652260 | 5.5 |
| 71 | 60 | Tibetan | SRR652261 | 5.4 |
| 72 | 61 | Tibetan | SRR652262 | 6.0 |
| 73 | 62 | Tibetan | SRR652263 | 4.6 |
| 74 | 63 | Tibetan | SRR652264 | 4.5 |
| 75 | 64 | Tibetan | SRR652265 | 6.7 |
| 76 | 65 | Tibetan | SRR652266 | 4.5 |
| 77 | 66 | Tibetan | SRR652267 | 3.6 |
| 78 | 67 | Tibetan | SRR652268 | 7.1 |
| 79 | 68 | Tibetan | SRR652269 | 5.1 |
| 80 | 69 | Tibetan | SRR652270 | 4.6 |
| 81 | 70 | Tibetan | SRR652302 | 6.8 |
| 82 | 71 | Tibetan | SRR652303 | 5.5 |
| 83 | 72 | Tibetan | SRR652304 | 4.3 |
| 84 | 73 | Tibetan | SRR652305 | 5.5 |
| 85 | 74 | Tibetan | SRR652306 | 4.8 |
| 86 | 75 | Tibetan | SRR652307 | 5.7 |
| 87 | 76 | Tibetan | SRR652327 | 5.9 |
| 88 | 78 | Tibetan | SRR652340 | 5.0 |
| 89 | 79 | Tibetan | SRR652341 | 2.7 |
| 90 | 80 | Tibetan | SRR652342 | 4.4 |
| 91 | 81 | Tibetan | SRR652343 | 4.5 |
| 92 | 82 | Tibetan | SRR652344 | 7.1 |
| 93 | 83 | Tibetan | SRR652345 | 5.5 |
| 94 | 84 | Tibetan | SRR652346 | 6.5 |
| 95 | 85 | Tibetan | SRR652347 | 5.6 |
| 96 | 104 | Tibetan | SRR652443 | 8.6 |
| 97 | 125 | Tibetan | SRR949670 | 12.9 |
| 98 | 126 | Tibetan | SRR949672 | 12.0 |
| 99 | 127 | Tibetan | SRR949674 | 2.9 |
| 100 | 128 | Tibetan | SRR949678 | 10.5 |
| 101 | 129 | Tibetan | SRR949680 | 10.9 |
| 102 | 130 | Tibetan | SRR949682 | 13.2 |
| 103 | 131 | Tibetan | SRR949684 | 13.8 |
| 104 | 132 | Tibetan | SRR949686 | 10.1 |
| 105 | 133 | Tibetan | SRR949688 | 12.8 |
| 106 | 134 | Tibetan | SRR949690 | 13.6 |
| 107 | 135 | Tibetan | SRR949692 | 12.3 |
| 108 | 136 | Tibetan | SRR949694 | 12.5 |
| 109 | 137 | Tibetan | SRR949696 | 12.5 |
| 110 | 138 | Tibetan | SRR949698 | 13.0 |
| 111 | 139 | Tibetan | SRR949700 | 12.4 |
| 112 | 140 | Tibetan | SRR949702 | 12.9 |
| 113 | 141 | Tibetan | SRR949704 | 13.6 |
| 114 | 142 | Tibetan | SRR949706 | 13.1 |
| 115 | 143 | Tibetan | SRR949708 | 11.9 |
| 116 | 144 | Tibetan | SRR949710 | 11.9 |
| 117 | 145 | Tibetan | SRR949712 | 12.1 |
| 118 | 146 | Tibetan | SRR949714 | 12.7 |
| 119 | 614 | Tibetan | New sequenced | 4.0 |
| 120 | 615 | Tibetan | New sequenced | 3.4 |
| 121 | 616 | Tibetan | New sequenced | 5.5 |
| 122 | 617 | Tibetan | New sequenced | 2.4 |
| 123 | 618 | Tibetan | New sequenced | 3.7 |
| 124 | 619 | Tibetan | New sequenced | 4.4 |
| 125 | 620 | Tibetan | New sequenced | 3.2 |
| 126 | 621 | Tibetan | New sequenced | 3.4 |
| 127 | 622 | Tibetan | New sequenced | 2.5 |
| 128 | 623 | Tibetan | New sequenced | 3.0 |
| 129 | 624 | Tibetan | New sequenced | 2.7 |
| 130 | 54 | Xiang | ERR173223 | 8.1 |
| 131 | 55 | Xiang | ERR173224 | 7.8 |
| 132 | 159 | Xiang | SRR949742 | 12.9 |
| 133 | 160 | Xiang | SRR949744 | 13.2 |
| 134 | 161 | Xiang | SRR949746 | 12.6 |
| 135 | 162 | Xiang | SRR949748 | 12.6 |
| 136 | 163 | Xiang | SRR949750 | 12.9 |
| 137 | 164 | Xiang | SRR949752 | 13.2 |
| 138 | 625 | Xiang | New sequenced | 4.7 |
| 139 | 626 | Xiang | New sequenced | 5.1 |
| 140 | 627 | Xiang | New sequenced | 6.1 |
| 141 | 628 | Xiang | New sequenced | 5.9 |
| 142 | 629 | Xiang | New sequenced | 5.8 |
| 143 | 630 | Xiang | New sequenced | 2.4 |
| 144 | 631 | Xiang | New sequenced | 2.4 |
| 145 | 632 | Xiang | New sequenced | 2.4 |
| 146 | 633 | Xiang | New sequenced | 2.1 |
| 147 | 188 | Rongchang | SRR1056426 | 6.7 |
| 148 | 189 | Rongchang | SRR1056427 | 8.3 |
| 149 | 190 | Rongchang | SRR1056429 | 8.3 |
| 150 | 191 | Rongchang | SRR1056430 | 8.0 |
| 151 | 192 | Rongchang | SRR1056431 | 8.0 |
| 152 | 731 | Rongchang | New sequenced | 3.2 |
| 153 | 95 | Neijiang | SRR652357 | 5.0 |
| 154 | 96 | Neijiang | SRR652362 | 5.9 |
| 155 | 97 | Neijiang | SRR652363 | 4.3 |
| 156 | 92 | Ya'nan | SRR652354 | 4.7 |
| 157 | 93 | Ya'nan | SRR652355 | 4.4 |
| 158 | 94 | Ya'nan | SRR652356 | 5.2 |
| 159 | 86 | Penzhou | SRR652348 | 4.5 |
| 160 | 87 | Penzhou | SRR652349 | 4.7 |
| 161 | 88 | Penzhou | SRR652350 | 5.4 |
| 162 | 147 | Hetao | SRR949716 | 11.9 |
| 163 | 148 | Hetao | SRR949718 | 11.9 |
| 164 | 149 | Hetao | SRR949722 | 9.1 |
| 165 | 150 | Hetao | SRR949724 | 11.8 |
| 166 | 151 | Hetao | SRR949726 | 12.4 |
| 167 | 152 | Hetao | SRR949728 | 11.7 |
| 168 | 153 | Min | SRR949730 | 12.7 |
| 169 | 154 | Min | SRR949732 | 12.7 |
| 170 | 155 | Min | SRR949734 | 12.0 |
| 171 | 156 | Min | SRR949736 | 11.3 |
| 172 | 157 | Min | SRR949738 | 13.3 |
| 173 | 158 | Min | SRR949740 | 13.0 |
| 174 | 165 | Laiwu | SRR949754 | 11.8 |
| 175 | 166 | Laiwu | SRR949756 | 12.8 |
| 176 | 167 | Laiwu | SRR949758 | 13.2 |
| 177 | 168 | Laiwu | SRR949760 | 13.0 |
| 178 | 169 | Laiwu | SRR949762 | 12.7 |
| 179 | 170 | Laiwu | SRR949764 | 12.4 |
| 180 | 634 | Laiwu | New sequenced | 4.9 |
| 181 | 635 | Laiwu | New sequenced | 5.8 |
| 182 | 636 | Laiwu | New sequenced | 5.3 |
| 183 | 637 | Laiwu | New sequenced | 4.1 |
| 184 | 638 | Laiwu | New sequenced | 4.3 |
| 185 | 639 | Laiwu | New sequenced | 2.9 |
| 186 | 640 | Laiwu | New sequenced | 2.0 |
| 187 | 641 | Laiwu | New sequenced | 2.5 |
| 188 | 642 | Laiwu | New sequenced | 2.4 |
| 189 | 643 | Laiwu | New sequenced | 2.2 |
| 190 | 725 | Wei | New sequenced | 4.9 |
| 191 | 722 | Wanzhe | New sequenced | 2.6 |
| 192 | 98 | Jinhua | SRR652374 | 4.6 |
| 193 | 99 | Jinhua | SRR652375 | 4.9 |
| 194 | 100 | Jinhua | SRR652376 | 4.2 |
| 195 | 726 | Jinhua | New sequenced | 3.7 |
| 196 | 10 | Jiangquhai | ERR173179 | 10.0 |
| 197 | 30 | Meishan | ERR173199 | 8.6 |
| 198 | 31 | Meishan | ERR173200 | 8.5 |
| 199 | 32 | Meishan | ERR173201 | 8.1 |
| 200 | 33 | Meishan | ERR173202 | 9.7 |
| 201 | 111 | Erhualian | SRR949638 | 13.1 |
| 202 | 112 | Erhualian | SRR949640 | 12.5 |
| 203 | 171 | Erhualian | SRR949765 | 9.9 |
| 204 | 172 | Erhualian | SRR949771 | 9.8 |
| 205 | 173 | Erhualian | SRR949779 | 7.5 |
| 206 | 724 | Jiaxing | New sequenced | 3.5 |
| 207 | 119 | Luchuan | SRR949658 | 9.1 |
| 208 | 120 | Luchuan | SRR949660 | 13.0 |
| 209 | 121 | Luchuan | SRR949662 | 12.8 |
| 210 | 122 | Luchuan | SRR949664 | 12.5 |
| 211 | 123 | Luchuan | SRR949666 | 13.4 |
| 212 | 124 | Luchuan | SRR949668 | 12.9 |
| 213 | 728 | Large  Black-white | New sequenced | 4.0 |
| 214 | 727 | Lantang | New sequenced | 4.2 |
| 215 | 105 | Wuzhishan | SRR949626 | 12.4 |
| 216 | 106 | Wuzhishan | SRR949628 | 12.5 |
| 217 | 107 | Wuzhishan | SRR949630 | 12.5 |
| 218 | 108 | Wuzhishan | SRR949632 | 12.0 |
| 219 | 109 | Wuzhishan | SRR949634 | 12.2 |
| 220 | 110 | Wuzhishan | SRR949636 | 12.6 |
| 221 | 174 | Wuzhishan | SRR448588 | 9.6 |
| 222 | 175 | Tongcheng | SRR1172563 | 7.8 |
| 223 | 176 | Tongcheng | SRR1172577 | 8.6 |
| 224 | 177 | Tongcheng | SRR1216635 | 6.4 |
| 225 | 178 | Tongcheng | SRR1216636 | 6.2 |
| 226 | 730 | Hang | New sequenced | 3.6 |
| 227 | 721 | Putian | New sequenced | 2.4 |
| 228 | 7 | Wild boar of Sumatra | ERR173176 | 10.3 |
| 229 | 9 | Wild boar of Sumatra | ERR173178 | 10.4 |
| 230 | 8 | *Sus barbatus* | ERR173177 | 6.6 |
| 231 | 42 | *Sus verrucosus* | ERR173211 | 11.8 |
| 232 | 41 | *Sus celebensis* | ERR173210 | 22.2 |
| 233 | 40 | *Sus cebifrons* | ERR173209 | 9.4 |

Note: The sequencing depth was counted basing on SNPs within all samples.

**Supplementary Table s3:** Average genetic components of all the 233 samples.

| **Serial Number** | **Sample ID** | **Breed** | **Iteration 1 (K=3)** | | | **Iteration 2 (K=3)** | | | **Iteration 3 (K=3)** | | | **Average (K=3)** | | |
| --- | --- | --- | --- | --- | --- | --- | --- | --- | --- | --- | --- | --- | --- | --- |
|  |  |  | **Europe** | **Asia** | **Outgroup** | **Europe** | **Asia** | **Outgroup** | **Europe** | **Asia** | **Outgroup** | **Europe** | **Asia** | **Outgroup** |
| 1 | 44 | European  wild boar | 1.000 | 0.000 | 0.000 | 0.999 | 0.001 | 0.000 | 1.000 | 0.000 | 0.000 | 1.000 | 0.000 | 0.000 |
| 2 | 45 | European  wild boar | 0.999 | 0.001 | 0.000 | 0.999 | 0.001 | 0.000 | 0.999 | 0.001 | 0.000 | 0.999 | 0.001 | 0.000 |
| 3 | 46 | European  wild boar | 1.000 | 0.000 | 0.000 | 0.999 | 0.001 | 0.000 | 0.999 | 0.001 | 0.000 | 0.999 | 0.001 | 0.000 |
| 4 | 47 | European  wild boar | 1.000 | 0.000 | 0.000 | 0.999 | 0.001 | 0.000 | 0.999 | 0.001 | 0.000 | 0.999 | 0.001 | 0.000 |
| 5 | 48 | European  wild boar | 0.997 | 0.002 | 0.000 | 0.995 | 0.005 | 0.000 | 0.997 | 0.003 | 0.000 | 0.996 | 0.004 | 0.000 |
| 6 | 49 | European  wild boar | 0.999 | 0.001 | 0.000 | 0.998 | 0.002 | 0.000 | 0.999 | 0.001 | 0.000 | 0.999 | 0.001 | 0.000 |
| 7 | 16 | Large White | 0.974 | 0.026 | 0.000 | 0.946 | 0.054 | 0.000 | 0.968 | 0.032 | 0.000 | 0.963 | 0.037 | 0.000 |
| 8 | 17 | Large White | 0.997 | 0.003 | 0.000 | 0.997 | 0.003 | 0.000 | 0.998 | 0.002 | 0.000 | 0.997 | 0.003 | 0.000 |
| 9 | 18 | Large White | 0.999 | 0.001 | 0.000 | 0.999 | 0.001 | 0.000 | 0.998 | 0.001 | 0.000 | 0.999 | 0.001 | 0.000 |
| 10 | 19 | Large White | 0.997 | 0.003 | 0.000 | 0.991 | 0.009 | 0.000 | 0.996 | 0.004 | 0.000 | 0.995 | 0.005 | 0.000 |
| 11 | 20 | Large White | 0.995 | 0.005 | 0.000 | 0.994 | 0.006 | 0.000 | 0.995 | 0.005 | 0.000 | 0.995 | 0.005 | 0.000 |
| 12 | 21 | Large White | 0.988 | 0.012 | 0.000 | 0.983 | 0.017 | 0.000 | 0.988 | 0.012 | 0.000 | 0.986 | 0.014 | 0.000 |
| 13 | 22 | Large White | 0.996 | 0.004 | 0.000 | 0.994 | 0.006 | 0.000 | 0.997 | 0.003 | 0.000 | 0.996 | 0.004 | 0.000 |
| 14 | 23 | Large White | 0.998 | 0.002 | 0.000 | 0.998 | 0.002 | 0.000 | 0.997 | 0.003 | 0.000 | 0.998 | 0.002 | 0.000 |
| 15 | 24 | Large White | 0.996 | 0.004 | 0.000 | 0.989 | 0.011 | 0.000 | 0.994 | 0.006 | 0.000 | 0.993 | 0.007 | 0.000 |
| 16 | 25 | Large White | 0.999 | 0.001 | 0.000 | 0.998 | 0.002 | 0.000 | 0.998 | 0.002 | 0.000 | 0.998 | 0.002 | 0.000 |
| 17 | 26 | Large White | 0.999 | 0.001 | 0.000 | 0.998 | 0.002 | 0.000 | 0.999 | 0.001 | 0.000 | 0.999 | 0.001 | 0.000 |
| 18 | 27 | Large White | 0.998 | 0.002 | 0.000 | 0.998 | 0.002 | 0.000 | 0.999 | 0.001 | 0.000 | 0.998 | 0.002 | 0.000 |
| 19 | 28 | Large White | 0.984 | 0.016 | 0.000 | 0.980 | 0.020 | 0.000 | 0.986 | 0.014 | 0.000 | 0.983 | 0.017 | 0.000 |
| 20 | 29 | Large White | 0.927 | 0.073 | 0.000 | 0.906 | 0.094 | 0.000 | 0.927 | 0.073 | 0.000 | 0.920 | 0.080 | 0.000 |
| 21 | 1 | Duroc | 0.997 | 0.003 | 0.000 | 0.997 | 0.003 | 0.000 | 0.998 | 0.002 | 0.000 | 0.997 | 0.003 | 0.000 |
| 22 | 2 | Duroc | 0.937 | 0.063 | 0.000 | 0.940 | 0.060 | 0.000 | 0.946 | 0.054 | 0.000 | 0.941 | 0.059 | 0.000 |
| 23 | 3 | Duroc | 0.996 | 0.004 | 0.000 | 0.997 | 0.003 | 0.000 | 0.998 | 0.002 | 0.000 | 0.997 | 0.003 | 0.000 |
| 24 | 4 | Duroc | 0.988 | 0.012 | 0.000 | 0.987 | 0.013 | 0.000 | 0.987 | 0.013 | 0.000 | 0.987 | 0.013 | 0.000 |
| 25 | 179 | Duroc | 0.962 | 0.038 | 0.000 | 0.948 | 0.052 | 0.000 | 0.958 | 0.042 | 0.000 | 0.956 | 0.044 | 0.000 |
| 26 | 184 | Duroc | 0.994 | 0.006 | 0.000 | 0.993 | 0.007 | 0.000 | 0.995 | 0.005 | 0.000 | 0.994 | 0.006 | 0.000 |
| 27 | 11 | Landrace | 0.926 | 0.074 | 0.000 | 0.920 | 0.080 | 0.000 | 0.928 | 0.072 | 0.000 | 0.925 | 0.075 | 0.000 |
| 28 | 12 | Landrace | 0.989 | 0.011 | 0.000 | 0.979 | 0.021 | 0.000 | 0.989 | 0.011 | 0.000 | 0.986 | 0.014 | 0.000 |
| 29 | 13 | Landrace | 0.997 | 0.003 | 0.000 | 0.996 | 0.004 | 0.000 | 0.997 | 0.003 | 0.000 | 0.997 | 0.003 | 0.000 |
| 30 | 14 | Landrace | 0.989 | 0.010 | 0.000 | 0.983 | 0.017 | 0.000 | 0.990 | 0.010 | 0.000 | 0.987 | 0.013 | 0.000 |
| 31 | 15 | Landrace | 0.958 | 0.042 | 0.000 | 0.949 | 0.051 | 0.000 | 0.970 | 0.030 | 0.000 | 0.959 | 0.041 | 0.000 |
| 32 | 35 | Pietrain | 0.990 | 0.010 | 0.000 | 0.982 | 0.018 | 0.000 | 0.990 | 0.010 | 0.000 | 0.987 | 0.013 | 0.000 |
| 33 | 36 | Pietrain | 0.896 | 0.104 | 0.000 | 0.885 | 0.115 | 0.000 | 0.898 | 0.102 | 0.000 | 0.893 | 0.107 | 0.000 |
| 34 | 37 | Pietrain | 0.998 | 0.002 | 0.000 | 0.998 | 0.002 | 0.000 | 0.999 | 0.001 | 0.000 | 0.998 | 0.002 | 0.000 |
| 35 | 38 | Pietrain | 0.907 | 0.093 | 0.000 | 0.894 | 0.106 | 0.000 | 0.903 | 0.097 | 0.000 | 0.901 | 0.099 | 0.000 |
| 36 | 39 | Pietrain | 0.996 | 0.004 | 0.000 | 0.995 | 0.005 | 0.000 | 0.996 | 0.004 | 0.000 | 0.996 | 0.004 | 0.000 |
| 37 | 181 | Berkshire | 0.978 | 0.021 | 0.000 | 0.972 | 0.028 | 0.000 | 0.983 | 0.017 | 0.000 | 0.978 | 0.022 | 0.000 |
| 38 | 182 | Berkshire | 0.998 | 0.002 | 0.000 | 0.994 | 0.006 | 0.000 | 0.997 | 0.003 | 0.000 | 0.996 | 0.004 | 0.000 |
| 39 | 183 | Berkshire | 0.969 | 0.031 | 0.000 | 0.951 | 0.049 | 0.000 | 0.964 | 0.036 | 0.000 | 0.961 | 0.039 | 0.000 |
| 40 | 185 | Mangalitsa | 0.995 | 0.005 | 0.000 | 0.991 | 0.009 | 0.000 | 0.995 | 0.005 | 0.000 | 0.994 | 0.006 | 0.000 |
| 41 | 186 | Mangalitsa | 1.000 | 0.000 | 0.000 | 0.999 | 0.001 | 0.000 | 0.999 | 0.001 | 0.000 | 0.999 | 0.001 | 0.000 |
| 42 | 187 | Mangalitsa | 0.999 | 0.001 | 0.000 | 0.998 | 0.002 | 0.000 | 0.999 | 0.001 | 0.000 | 0.999 | 0.001 | 0.000 |
| 43 | 5 | Hampshire | 0.923 | 0.074 | 0.004 | 0.905 | 0.092 | 0.003 | 0.912 | 0.084 | 0.003 | 0.913 | 0.083 | 0.003 |
| 44 | 6 | Hampshire | 0.896 | 0.103 | 0.001 | 0.870 | 0.129 | 0.001 | 0.890 | 0.109 | 0.001 | 0.885 | 0.114 | 0.001 |
| 45 | 50 | Chinese  wild boar | 0.004 | 0.995 | 0.001 | 0.001 | 0.998 | 0.001 | 0.004 | 0.995 | 0.001 | 0.003 | 0.996 | 0.001 |
| 46 | 51 | Chinese  wild boar | 0.018 | 0.982 | 0.001 | 0.011 | 0.988 | 0.000 | 0.019 | 0.980 | 0.001 | 0.016 | 0.983 | 0.001 |
| 47 | 52 | Chinese  wild boar | 0.008 | 0.991 | 0.000 | 0.005 | 0.995 | 0.000 | 0.011 | 0.988 | 0.000 | 0.008 | 0.992 | 0.000 |
| 48 | 53 | Chinese  wild boar | 0.004 | 0.995 | 0.000 | 0.001 | 0.999 | 0.000 | 0.004 | 0.995 | 0.000 | 0.003 | 0.997 | 0.000 |
| 49 | 101 | Chinese  wild boar | 0.002 | 0.998 | 0.000 | 0.001 | 0.999 | 0.000 | 0.003 | 0.997 | 0.000 | 0.002 | 0.998 | 0.000 |
| 50 | 102 | Chinese  wild boar | 0.004 | 0.996 | 0.000 | 0.002 | 0.998 | 0.000 | 0.007 | 0.993 | 0.000 | 0.004 | 0.996 | 0.000 |
| 51 | 103 | Chinese  wild boar | 0.003 | 0.997 | 0.000 | 0.001 | 0.999 | 0.000 | 0.002 | 0.998 | 0.000 | 0.002 | 0.998 | 0.000 |
| 52 | 113 | Chinese  wild boar | 0.002 | 0.997 | 0.001 | 0.000 | 0.999 | 0.000 | 0.002 | 0.997 | 0.000 | 0.001 | 0.998 | 0.000 |
| 53 | 114 | Chinese  wild boar | 0.002 | 0.997 | 0.000 | 0.001 | 0.999 | 0.000 | 0.002 | 0.997 | 0.000 | 0.002 | 0.998 | 0.000 |
| 54 | 115 | Chinese  wild boar | 0.037 | 0.963 | 0.000 | 0.010 | 0.990 | 0.000 | 0.036 | 0.964 | 0.000 | 0.028 | 0.972 | 0.000 |
| 55 | 116 | Chinese  wild boar | 0.002 | 0.997 | 0.000 | 0.001 | 0.999 | 0.000 | 0.002 | 0.997 | 0.000 | 0.002 | 0.998 | 0.000 |
| 56 | 117 | Chinese  wild boar | 0.004 | 0.994 | 0.002 | 0.001 | 0.997 | 0.001 | 0.003 | 0.995 | 0.002 | 0.003 | 0.996 | 0.002 |
| 57 | 118 | Chinese  wild boar | 0.005 | 0.993 | 0.002 | 0.001 | 0.998 | 0.001 | 0.004 | 0.994 | 0.002 | 0.003 | 0.995 | 0.002 |
| 58 | 601 | Chinese  wild boar | 0.832 | 0.168 | 0.000 | 0.761 | 0.238 | 0.000 | 0.849 | 0.151 | 0.000 | 0.814 | 0.186 | 0.000 |
| 59 | 602 | Chinese  wild boar | 0.038 | 0.958 | 0.004 | 0.005 | 0.992 | 0.004 | 0.037 | 0.959 | 0.004 | 0.027 | 0.969 | 0.004 |
| 60 | 603 | Chinese  wild boar | 0.005 | 0.995 | 0.000 | 0.002 | 0.998 | 0.000 | 0.005 | 0.995 | 0.000 | 0.004 | 0.996 | 0.000 |
| 61 | 604 | Chinese  wild boar | 0.003 | 0.997 | 0.000 | 0.001 | 0.999 | 0.000 | 0.003 | 0.997 | 0.000 | 0.002 | 0.998 | 0.000 |
| 62 | 605 | Chinese  wild boar | 0.006 | 0.994 | 0.000 | 0.003 | 0.997 | 0.000 | 0.006 | 0.994 | 0.000 | 0.005 | 0.995 | 0.000 |
| 63 | 606 | Chinese  wild boar | 0.003 | 0.997 | 0.000 | 0.001 | 0.999 | 0.000 | 0.002 | 0.998 | 0.000 | 0.002 | 0.998 | 0.000 |
| 64 | 607 | Chinese  wild boar | 0.003 | 0.997 | 0.000 | 0.001 | 0.999 | 0.000 | 0.003 | 0.997 | 0.000 | 0.002 | 0.998 | 0.000 |
| 65 | 608 | Chinese  wild boar | 0.003 | 0.997 | 0.000 | 0.002 | 0.998 | 0.000 | 0.005 | 0.995 | 0.000 | 0.003 | 0.997 | 0.000 |
| 66 | 609 | Chinese  wild boar | 0.990 | 0.010 | 0.000 | 0.991 | 0.009 | 0.000 | 0.989 | 0.011 | 0.000 | 0.990 | 0.010 | 0.000 |
| 67 | 56 | Tibetan | 0.025 | 0.975 | 0.000 | 0.004 | 0.996 | 0.000 | 0.025 | 0.975 | 0.000 | 0.018 | 0.982 | 0.000 |
| 68 | 57 | Tibetan | 0.002 | 0.998 | 0.000 | 0.001 | 0.999 | 0.000 | 0.003 | 0.997 | 0.000 | 0.002 | 0.998 | 0.000 |
| 69 | 58 | Tibetan | 0.005 | 0.995 | 0.000 | 0.002 | 0.998 | 0.000 | 0.006 | 0.994 | 0.000 | 0.004 | 0.996 | 0.000 |
| 70 | 59 | Tibetan | 0.003 | 0.997 | 0.000 | 0.001 | 0.999 | 0.000 | 0.002 | 0.998 | 0.000 | 0.002 | 0.998 | 0.000 |
| 71 | 60 | Tibetan | 0.005 | 0.995 | 0.000 | 0.002 | 0.998 | 0.000 | 0.007 | 0.993 | 0.000 | 0.005 | 0.995 | 0.000 |
| 72 | 61 | Tibetan | 0.120 | 0.880 | 0.000 | 0.111 | 0.889 | 0.000 | 0.124 | 0.876 | 0.000 | 0.118 | 0.882 | 0.000 |
| 73 | 62 | Tibetan | 0.282 | 0.718 | 0.000 | 0.282 | 0.718 | 0.000 | 0.283 | 0.717 | 0.000 | 0.282 | 0.718 | 0.000 |
| 74 | 63 | Tibetan | 0.166 | 0.834 | 0.000 | 0.162 | 0.838 | 0.000 | 0.182 | 0.818 | 0.000 | 0.170 | 0.830 | 0.000 |
| 75 | 64 | Tibetan | 0.128 | 0.872 | 0.000 | 0.102 | 0.898 | 0.000 | 0.125 | 0.875 | 0.000 | 0.118 | 0.882 | 0.000 |
| 76 | 65 | Tibetan | 0.003 | 0.997 | 0.000 | 0.001 | 0.999 | 0.000 | 0.004 | 0.996 | 0.000 | 0.003 | 0.997 | 0.000 |
| 77 | 66 | Tibetan | 0.050 | 0.950 | 0.000 | 0.019 | 0.981 | 0.000 | 0.060 | 0.940 | 0.000 | 0.043 | 0.957 | 0.000 |
| 78 | 67 | Tibetan | 0.108 | 0.892 | 0.000 | 0.098 | 0.902 | 0.000 | 0.112 | 0.888 | 0.000 | 0.106 | 0.894 | 0.000 |
| 79 | 68 | Tibetan | 0.168 | 0.832 | 0.000 | 0.164 | 0.836 | 0.000 | 0.184 | 0.816 | 0.000 | 0.172 | 0.828 | 0.000 |
| 80 | 69 | Tibetan | 0.153 | 0.847 | 0.000 | 0.136 | 0.864 | 0.000 | 0.141 | 0.859 | 0.000 | 0.143 | 0.857 | 0.000 |
| 81 | 70 | Tibetan | 0.162 | 0.838 | 0.000 | 0.152 | 0.848 | 0.000 | 0.166 | 0.834 | 0.000 | 0.160 | 0.840 | 0.000 |
| 82 | 71 | Tibetan | 0.447 | 0.553 | 0.000 | 0.413 | 0.587 | 0.000 | 0.436 | 0.564 | 0.000 | 0.432 | 0.568 | 0.000 |
| 83 | 72 | Tibetan | 0.636 | 0.364 | 0.000 | 0.609 | 0.391 | 0.000 | 0.653 | 0.347 | 0.000 | 0.633 | 0.367 | 0.000 |
| 84 | 73 | Tibetan | 0.268 | 0.732 | 0.000 | 0.228 | 0.772 | 0.000 | 0.252 | 0.748 | 0.000 | 0.249 | 0.751 | 0.000 |
| 85 | 74 | Tibetan | 0.266 | 0.734 | 0.000 | 0.247 | 0.753 | 0.000 | 0.259 | 0.741 | 0.000 | 0.257 | 0.743 | 0.000 |
| 86 | 75 | Tibetan | 0.277 | 0.723 | 0.000 | 0.260 | 0.740 | 0.000 | 0.279 | 0.721 | 0.000 | 0.272 | 0.728 | 0.000 |
| 87 | 76 | Tibetan | 0.145 | 0.855 | 0.000 | 0.119 | 0.881 | 0.000 | 0.132 | 0.868 | 0.000 | 0.132 | 0.868 | 0.000 |
| 88 | 78 | Tibetan | 0.148 | 0.852 | 0.000 | 0.135 | 0.865 | 0.000 | 0.139 | 0.861 | 0.000 | 0.141 | 0.859 | 0.000 |
| 89 | 79 | Tibetan | 0.125 | 0.875 | 0.000 | 0.109 | 0.891 | 0.000 | 0.121 | 0.879 | 0.000 | 0.118 | 0.882 | 0.000 |
| 90 | 80 | Tibetan | 0.197 | 0.803 | 0.000 | 0.179 | 0.821 | 0.000 | 0.191 | 0.809 | 0.000 | 0.189 | 0.811 | 0.000 |
| 91 | 81 | Tibetan | 0.118 | 0.882 | 0.000 | 0.082 | 0.918 | 0.000 | 0.110 | 0.890 | 0.000 | 0.103 | 0.897 | 0.000 |
| 92 | 82 | Tibetan | 0.099 | 0.901 | 0.000 | 0.079 | 0.921 | 0.000 | 0.098 | 0.902 | 0.000 | 0.092 | 0.908 | 0.000 |
| 93 | 83 | Tibetan | 0.085 | 0.915 | 0.000 | 0.049 | 0.951 | 0.000 | 0.080 | 0.920 | 0.000 | 0.071 | 0.929 | 0.000 |
| 94 | 84 | Tibetan | 0.035 | 0.965 | 0.000 | 0.027 | 0.973 | 0.000 | 0.036 | 0.964 | 0.000 | 0.033 | 0.967 | 0.000 |
| 95 | 85 | Tibetan | 0.103 | 0.897 | 0.000 | 0.073 | 0.927 | 0.000 | 0.102 | 0.898 | 0.000 | 0.093 | 0.907 | 0.000 |
| 96 | 104 | Tibetan | 0.004 | 0.996 | 0.000 | 0.001 | 0.999 | 0.000 | 0.003 | 0.997 | 0.000 | 0.003 | 0.997 | 0.000 |
| 97 | 125 | Tibetan | 0.069 | 0.931 | 0.000 | 0.048 | 0.952 | 0.000 | 0.068 | 0.932 | 0.000 | 0.062 | 0.938 | 0.000 |
| 98 | 126 | Tibetan | 0.080 | 0.919 | 0.000 | 0.072 | 0.928 | 0.000 | 0.078 | 0.922 | 0.000 | 0.077 | 0.923 | 0.000 |
| 99 | 127 | Tibetan | 0.099 | 0.901 | 0.000 | 0.090 | 0.910 | 0.000 | 0.096 | 0.904 | 0.000 | 0.095 | 0.905 | 0.000 |
| 100 | 128 | Tibetan | 0.148 | 0.852 | 0.000 | 0.138 | 0.862 | 0.000 | 0.143 | 0.857 | 0.000 | 0.143 | 0.857 | 0.000 |
| 101 | 129 | Tibetan | 0.137 | 0.863 | 0.000 | 0.129 | 0.871 | 0.000 | 0.131 | 0.869 | 0.000 | 0.132 | 0.868 | 0.000 |
| 102 | 130 | Tibetan | 0.076 | 0.924 | 0.000 | 0.061 | 0.939 | 0.000 | 0.074 | 0.926 | 0.000 | 0.070 | 0.930 | 0.000 |
| 103 | 131 | Tibetan | 0.040 | 0.960 | 0.000 | 0.026 | 0.974 | 0.000 | 0.045 | 0.955 | 0.000 | 0.037 | 0.963 | 0.000 |
| 104 | 132 | Tibetan | 0.003 | 0.997 | 0.000 | 0.001 | 0.999 | 0.000 | 0.002 | 0.998 | 0.000 | 0.002 | 0.998 | 0.000 |
| 105 | 133 | Tibetan | 0.030 | 0.970 | 0.000 | 0.029 | 0.971 | 0.000 | 0.034 | 0.966 | 0.000 | 0.031 | 0.969 | 0.000 |
| 106 | 134 | Tibetan | 0.079 | 0.921 | 0.000 | 0.064 | 0.936 | 0.000 | 0.080 | 0.920 | 0.000 | 0.074 | 0.926 | 0.000 |
| 107 | 135 | Tibetan | 0.008 | 0.992 | 0.000 | 0.003 | 0.997 | 0.000 | 0.007 | 0.993 | 0.000 | 0.006 | 0.994 | 0.000 |
| 108 | 136 | Tibetan | 0.047 | 0.953 | 0.000 | 0.039 | 0.961 | 0.000 | 0.051 | 0.949 | 0.000 | 0.046 | 0.954 | 0.000 |
| 109 | 137 | Tibetan | 0.002 | 0.998 | 0.000 | 0.001 | 0.999 | 0.000 | 0.002 | 0.998 | 0.000 | 0.002 | 0.998 | 0.000 |
| 110 | 138 | Tibetan | 0.003 | 0.997 | 0.000 | 0.001 | 0.999 | 0.000 | 0.003 | 0.997 | 0.000 | 0.002 | 0.998 | 0.000 |
| 111 | 139 | Tibetan | 0.013 | 0.987 | 0.000 | 0.004 | 0.996 | 0.000 | 0.014 | 0.986 | 0.000 | 0.010 | 0.990 | 0.000 |
| 112 | 140 | Tibetan | 0.002 | 0.998 | 0.000 | 0.001 | 0.999 | 0.000 | 0.003 | 0.997 | 0.000 | 0.002 | 0.998 | 0.000 |
| 113 | 141 | Tibetan | 0.002 | 0.997 | 0.000 | 0.001 | 0.999 | 0.000 | 0.003 | 0.997 | 0.000 | 0.002 | 0.998 | 0.000 |
| 114 | 142 | Tibetan | 0.034 | 0.965 | 0.000 | 0.011 | 0.989 | 0.000 | 0.031 | 0.969 | 0.000 | 0.025 | 0.975 | 0.000 |
| 115 | 143 | Tibetan | 0.020 | 0.980 | 0.000 | 0.004 | 0.996 | 0.000 | 0.018 | 0.982 | 0.000 | 0.014 | 0.986 | 0.000 |
| 116 | 144 | Tibetan | 0.075 | 0.925 | 0.000 | 0.060 | 0.940 | 0.000 | 0.073 | 0.927 | 0.000 | 0.069 | 0.931 | 0.000 |
| 117 | 145 | Tibetan | 0.147 | 0.853 | 0.000 | 0.137 | 0.863 | 0.000 | 0.146 | 0.854 | 0.000 | 0.143 | 0.857 | 0.000 |
| 118 | 146 | Tibetan | 0.084 | 0.916 | 0.000 | 0.079 | 0.921 | 0.000 | 0.084 | 0.916 | 0.000 | 0.082 | 0.918 | 0.000 |
| 119 | 614 | Tibetan | 0.150 | 0.850 | 0.000 | 0.118 | 0.882 | 0.000 | 0.147 | 0.853 | 0.000 | 0.138 | 0.862 | 0.000 |
| 120 | 615 | Tibetan | 0.105 | 0.895 | 0.000 | 0.092 | 0.908 | 0.000 | 0.115 | 0.885 | 0.000 | 0.104 | 0.896 | 0.000 |
| 121 | 616 | Tibetan | 0.114 | 0.886 | 0.000 | 0.097 | 0.903 | 0.000 | 0.116 | 0.884 | 0.000 | 0.109 | 0.891 | 0.000 |
| 122 | 617 | Tibetan | 0.036 | 0.964 | 0.000 | 0.026 | 0.974 | 0.000 | 0.044 | 0.956 | 0.000 | 0.035 | 0.965 | 0.000 |
| 123 | 618 | Tibetan | 0.032 | 0.968 | 0.000 | 0.021 | 0.979 | 0.000 | 0.035 | 0.965 | 0.000 | 0.029 | 0.971 | 0.000 |
| 124 | 619 | Tibetan | 0.141 | 0.859 | 0.000 | 0.124 | 0.876 | 0.000 | 0.128 | 0.872 | 0.000 | 0.131 | 0.869 | 0.000 |
| 125 | 620 | Tibetan | 0.049 | 0.951 | 0.000 | 0.028 | 0.972 | 0.000 | 0.047 | 0.953 | 0.000 | 0.041 | 0.959 | 0.000 |
| 126 | 621 | Tibetan | 0.007 | 0.993 | 0.000 | 0.003 | 0.997 | 0.000 | 0.006 | 0.994 | 0.000 | 0.005 | 0.995 | 0.000 |
| 127 | 622 | Tibetan | 0.006 | 0.994 | 0.000 | 0.003 | 0.997 | 0.000 | 0.006 | 0.994 | 0.000 | 0.005 | 0.995 | 0.000 |
| 128 | 623 | Tibetan | 0.294 | 0.706 | 0.000 | 0.291 | 0.709 | 0.000 | 0.299 | 0.701 | 0.000 | 0.295 | 0.705 | 0.000 |
| 129 | 624 | Tibetan | 0.378 | 0.622 | 0.000 | 0.313 | 0.687 | 0.000 | 0.360 | 0.640 | 0.000 | 0.350 | 0.650 | 0.000 |
| 130 | 54 | Xiang | 0.045 | 0.953 | 0.002 | 0.012 | 0.986 | 0.002 | 0.038 | 0.960 | 0.002 | 0.032 | 0.966 | 0.002 |
| 131 | 55 | Xiang | 0.014 | 0.984 | 0.002 | 0.003 | 0.995 | 0.002 | 0.012 | 0.986 | 0.002 | 0.010 | 0.988 | 0.002 |
| 132 | 159 | Xiang | 0.006 | 0.994 | 0.000 | 0.001 | 0.999 | 0.000 | 0.004 | 0.996 | 0.000 | 0.004 | 0.996 | 0.000 |
| 133 | 160 | Xiang | 0.016 | 0.984 | 0.000 | 0.006 | 0.994 | 0.000 | 0.014 | 0.986 | 0.000 | 0.012 | 0.988 | 0.000 |
| 134 | 161 | Xiang | 0.002 | 0.998 | 0.000 | 0.001 | 0.999 | 0.000 | 0.002 | 0.998 | 0.000 | 0.002 | 0.998 | 0.000 |
| 135 | 162 | Xiang | 0.021 | 0.979 | 0.000 | 0.004 | 0.996 | 0.000 | 0.019 | 0.981 | 0.000 | 0.015 | 0.985 | 0.000 |
| 136 | 163 | Xiang | 0.002 | 0.998 | 0.000 | 0.001 | 0.999 | 0.000 | 0.003 | 0.997 | 0.000 | 0.002 | 0.998 | 0.000 |
| 137 | 164 | Xiang | 0.002 | 0.998 | 0.000 | 0.001 | 0.999 | 0.000 | 0.002 | 0.998 | 0.000 | 0.002 | 0.998 | 0.000 |
| 138 | 625 | Xiang | 0.003 | 0.997 | 0.000 | 0.001 | 0.999 | 0.000 | 0.003 | 0.997 | 0.000 | 0.002 | 0.998 | 0.000 |
| 139 | 626 | Xiang | 0.002 | 0.998 | 0.000 | 0.001 | 0.999 | 0.000 | 0.002 | 0.998 | 0.000 | 0.002 | 0.998 | 0.000 |
| 140 | 627 | Xiang | 0.002 | 0.998 | 0.000 | 0.001 | 0.999 | 0.000 | 0.002 | 0.998 | 0.000 | 0.002 | 0.998 | 0.000 |
| 141 | 628 | Xiang | 0.002 | 0.998 | 0.000 | 0.001 | 0.999 | 0.000 | 0.003 | 0.997 | 0.000 | 0.002 | 0.998 | 0.000 |
| 142 | 629 | Xiang | 0.002 | 0.998 | 0.000 | 0.000 | 1.000 | 0.000 | 0.002 | 0.998 | 0.000 | 0.001 | 0.999 | 0.000 |
| 143 | 630 | Xiang | 0.035 | 0.965 | 0.000 | 0.025 | 0.975 | 0.000 | 0.038 | 0.962 | 0.000 | 0.033 | 0.967 | 0.000 |
| 144 | 631 | Xiang | 0.005 | 0.995 | 0.000 | 0.002 | 0.998 | 0.000 | 0.004 | 0.996 | 0.000 | 0.004 | 0.996 | 0.000 |
| 145 | 632 | Xiang | 0.006 | 0.994 | 0.000 | 0.003 | 0.997 | 0.000 | 0.006 | 0.994 | 0.000 | 0.005 | 0.995 | 0.000 |
| 146 | 633 | Xiang | 0.060 | 0.940 | 0.000 | 0.050 | 0.950 | 0.000 | 0.068 | 0.932 | 0.000 | 0.059 | 0.941 | 0.000 |
| 147 | 188 | Rongchang | 0.003 | 0.997 | 0.000 | 0.001 | 0.999 | 0.000 | 0.003 | 0.997 | 0.000 | 0.002 | 0.998 | 0.000 |
| 148 | 189 | Rongchang | 0.002 | 0.998 | 0.000 | 0.001 | 0.999 | 0.000 | 0.002 | 0.998 | 0.000 | 0.002 | 0.998 | 0.000 |
| 149 | 190 | Rongchang | 0.002 | 0.998 | 0.000 | 0.001 | 0.999 | 0.000 | 0.002 | 0.998 | 0.000 | 0.002 | 0.998 | 0.000 |
| 150 | 191 | Rongchang | 0.003 | 0.997 | 0.000 | 0.001 | 0.999 | 0.000 | 0.002 | 0.998 | 0.000 | 0.002 | 0.998 | 0.000 |
| 151 | 192 | Rongchang | 0.002 | 0.998 | 0.000 | 0.000 | 1.000 | 0.000 | 0.002 | 0.998 | 0.000 | 0.001 | 0.999 | 0.000 |
| 152 | 731 | Rongchang | 0.003 | 0.997 | 0.000 | 0.001 | 0.999 | 0.000 | 0.003 | 0.997 | 0.000 | 0.002 | 0.998 | 0.000 |
| 153 | 95 | Neijiang | 0.004 | 0.996 | 0.000 | 0.001 | 0.999 | 0.000 | 0.003 | 0.997 | 0.000 | 0.003 | 0.997 | 0.000 |
| 154 | 96 | Neijiang | 0.014 | 0.986 | 0.000 | 0.003 | 0.997 | 0.000 | 0.010 | 0.990 | 0.000 | 0.009 | 0.991 | 0.000 |
| 155 | 97 | Neijiang | 0.021 | 0.979 | 0.000 | 0.003 | 0.997 | 0.000 | 0.017 | 0.983 | 0.000 | 0.014 | 0.986 | 0.000 |
| 156 | 92 | Ya'nan | 0.003 | 0.997 | 0.000 | 0.001 | 0.999 | 0.000 | 0.002 | 0.998 | 0.000 | 0.002 | 0.998 | 0.000 |
| 157 | 93 | Ya'nan | 0.002 | 0.998 | 0.000 | 0.000 | 1.000 | 0.000 | 0.002 | 0.998 | 0.000 | 0.001 | 0.999 | 0.000 |
| 158 | 94 | Ya'nan | 0.002 | 0.998 | 0.000 | 0.002 | 0.998 | 0.000 | 0.003 | 0.997 | 0.000 | 0.002 | 0.998 | 0.000 |
| 159 | 86 | Penzhou | 0.129 | 0.871 | 0.000 | 0.104 | 0.896 | 0.000 | 0.139 | 0.861 | 0.000 | 0.124 | 0.876 | 0.000 |
| 160 | 87 | Penzhou | 0.255 | 0.745 | 0.000 | 0.224 | 0.776 | 0.000 | 0.258 | 0.742 | 0.000 | 0.246 | 0.754 | 0.000 |
| 161 | 88 | Penzhou | 0.563 | 0.437 | 0.000 | 0.560 | 0.440 | 0.000 | 0.559 | 0.441 | 0.000 | 0.561 | 0.439 | 0.000 |
| 162 | 147 | Hetao | 0.105 | 0.895 | 0.000 | 0.098 | 0.902 | 0.000 | 0.116 | 0.884 | 0.000 | 0.106 | 0.894 | 0.000 |
| 163 | 148 | Hetao | 0.089 | 0.911 | 0.000 | 0.075 | 0.925 | 0.000 | 0.085 | 0.915 | 0.000 | 0.083 | 0.917 | 0.000 |
| 164 | 149 | Hetao | 0.099 | 0.901 | 0.000 | 0.069 | 0.931 | 0.000 | 0.094 | 0.906 | 0.000 | 0.087 | 0.913 | 0.000 |
| 165 | 150 | Hetao | 0.104 | 0.896 | 0.000 | 0.089 | 0.911 | 0.000 | 0.112 | 0.888 | 0.000 | 0.102 | 0.898 | 0.000 |
| 166 | 151 | Hetao | 0.097 | 0.903 | 0.000 | 0.080 | 0.920 | 0.000 | 0.097 | 0.903 | 0.000 | 0.091 | 0.909 | 0.000 |
| 167 | 152 | Hetao | 0.171 | 0.829 | 0.000 | 0.147 | 0.853 | 0.000 | 0.167 | 0.833 | 0.000 | 0.162 | 0.838 | 0.000 |
| 168 | 153 | Min | 0.173 | 0.827 | 0.000 | 0.148 | 0.852 | 0.000 | 0.169 | 0.831 | 0.000 | 0.163 | 0.837 | 0.000 |
| 169 | 154 | Min | 0.145 | 0.855 | 0.000 | 0.126 | 0.874 | 0.000 | 0.143 | 0.857 | 0.000 | 0.138 | 0.862 | 0.000 |
| 170 | 155 | Min | 0.357 | 0.643 | 0.000 | 0.332 | 0.668 | 0.000 | 0.361 | 0.639 | 0.000 | 0.350 | 0.650 | 0.000 |
| 171 | 156 | Min | 0.285 | 0.715 | 0.000 | 0.265 | 0.735 | 0.000 | 0.285 | 0.715 | 0.000 | 0.278 | 0.722 | 0.000 |
| 172 | 157 | Min | 0.287 | 0.713 | 0.000 | 0.263 | 0.737 | 0.000 | 0.289 | 0.711 | 0.000 | 0.280 | 0.720 | 0.000 |
| 173 | 158 | Min | 0.041 | 0.959 | 0.000 | 0.026 | 0.974 | 0.000 | 0.037 | 0.963 | 0.000 | 0.035 | 0.965 | 0.000 |
| 174 | 165 | Laiwu | 0.265 | 0.735 | 0.000 | 0.207 | 0.793 | 0.000 | 0.260 | 0.740 | 0.000 | 0.244 | 0.756 | 0.000 |
| 175 | 166 | Laiwu | 0.069 | 0.931 | 0.000 | 0.036 | 0.964 | 0.000 | 0.070 | 0.930 | 0.000 | 0.058 | 0.942 | 0.000 |
| 176 | 167 | Laiwu | 0.185 | 0.815 | 0.000 | 0.146 | 0.854 | 0.000 | 0.183 | 0.817 | 0.000 | 0.171 | 0.829 | 0.000 |
| 177 | 168 | Laiwu | 0.253 | 0.747 | 0.000 | 0.206 | 0.794 | 0.000 | 0.244 | 0.756 | 0.000 | 0.234 | 0.766 | 0.000 |
| 178 | 169 | Laiwu | 0.257 | 0.743 | 0.000 | 0.174 | 0.826 | 0.000 | 0.234 | 0.766 | 0.000 | 0.222 | 0.778 | 0.000 |
| 179 | 170 | Laiwu | 0.300 | 0.700 | 0.000 | 0.231 | 0.769 | 0.000 | 0.297 | 0.703 | 0.000 | 0.276 | 0.724 | 0.000 |
| 180 | 634 | Laiwu | 0.261 | 0.739 | 0.000 | 0.199 | 0.801 | 0.000 | 0.248 | 0.752 | 0.000 | 0.236 | 0.764 | 0.000 |
| 181 | 635 | Laiwu | 0.191 | 0.809 | 0.000 | 0.148 | 0.852 | 0.000 | 0.185 | 0.815 | 0.000 | 0.175 | 0.825 | 0.000 |
| 182 | 636 | Laiwu | 0.253 | 0.747 | 0.000 | 0.152 | 0.848 | 0.000 | 0.261 | 0.739 | 0.000 | 0.222 | 0.778 | 0.000 |
| 183 | 637 | Laiwu | 0.405 | 0.595 | 0.000 | 0.360 | 0.640 | 0.000 | 0.431 | 0.569 | 0.000 | 0.399 | 0.601 | 0.000 |
| 184 | 638 | Laiwu | 0.135 | 0.865 | 0.000 | 0.062 | 0.938 | 0.000 | 0.136 | 0.864 | 0.000 | 0.111 | 0.889 | 0.000 |
| 185 | 639 | Laiwu | 0.087 | 0.913 | 0.000 | 0.086 | 0.914 | 0.000 | 0.081 | 0.919 | 0.000 | 0.085 | 0.915 | 0.000 |
| 186 | 640 | Laiwu | 0.217 | 0.783 | 0.000 | 0.174 | 0.826 | 0.000 | 0.226 | 0.774 | 0.000 | 0.206 | 0.794 | 0.000 |
| 187 | 641 | Laiwu | 0.060 | 0.939 | 0.000 | 0.042 | 0.958 | 0.000 | 0.075 | 0.925 | 0.000 | 0.059 | 0.941 | 0.000 |
| 188 | 642 | Laiwu | 0.178 | 0.822 | 0.000 | 0.162 | 0.838 | 0.000 | 0.191 | 0.809 | 0.000 | 0.177 | 0.823 | 0.000 |
| 189 | 643 | Laiwu | 0.126 | 0.874 | 0.000 | 0.047 | 0.953 | 0.000 | 0.123 | 0.877 | 0.000 | 0.099 | 0.901 | 0.000 |
| 190 | 725 | Wei | 0.179 | 0.820 | 0.002 | 0.159 | 0.840 | 0.001 | 0.191 | 0.807 | 0.001 | 0.176 | 0.822 | 0.001 |
| 191 | 722 | Wanzhe | 0.042 | 0.957 | 0.001 | 0.004 | 0.996 | 0.000 | 0.033 | 0.967 | 0.001 | 0.026 | 0.973 | 0.001 |
| 192 | 98 | Jinhua | 0.005 | 0.995 | 0.000 | 0.002 | 0.998 | 0.000 | 0.003 | 0.997 | 0.000 | 0.003 | 0.997 | 0.000 |
| 193 | 99 | Jinhua | 0.002 | 0.998 | 0.000 | 0.001 | 0.999 | 0.000 | 0.002 | 0.998 | 0.000 | 0.002 | 0.998 | 0.000 |
| 194 | 100 | Jinhua | 0.003 | 0.997 | 0.000 | 0.001 | 0.999 | 0.000 | 0.004 | 0.996 | 0.000 | 0.003 | 0.997 | 0.000 |
| 195 | 726 | Jinhua | 0.428 | 0.572 | 0.000 | 0.404 | 0.596 | 0.000 | 0.423 | 0.577 | 0.000 | 0.418 | 0.582 | 0.000 |
| 196 | 10 | Jiangquhai | 0.042 | 0.957 | 0.001 | 0.031 | 0.968 | 0.001 | 0.045 | 0.954 | 0.001 | 0.039 | 0.960 | 0.001 |
| 197 | 30 | Meishan | 0.041 | 0.959 | 0.000 | 0.032 | 0.968 | 0.000 | 0.047 | 0.953 | 0.000 | 0.040 | 0.960 | 0.000 |
| 198 | 31 | Meishan | 0.111 | 0.889 | 0.000 | 0.104 | 0.896 | 0.000 | 0.106 | 0.894 | 0.000 | 0.107 | 0.893 | 0.000 |
| 199 | 32 | Meishan | 0.008 | 0.992 | 0.000 | 0.003 | 0.997 | 0.000 | 0.012 | 0.988 | 0.000 | 0.008 | 0.992 | 0.000 |
| 200 | 33 | Meishan | 0.012 | 0.988 | 0.000 | 0.003 | 0.997 | 0.000 | 0.017 | 0.983 | 0.000 | 0.011 | 0.989 | 0.000 |
| 201 | 111 | Erhualian | 0.003 | 0.997 | 0.000 | 0.001 | 0.999 | 0.000 | 0.003 | 0.997 | 0.000 | 0.002 | 0.998 | 0.000 |
| 202 | 112 | Erhualian | 0.006 | 0.994 | 0.000 | 0.002 | 0.998 | 0.000 | 0.004 | 0.996 | 0.000 | 0.004 | 0.996 | 0.000 |
| 203 | 171 | Erhualian | 0.064 | 0.936 | 0.000 | 0.057 | 0.943 | 0.000 | 0.068 | 0.932 | 0.000 | 0.063 | 0.937 | 0.000 |
| 204 | 172 | Erhualian | 0.024 | 0.976 | 0.000 | 0.017 | 0.983 | 0.000 | 0.027 | 0.973 | 0.000 | 0.023 | 0.977 | 0.000 |
| 205 | 173 | Erhualian | 0.003 | 0.997 | 0.000 | 0.001 | 0.999 | 0.000 | 0.003 | 0.997 | 0.000 | 0.002 | 0.998 | 0.000 |
| 206 | 724 | Jiaxing | 0.243 | 0.757 | 0.000 | 0.235 | 0.765 | 0.000 | 0.253 | 0.747 | 0.000 | 0.244 | 0.756 | 0.000 |
| 207 | 119 | Luchuan | 0.002 | 0.998 | 0.000 | 0.001 | 0.999 | 0.000 | 0.002 | 0.998 | 0.000 | 0.002 | 0.998 | 0.000 |
| 208 | 120 | Luchuan | 0.002 | 0.998 | 0.000 | 0.001 | 0.999 | 0.000 | 0.002 | 0.998 | 0.000 | 0.002 | 0.998 | 0.000 |
| 209 | 121 | Luchuan | 0.002 | 0.998 | 0.000 | 0.000 | 1.000 | 0.000 | 0.002 | 0.998 | 0.000 | 0.001 | 0.999 | 0.000 |
| 210 | 122 | Luchuan | 0.006 | 0.994 | 0.000 | 0.001 | 0.999 | 0.000 | 0.006 | 0.994 | 0.000 | 0.004 | 0.996 | 0.000 |
| 211 | 123 | Luchuan | 0.002 | 0.998 | 0.000 | 0.000 | 1.000 | 0.000 | 0.002 | 0.998 | 0.000 | 0.001 | 0.999 | 0.000 |
| 212 | 124 | Luchuan | 0.012 | 0.988 | 0.000 | 0.005 | 0.995 | 0.000 | 0.013 | 0.987 | 0.000 | 0.010 | 0.990 | 0.000 |
| 213 | 728 | Large  Black-white | 0.083 | 0.917 | 0.000 | 0.065 | 0.935 | 0.000 | 0.085 | 0.915 | 0.000 | 0.078 | 0.922 | 0.000 |
| 214 | 727 | Lantang | 0.110 | 0.889 | 0.000 | 0.077 | 0.922 | 0.000 | 0.103 | 0.896 | 0.001 | 0.097 | 0.903 | 0.000 |
| 215 | 105 | Wuzhishan | 0.079 | 0.921 | 0.000 | 0.067 | 0.933 | 0.000 | 0.074 | 0.926 | 0.000 | 0.073 | 0.927 | 0.000 |
| 216 | 106 | Wuzhishan | 0.066 | 0.934 | 0.000 | 0.054 | 0.946 | 0.000 | 0.065 | 0.935 | 0.000 | 0.062 | 0.938 | 0.000 |
| 217 | 107 | Wuzhishan | 0.088 | 0.912 | 0.000 | 0.071 | 0.929 | 0.000 | 0.085 | 0.915 | 0.000 | 0.081 | 0.919 | 0.000 |
| 218 | 108 | Wuzhishan | 0.063 | 0.936 | 0.000 | 0.032 | 0.968 | 0.000 | 0.060 | 0.940 | 0.000 | 0.052 | 0.948 | 0.000 |
| 219 | 109 | Wuzhishan | 0.071 | 0.929 | 0.000 | 0.058 | 0.942 | 0.000 | 0.075 | 0.925 | 0.000 | 0.068 | 0.932 | 0.000 |
| 220 | 110 | Wuzhishan | 0.023 | 0.976 | 0.001 | 0.013 | 0.986 | 0.001 | 0.020 | 0.979 | 0.001 | 0.019 | 0.980 | 0.001 |
| 221 | 174 | Wuzhishan | 0.060 | 0.940 | 0.000 | 0.037 | 0.963 | 0.000 | 0.061 | 0.939 | 0.000 | 0.053 | 0.947 | 0.000 |
| 222 | 175 | Tongcheng | 0.023 | 0.976 | 0.000 | 0.015 | 0.985 | 0.000 | 0.020 | 0.980 | 0.000 | 0.019 | 0.981 | 0.000 |
| 223 | 176 | Tongcheng | 0.037 | 0.963 | 0.000 | 0.027 | 0.973 | 0.000 | 0.040 | 0.960 | 0.000 | 0.035 | 0.965 | 0.000 |
| 224 | 177 | Tongcheng | 0.005 | 0.995 | 0.000 | 0.002 | 0.998 | 0.000 | 0.005 | 0.995 | 0.000 | 0.004 | 0.996 | 0.000 |
| 225 | 178 | Tongcheng | 0.027 | 0.973 | 0.000 | 0.017 | 0.983 | 0.000 | 0.030 | 0.970 | 0.000 | 0.025 | 0.975 | 0.000 |
| 226 | 730 | Hang | 0.061 | 0.938 | 0.001 | 0.050 | 0.949 | 0.001 | 0.062 | 0.936 | 0.001 | 0.058 | 0.941 | 0.001 |
| 227 | 721 | Putian | 0.191 | 0.809 | 0.000 | 0.154 | 0.846 | 0.000 | 0.180 | 0.820 | 0.000 | 0.175 | 0.825 | 0.000 |
| 228 | 7 | Wild boar  of Sumatra | 0.008 | 0.714 | 0.279 | 0.001 | 0.720 | 0.279 | 0.006 | 0.715 | 0.279 | 0.005 | 0.716 | 0.279 |
| 229 | 9 | Wild boar  of Sumatra | 0.015 | 0.713 | 0.271 | 0.001 | 0.727 | 0.271 | 0.016 | 0.712 | 0.272 | 0.011 | 0.718 | 0.271 |
| 230 | 8 | Sus barbatus | 0.001 | 0.000 | 0.999 | 0.000 | 0.001 | 0.999 | 0.001 | 0.000 | 0.999 | 0.001 | 0.000 | 0.999 |
| 231 | 42 | Sus verrucosus | 0.000 | 0.000 | 1.000 | 0.000 | 0.000 | 1.000 | 0.000 | 0.000 | 1.000 | 0.000 | 0.000 | 1.000 |
| 232 | 41 | Sus celebensis | 0.000 | 0.000 | 1.000 | 0.000 | 0.000 | 1.000 | 0.000 | 0.000 | 1.000 | 0.000 | 0.000 | 1.000 |
| 233 | 40 | Sus cebifrons | 0.000 | 0.000 | 1.000 | 0.000 | 0.000 | 1.000 | 0.000 | 0.000 | 1.000 | 0.000 | 0.000 | 1.000 |

**Supplementary Table s4:** Genetic regions under selection in the Tibetan pig by *F*_ST._

| **Number** | **Chromosome** | **Start Position** | **End Positon** | **Sweep Length** | **SNPs** | **Mean *F*_ST_** | **PSGs** |
| --- | --- | --- | --- | --- | --- | --- | --- |
| 1 | 1 | 46710001 | 46750000 | 40 Kb | 36 | 0.202 | */* |
| 2 | 1 | 52070001 | 52100000 | 30 Kb | 304 | 0.234 | */* |
| 3 | 1 | 83300001 | 83340000 | 40 Kb | 134 | 0.167 | *SEC63* |
| 4 | 1 | 96950001 | 96980000 | 30 Kb | 264 | 0.163 | */* |
| 5 | 1 | 136000001 | 136070000 | 70 Kb | 258 | 0.228 | */* |
| 6 | 1 | 149500001 | 149560000 | 60 Kb | 67 | 0.211 | */* |
| 7 | 1 | 292940001 | 292970000 | 30 Kb | 128 | 0.184 | *ENSSSCG00000005507, ENSSSCG00000005509* |
| 8 | 1 | 292990001 | 293020000 | 30 Kb | 94 | 0.201 | *PHF19* |
| 9 | 2 | 27060001 | 27110000 | 50 Kb | 125 | 0.203 | */* |
| 10 | 2 | 59180001 | 59230000 | 50 Kb | 99 | 0.183 | *ENSSSCG00000029924, IFI30,PIK3R2* |
| 11 | 2 | 61350001 | 61380000 | 30 Kb | 316 | 0.287 | *CYP4F2* |
| 12 | 2 | 76370001 | 76400000 | 30 Kb | 134 | 0.195 | *SGTA,THOP1* |
| 13 | 2 | 78120001 | 78180000 | 60 Kb | 102 | 0.220 | *ENSSSCG00000013434, MBD3,MEX3D,UQCR11* |
| 14 | 2 | 80660001 | 80690000 | 30 Kb | 121 | 0.198 | */* |
| 15 | 2 | 82510001 | 82560000 | 50 Kb | 122 | 0.160 | *UIMC1* |
| 16 | 3 | 10001 | 100000 | 90 Kb | 462 | 0.224 | */* |
| 17 | 3 | 7020001 | 7050000 | 30 Kb | 54 | 0.384 | *ENSSSCG00000030382* |
| 18 | 3 | 38330001 | 38370000 | 40 Kb | 120 | 0.180 | *ENSSSCG00000007919, ENSSSCG00000025072* |
| 19 | 3 | 44170001 | 44250000 | 80 Kb | 22 | 0.244 | *ENSSSCG00000025899, FAM120A* |
| 20 | 3 | 44410001 | 44440000 | 30 Kb | 26 | 0.193 | *PHF2* |
| 21 | 3 | 58220001 | 58260000 | 40 Kb | 109 | 0.208 | *COA5,UNC50* |
| 22 | 3 | 100170001 | 100240000 | 70 Kb | 572 | 0.231 | *EPAS1* |
| 23 | 4 | 55410001 | 55440000 | 30 Kb | 79 | 0.188 | */* |
| 24 | 4 | 55600001 | 55630000 | 30 Kb | 185 | 0.170 | *SLC7A13* |
| 25 | 5 | 1950001 | 1980000 | 30 Kb | 41 | 0.201 | */* |
| 26 | 5 | 3960001 | 4060000 | 100 Kb | 43 | 0.192 | *ENSSSCG00000023108, NAGA,NDUFA6* |
| 27 | 5 | 13450001 | 13540000 | 90 Kb | 529 | 0.194 | *RFX4* |
| 28 | 5 | 19650001 | 19680000 | 30 Kb | 32 | 0.184 | *HOXC10* |
| 29 | 5 | 48610001 | 48640000 | 30 Kb | 110 | 0.177 | *CCDC91* |
| 30 | 5 | 81490001 | 81520000 | 30 Kb | 42 | 0.376 | *TMEM106C* |
| 31 | 6 | 48990001 | 49020000 | 30 Kb | 49 | 0.172 | *SEPW1* |
| 32 | 6 | 50410001 | 50460000 | 50 Kb | 61 | 0.193 | *BCL2L12,PRMT1, RRAS,SCAF1* |
| 33 | 6 | 62510001 | 62540000 | 30 Kb | 56 | 0.196 | */* |
| 34 | 6 | 65180001 | 65210000 | 30 Kb | 151 | 0.170 | *EXOSC10,FRAP1* |
| 35 | 6 | 66960001 | 67050000 | 90 Kb | 28 | 0.351 | *ENSSSCG00000003446* |
| 36 | 6 | 123160001 | 123210000 | 50 Kb | 157 | 0.195 | */* |
| 37 | 6 | 127410001 | 127480000 | 70 Kb | 124 | 0.234 | *MSH4* |
| 38 | 6 | 131270001 | 131300000 | 30 Kb | 94 | 0.175 | */* |
| 39 | 6 | 131310001 | 131340000 | 30 Kb | 142 | 0.201 | */* |
| 40 | 7 | 44050001 | 44080000 | 30 Kb | 103 | 0.211 | */* |
| 41 | 7 | 72680001 | 72720000 | 40 Kb | 192 | 0.168 | *ENSSSCG00000026310* |
| 42 | 7 | 80060001 | 80110000 | 50 Kb | 101 | 0.189 | *ENSSSCG00000001982* |
| 43 | 8 | 49440001 | 49470000 | 30 Kb | 165 | 0.177 | */* |
| 44 | 8 | 80310001 | 80340000 | 30 Kb | 15 | 0.221 | *TRIM2* |
| 45 | 8 | 91770001 | 91820000 | 50 Kb | 288 | 0.212 | */* |
| 46 | 8 | 91920001 | 91960000 | 40 Kb | 283 | 0.239 | *ENSSSCG00000009056* |
| 47 | 8 | 92110001 | 92240000 | 130 Kb | 415 | 0.212 | *CLGN* |
| 48 | 8 | 92250001 | 92280000 | 30 Kb | 101 | 0.225 | */* |
| 49 | 8 | 116800001 | 116830000 | 30 Kb | 213 | 0.164 | */* |
| 50 | 8 | 133540001 | 133570000 | 30 Kb | 195 | 0.215 | */* |
| 51 | 9 | 34220001 | 34270000 | 50 Kb | 389 | 0.187 | */* |
| 52 | 9 | 88910001 | 88970000 | 60 Kb | 183 | 0.280 | *THSD7A* |
| 53 | 9 | 89010001 | 89130000 | 120 Kb | 213 | 0.235 | *THSD7A* |
| 54 | 9 | 89430001 | 89480000 | 50 Kb | 232 | 0.208 | */* |
| 55 | 9 | 89750001 | 89780000 | 30 Kb | 124 | 0.219 | */* |
| 56 | 9 | 91680001 | 91710000 | 30 Kb | 94 | 0.163 | */* |
| 57 | 9 | 94360001 | 94400000 | 40 Kb | 433 | 0.194 | *ENSSSCG00000027138, ENSSSCG00000029648* |
| 58 | 9 | 95380001 | 95410000 | 30 Kb | 50 | 0.205 | *ENSSSCG00000015364* |
| 59 | 9 | 95580001 | 95610000 | 30 Kb | 113 | 0.197 | */* |
| 60 | 9 | 95680001 | 95750000 | 70 Kb | 54 | 0.165 | */* |
| 61 | 9 | 116660001 | 116690000 | 30 Kb | 187 | 0.189 | *ENSSSCG00000015434* |
| 62 | 9 | 120330001 | 120360000 | 30 Kb | 85 | 0.213 | *EZH2* |
| 63 | 10 | 38350001 | 38380000 | 30 Kb | 124 | 0.289 | */* |
| 64 | 11 | 17790001 | 17850000 | 60 Kb | 129 | 0.194 | */* |
| 65 | 11 | 19230001 | 19270000 | 40 Kb | 244 | 0.210 | */* |
| 66 | 11 | 35830001 | 35870000 | 40 Kb | 240 | 0.217 | */* |
| 67 | 11 | 63520001 | 63550000 | 30 Kb | 8 | 0.286 | */* |
| 68 | 12 | 56380001 | 56420000 | 40 Kb | 29 | 0.236 | */* |
| 69 | 13 | 7650001 | 7680000 | 30 Kb | 593 | 0.230 | */* |
| 70 | 13 | 48350001 | 48400000 | 50 Kb | 327 | 0.201 | *ENSSSCG00000011489* |
| 71 | 13 | 69310001 | 69360000 | 50 Kb | 6 | 0.187 | */* |
| 72 | 13 | 144990001 | 145020000 | 30 Kb | 199 | 0.223 | *MUC13* |
| 73 | 13 | 159250001 | 159280000 | 30 Kb | 110 | 0.215 | *DPPA4* |
| 74 | 13 | 160300001 | 160340000 | 40 Kb | 76 | 0.162 | *IFT57* |
| 75 | 13 | 162710001 | 162740000 | 30 Kb | 76 | 0.172 | *CBLB* |
| 76 | 14 | 45250001 | 45280000 | 30 Kb | 177 | 0.163 | */* |
| 77 | 14 | 45690001 | 45720000 | 30 Kb | 63 | 0.223 | *SGSM1,TMEM211* |
| 78 | 14 | 45760001 | 45810000 | 50 Kb | 113 | 0.247 | */* |
| 79 | 14 | 91100001 | 91140000 | 40 Kb | 162 | 0.191 | *NRG3* |
| 80 | 14 | 104860001 | 104910000 | 50 Kb | 263 | 0.184 | */* |
| 81 | 14 | 104920001 | 104950000 | 30 Kb | 123 | 0.173 | */* |
| 82 | 14 | 134110001 | 134140000 | 30 Kb | 16 | 0.204 | */* |
| 83 | 15 | 55790001 | 55830000 | 40 Kb | 163 | 0.240 | *ZNF703* |
| 84 | 15 | 56610001 | 56660000 | 50 Kb | 26 | 0.186 | */* |
| 85 | 15 | 58160001 | 58200000 | 40 Kb | 247 | 0.161 | */* |
| 86 | 15 | 58860001 | 58890000 | 30 Kb | 45 | 0.178 | */* |
| 87 | 15 | 59620001 | 59650000 | 30 Kb | 285 | 0.266 | */* |
| 88 | 15 | 73530001 | 73560000 | 30 Kb | 55 | 0.239 | *BAZ2B* |
| 89 | 15 | 73640001 | 73680000 | 40 Kb | 74 | 0.210 | */* |
| 90 | 15 | 74080001 | 74160000 | 80 Kb | 235 | 0.214 | *PLA2R1* |
| 91 | 15 | 74250001 | 74280000 | 30 Kb | 113 | 0.212 | *PLA2R1* |
| 92 | 15 | 80650001 | 80690000 | 40 Kb | 77 | 0.238 | *CSRNP3* |
| 93 | 15 | 80800001 | 80840000 | 40 Kb | 85 | 0.204 | *GALNT3* |
| 94 | 15 | 82540001 | 82570000 | 30 Kb | 84 | 0.161 |  |
| 95 | 15 | 85190001 | 85230000 | 40 Kb | 209 | 0.225 | *METTL5,SSB* |
| 96 | 15 | 96990001 | 97030000 | 40 Kb | 165 | 0.207 | *PDE1A* |
| 97 | 15 | 97210001 | 97480000 | 270 Kb | 396 | 0.215 | *ENSSSCG00000029344* |
| 98 | 15 | 113960001 | 113990000 | 30 Kb | 165 | 0.242 | */* |
| 99 | 16 | 27360001 | 27390000 | 30 Kb | 200 | 0.246 | */* |
| 100 | 16 | 41710001 | 41740000 | 30 Kb | 164 | 0.211 | */* |
| 101 | 16 | 42070001 | 42100000 | 30 Kb | 133 | 0.155 | */* |
| 102 | 16 | 54130001 | 54160000 | 30 Kb | 79 | 0.213 | */* |
| 103 | 17 | 41170001 | 41200000 | 30 Kb | 102 | 0.210 | */* |
| 104 | 18 | 41380001 | 41440000 | 60 Kb | 140 | 0.254 | *KIAA0895* |

**Supplementary Table s5:** Genetic regions under selection in the Tibetan pig by XP-EHH.

| **Number** | **Chromosome** | **Start Position** | **End Position** | **Sweep Length** | **SNPs** | **Mean XP-EHH** | **PSGs** |
| --- | --- | --- | --- | --- | --- | --- | --- |
| 1 | 1 | 1890001 | 1920000 | 30 Kb | 163 | 0.710 | */* |
| 2 | 1 | 12380001 | 12410000 | 30 Kb | 131 | 0.857 | */* |
| 3 | 1 | 17140001 | 17180000 | 40 Kb | 261 | 0.717 | *C6ORF97* |
| 4 | 1 | 19420001 | 19480000 | 60 Kb | 562 | 0.782 | *UST* |
| 5 | 1 | 24100001 | 24150000 | 50 Kb | 433 | 0.742 | *FUCA2* |
| 6 | 1 | 40700001 | 40820000 | 120 Kb | 463 | 0.847 | *HINT3,NCOA7,TRMT11* |
| 7 | 1 | 47790001 | 47860000 | 70 Kb | 442 | 0.938 | */* |
| 8 | 1 | 62860001 | 62900000 | 40 Kb | 246 | 0.791 | */* |
| 9 | 1 | 64040001 | 64070000 | 30 Kb | 210 | 0.691 | *GABRR2* |
| 10 | 1 | 74920001 | 75000000 | 80 Kb | 484 | 0.791 | *COQ3,ENSSSCG00000004348, ENSSSCG00000023154* |
| 11 | 1 | 89160001 | 89190000 | 30 Kb | 182 | 0.735 | */* |
| 12 | 1 | 95050001 | 95100000 | 50 Kb | 295 | 0.851 | */* |
| 13 | 1 | 110640001 | 110690000 | 50 Kb | 254 | 0.874 | */* |
| 14 | 1 | 110730001 | 110760000 | 30 Kb | 138 | 0.810 | */* |
| 15 | 1 | 124120001 | 124200000 | 80 Kb | 499 | 0.807 | */* |
| 16 | 1 | 124380001 | 124420000 | 40 Kb | 233 | 0.787 | *GCNT3* |
| 17 | 1 | 126990001 | 127040000 | 50 Kb | 159 | 0.828 | *TCF12* |
| 18 | 1 | 138790001 | 138850000 | 60 Kb | 228 | 0.965 |  |
| 19 | 1 | 143380001 | 143410000 | 30 Kb | 126 | 0.769 | *TTBK2* |
| 20 | 1 | 143860001 | 143910000 | 50 Kb | 240 | 0.708 | *CAPN3* |
| 21 | 1 | 145260001 | 145300000 | 40 Kb | 97 | 0.667 | *EXD1* |
| 22 | 1 | 145450001 | 145490000 | 40 Kb | 126 | 0.729 | *INO80* |
| 23 | 1 | 146210001 | 146240000 | 30 Kb | 128 | 0.735 | *BUB1B,ENSSSCG00000029381* |
| 24 | 1 | 160250001 | 160280000 | 30 Kb | 167 | 0.788 | *ENSSSCG00000004849* |
| 25 | 1 | 165230001 | 165360000 | 130 Kb | 569 | 0.950 | */* |
| 26 | 1 | 165520001 | 165630000 | 110 Kb | 555 | 1.045 | *CNDP1,CNDP2* |
| 27 | 1 | 165660001 | 165690000 | 30 Kb | 79 | 0.776 | */* |
| 28 | 1 | 173920001 | 174020000 | 100 Kb | 380 | 0.740 | */* |
| 29 | 1 | 179270001 | 179380000 | 110 Kb | 968 | 0.729 | *ENSSSCG00000004911* |
| 30 | 1 | 202060001 | 202140000 | 80 Kb | 347 | 0.919 | */* |
| 31 | 1 | 212150001 | 212180000 | 30 Kb | 46 | 0.738 | *HIF1A* |
| 32 | 1 | 213090001 | 213120000 | 30 Kb | 215 | 0.727 | *NTRK3* |
| 33 | 1 | 216070001 | 216100000 | 30 Kb | 126 | 0.798 | */* |
| 34 | 1 | 228940001 | 228980000 | 40 Kb | 331 | 0.789 | *SH3GL2* |
| 35 | 1 | 242930001 | 242960000 | 30 Kb | 192 | 0.710 | */* |
| 36 | 1 | 266960001 | 267000000 | 40 Kb | 199 | 0.778 | */* |
| 37 | 1 | 267020001 | 267110000 | 90 Kb | 544 | 0.948 | */* |
| 38 | 1 | 282040001 | 282080000 | 40 Kb | 304 | 0.880 | *TXN,TXNDC8* |
| 39 | 2 | 17120001 | 17190000 | 70 Kb | 487 | 0.865 | *ENSSSCG00000013249,F2, ENSSSCG00000029680,ZNF408* |
| 40 | 2 | 34550001 | 34620000 | 70 Kb | 314 | 0.822 | *METT5D1* |
| 41 | 2 | 42200001 | 42230000 | 30 Kb | 369 | 0.826 | *NAV2* |
| 42 | 2 | 44180001 | 44210000 | 30 Kb | 264 | 0.717 | *SERGEF* |
| 43 | 2 | 47610001 | 47660000 | 50 Kb | 255 | 0.738 | *ENSSSCG00000013388,PDE3B* |
| 44 | 2 | 47950001 | 47980000 | 30 Kb | 115 | 0.769 | *PSMA1* |
| 45 | 2 | 49360001 | 49390000 | 30 Kb | 157 | 0.824 | */* |
| 46 | 2 | 50710001 | 50740000 | 30 Kb | 274 | 0.780 | *DKK3* |
| 47 | 2 | 72710001 | 72750000 | 40 Kb | 160 | 0.745 | *EMR1* |
| 48 | 2 | 73470001 | 73550000 | 80 Kb | 240 | 0.794 | *ACSBG2,ENSSSCG00000026403* |
| 49 | 2 | 85730001 | 85810000 | 80 Kb | 589 | 0.735 | *ANKRD31,ENSSSCG00000014078* |
| 50 | 2 | 86590001 | 86630000 | 40 Kb | 180 | 0.698 | */* |
| 51 | 2 | 122700001 | 122770000 | 70 Kb | 543 | 0.910 | *ENSSSCG00000014212* |
| 52 | 2 | 136290001 | 136350000 | 60 Kb | 531 | 0.869 | */* |
| 53 | 3 | 2030001 | 2060000 | 30 Kb | 119 | 0.739 | *AMZ1* |
| 54 | 3 | 31260001 | 31320000 | 60 Kb | 389 | 0.813 | */* |
| 55 | 3 | 35950001 | 36020000 | 70 Kb | 502 | 0.715 | *RBFOX1* |
| 56 | 3 | 48100001 | 48130000 | 30 Kb | 190 | 0.818 | *MRPS5* |
| 57 | 3 | 48200001 | 48250000 | 50 Kb | 304 | 0.891 | */* |
| 58 | 3 | 67580001 | 67620000 | 40 Kb | 207 | 0.958 | */* |
| 59 | 3 | 80470001 | 80510000 | 40 Kb | 263 | 0.760 | *ACTR2* |
| 60 | 3 | 103190001 | 103220000 | 30 Kb | 199 | 0.947 | *THADA* |
| 61 | 3 | 139020001 | 139070000 | 50 Kb | 440 | 0.888 | */* |
| 62 | 3 | 140300001 | 140340000 | 40 Kb | 472 | 0.707 | */* |
| 63 | 3 | 140860001 | 140890000 | 30 Kb | 139 | 0.752 | */* |
| 64 | 4 | 19450001 | 19490000 | 40 Kb | 425 | 0.841 | *SNTB1* |
| 65 | 4 | 25830001 | 25980000 | 150 Kb | 582 | 0.892 | */* |
| 66 | 4 | 44640001 | 44680000 | 40 Kb | 348 | 0.854 | *U6* |
| 67 | 4 | 54080001 | 54110000 | 30 Kb | 47 | 0.707 | */* |
| 68 | 4 | 54810001 | 54840000 | 30 Kb | 134 | 0.772 | */* |
| 69 | 4 | 56070001 | 56120000 | 50 Kb | 273 | 0.739 | */* |
| 70 | 4 | 56220001 | 56250000 | 30 Kb | 174 | 0.733 | */* |
| 71 | 4 | 60320001 | 60390000 | 70 Kb | 450 | 0.764 | */* |
| 72 | 4 | 137710001 | 137740000 | 30 Kb | 168 | 0.759 | *BARHL2* |
| 73 | 4 | 141510001 | 141580000 | 70 Kb | 690 | 0.943 | *HS2ST1* |
| 74 | 5 | 9470001 | 9500000 | 30 Kb | 343 | 0.831 | *ENSSSCG00000028256* |
| 75 | 5 | 13450001 | 13560000 | 110 Kb | 1026 | 1.090 | *RFX4* |
| 76 | 5 | 23310001 | 23350000 | 40 Kb | 218 | 0.737 | *MIP,TIMELESS* |
| 77 | 5 | 23390001 | 23440000 | 50 Kb | 107 | 0.704 | *RBMS2* |
| 78 | 5 | 66240001 | 66280000 | 40 Kb | 286 | 0.817 | *ENO2,LRRC23,SPSB2* |
| 79 | 5 | 70460001 | 70490000 | 30 Kb | 213 | 0.867 | */* |
| 80 | 5 | 74660001 | 74710000 | 50 Kb | 185 | 0.726 | *MUC19* |
| 81 | 5 | 83790001 | 83820000 | 30 Kb | 168 | 0.682 | */* |
| 82 | 5 | 90220001 | 90260000 | 40 Kb | 250 | 0.746 | */* |
| 83 | 6 | 41750001 | 41790000 | 40 Kb | 258 | 0.712 | *ZNF527* |
| 84 | 6 | 41810001 | 41900000 | 90 Kb | 351 | 0.804 | *ENSSSCG00000023635,ZNF420* |
| 85 | 6 | 42410001 | 42450000 | 40 Kb | 168 | 0.691 | *ENSSSCG00000023833* |
| 86 | 6 | 42590001 | 42620000 | 30 Kb | 148 | 0.702 | *ENSSSCG00000002950, ENSSSCG00000029134* |
| 87 | 6 | 42680001 | 42710000 | 30 Kb | 153 | 0.732 | *SPINT2* |
| 88 | 6 | 42760001 | 42820000 | 60 Kb | 196 | 0.773 | *CATSPERG,FAM98C,GGN, PSMD8,SPRED3* |
| 89 | 6 | 63850001 | 63880000 | 30 Kb | 166 | 0.760 | */* |
| 90 | 6 | 64550001 | 64600000 | 50 Kb | 232 | 0.845 | *KIF1B* |
| 91 | 6 | 65320001 | 65370000 | 50 Kb | 345 | 0.811 | *UBIAD1* |
| 92 | 6 | 65820001 | 65890000 | 70 Kb | 420 | 0.731 | *CLCN6,NPPB* |
| 93 | 6 | 80940001 | 80970000 | 30 Kb | 270 | 0.797 | */* |
| 94 | 6 | 81990001 | 82020000 | 30 Kb | 128 | 0.778 | *COL16A1* |
| 95 | 6 | 86270001 | 86300000 | 30 Kb | 321 | 0.857 | */* |
| 96 | 6 | 87820001 | 87860000 | 40 Kb | 219 | 0.872 | *MYCBP,RRAGC* |
| 97 | 6 | 88900001 | 88960000 | 60 Kb | 330 | 0.743 | *MFSD2A* |
| 98 | 6 | 89770001 | 89820000 | 50 Kb | 384 | 0.940 | *C18orf1* |
| 99 | 6 | 101770001 | 101810000 | 40 Kb | 166 | 0.720 | *OSBPL1A* |
| 100 | 6 | 102670001 | 102730000 | 60 Kb | 225 | 0.857 | */* |
| 101 | 6 | 110970001 | 111020000 | 50 Kb | 318 | 0.828 | *ENSSSCG00000029762* |
| 102 | 6 | 129140001 | 129170000 | 30 Kb | 176 | 0.741 | */* |
| 103 | 6 | 146340001 | 146370000 | 30 Kb | 275 | 0.733 | */* |
| 104 | 6 | 148210001 | 148240000 | 30 Kb | 327 | 0.838 | *OSBPL9* |
| 105 | 6 | 148350001 | 148400000 | 50 Kb | 404 | 0.873 | *EPS15* |
| 106 | 7 | 49580001 | 49610000 | 30 Kb | 277 | 0.839 | */* |
| 107 | 7 | 55570001 | 55600000 | 30 Kb | 282 | 0.878 | *ENSSSCG00000030389* |
| 108 | 7 | 69470001 | 69530000 | 60 Kb | 373 | 0.749 | *PSMA6* |
| 109 | 7 | 73480001 | 73510000 | 30 Kb | 109 | 0.721 | *G2E3* |
| 110 | 7 | 80100001 | 80150000 | 50 Kb | 167 | 0.919 | *CBLN3,KHNYN,SDR39U1* |
| 111 | 7 | 80240001 | 80310000 | 70 Kb | 341 | 0.960 | *C14ORF21,CIDEB,DHRS1, RABGGTA,TGM1* |
| 112 | 7 | 120700001 | 120730000 | 30 Kb | 267 | 0.740 | *RIN3* |
| 113 | 7 | 123260001 | 123290000 | 30 Kb | 280 | 0.744 | */* |
| 114 | 7 | 130480001 | 130530000 | 50 Kb | 277 | 0.985 | */* |
| 115 | 8 | 880001 | 930000 | 50 Kb | 232 | 0.764 | *RNF4* |
| 116 | 8 | 1210001 | 1260000 | 50 Kb | 297 | 0.809 | *ADD1* |
| 117 | 8 | 1470001 | 1530000 | 60 Kb | 276 | 0.873 | *HTT* |
| 118 | 8 | 50580001 | 50640000 | 60 Kb | 190 | 0.759 | *RAPGEF2* |
| 119 | 8 | 91200001 | 91240000 | 40 Kb | 211 | 0.947 | */* |
| 120 | 8 | 91320001 | 91360000 | 40 Kb | 203 | 0.770 | *ZNF330* |
| 121 | 8 | 91450001 | 91490000 | 40 Kb | 323 | 0.965 | */* |
| 122 | 8 | 91570001 | 91700000 | 130 Kb | 788 | 0.913 | *ENSSSCG00000027137,RNF150* |
| 123 | 8 | 91760001 | 91800000 | 40 Kb | 257 | 0.849 | */* |
| 124 | 8 | 91900001 | 92010000 | 110 Kb | 799 | 1.133 | *ELMOD2,ENSSSCG00000009056* |
| 125 | 8 | 92060001 | 92230000 | 170 Kb | 1270 | 1.232 | *CLGN,ENSSSCG00000009054, ENSSSCG00000025882* |
| 126 | 8 | 92320001 | 92390000 | 70 Kb | 561 | 0.766 | */* |
| 127 | 8 | 107950001 | 107990000 | 40 Kb | 194 | 0.844 | */* |
| 128 | 8 | 122130001 | 122210000 | 80 Kb | 293 | 0.804 | *LEF1* |
| 129 | 8 | 130490001 | 130560000 | 70 Kb | 612 | 0.874 | *METAP1* |
| 130 | 8 | 130580001 | 130630000 | 50 Kb | 300 | 0.968 | *EIF4E* |
| 131 | 9 | 450001 | 480000 | 30 Kb | 113 | 0.683 | *SCUBE2* |
| 132 | 9 | 17310001 | 17340000 | 30 Kb | 183 | 0.683 | */* |
| 133 | 9 | 25400001 | 25430000 | 30 Kb | 181 | 0.727 | *NOX4* |
| 134 | 9 | 34350001 | 34380000 | 30 Kb | 110 | 0.688 | */* |
| 135 | 9 | 34490001 | 34530000 | 40 Kb | 139 | 0.844 | */* |
| 136 | 9 | 34550001 | 34580000 | 30 Kb | 157 | 0.727 | */* |
| 137 | 9 | 40490001 | 40520000 | 30 Kb | 191 | 0.786 | *CUL5* |
| 138 | 9 | 42960001 | 42990000 | 30 Kb | 200 | 0.711 | *RDX* |
| 139 | 9 | 44370001 | 44400000 | 30 Kb | 288 | 0.902 | *ENSSSCG00000015023, ENSSSCG00000022950* |
| 140 | 9 | 58630001 | 58730000 | 100 Kb | 339 | 0.829 | *ENSSSCG00000015220, ENSSSCG00000015222* |
| 141 | 9 | 61440001 | 61470000 | 30 Kb | 233 | 0.741 | */* |
| 142 | 9 | 78840001 | 78880000 | 40 Kb | 148 | 0.738 | *KRIT1,LRRD1* |
| 143 | 9 | 79120001 | 79220000 | 100 Kb | 457 | 0.790 | *ENSSSCG00000015316, C7ORF64,FAM133B,PEX1* |
| 144 | 9 | 88780001 | 88920000 | 140 Kb | 715 | 0.920 | *PHF14* |
| 145 | 9 | 100660001 | 100690000 | 30 Kb | 154 | 0.956 | */* |
| 146 | 9 | 102720001 | 102750000 | 30 Kb | 220 | 0.828 | *ABCB4,CROT* |
| 147 | 9 | 103200001 | 103260000 | 60 Kb | 417 | 0.880 | *GRM3,KIAA1324L* |
| 148 | 10 | 30650001 | 30700000 | 50 Kb | 416 | 0.885 | */* |
| 149 | 10 | 46970001 | 47000000 | 30 Kb | 440 | 0.811 | */* |
| 150 | 10 | 49670001 | 49700000 | 30 Kb | 280 | 0.748 | *NSUN6* |
| 151 | 10 | 57410001 | 57440000 | 30 Kb | 175 | 0.943 | *C10orf67* |
| 152 | 11 | 17740001 | 17770000 | 30 Kb | 118 | 0.763 | */* |
| 153 | 11 | 17780001 | 17810000 | 30 Kb | 82 | 0.782 | */* |
| 154 | 11 | 18860001 | 18960000 | 100 Kb | 947 | 0.858 | *CDADC1,MLNR* |
| 155 | 11 | 19350001 | 19380000 | 30 Kb | 211 | 0.851 | */* |
| 156 | 11 | 19430001 | 19510000 | 80 Kb | 720 | 0.939 | *CYSLTR2* |
| 157 | 11 | 22790001 | 22840000 | 50 Kb | 331 | 0.885 | *ENSSSCG00000009422* |
| 158 | 11 | 22850001 | 22880000 | 30 Kb | 267 | 0.756 | *ENSSSCG00000009422* |
| 159 | 11 | 59680001 | 59710000 | 30 Kb | 110 | 0.716 | */* |
| 160 | 12 | 14770001 | 14810000 | 40 Kb | 384 | 0.766 | *TEX2* |
| 161 | 12 | 54650001 | 54690000 | 40 Kb | 357 | 0.700 | *SNORA11* |
| 162 | 13 | 25890001 | 25940000 | 50 Kb | 330 | 0.841 | */* |
| 163 | 13 | 33450001 | 33590000 | 140 Kb | 632 | 0.790 | *C3ORF75,PTPN23,SCAP* |
| 164 | 13 | 48360001 | 48400000 | 40 Kb | 266 | 0.759 | *ENSSSCG00000011489* |
| 165 | 13 | 60560001 | 60590000 | 30 Kb | 251 | 0.716 | */* |
| 166 | 13 | 90280001 | 90420000 | 140 Kb | 738 | 0.766 | *GRK7,RNF7* |
| 167 | 13 | 90510001 | 90550000 | 40 Kb | 171 | 0.688 | *ENSSSCG00000011676* |
| 168 | 13 | 91380001 | 91480000 | 100 Kb | 530 | 0.750 | */* |
| 169 | 13 | 102240001 | 102390000 | 150 Kb | 589 | 0.835 | *ARHGEF26,DHX36* |
| 170 | 13 | 105860001 | 105990000 | 130 Kb | 292 | 1.032 | */* |
| 171 | 13 | 106140001 | 106220000 | 80 Kb | 205 | 0.724 | *ENSSSCG00000027556* |
| 172 | 13 | 109560001 | 109600000 | 40 Kb | 223 | 0.749 | */* |
| 173 | 13 | 110390001 | 110440000 | 50 Kb | 157 | 0.695 | */* |
| 174 | 13 | 119610001 | 119690000 | 80 Kb | 278 | 0.790 | */* |
| 175 | 13 | 119750001 | 119790000 | 40 Kb | 169 | 0.864 | *FNDC3B* |
| 176 | 13 | 119880001 | 119960000 | 80 Kb | 410 | 0.775 | *TNFSF10* |
| 177 | 13 | 133580001 | 133620000 | 40 Kb | 278 | 0.798 | */* |
| 178 | 13 | 142040001 | 142140000 | 100 Kb | 656 | 0.957 | *APOD,PPP1R2* |
| 179 | 13 | 142180001 | 142210000 | 30 Kb | 201 | 0.815 | */* |
| 180 | 13 | 142560001 | 142730000 | 170 Kb | 281 | 0.790 | *DLG1,MFI2* |
| 181 | 13 | 142880001 | 142930000 | 50 Kb | 371 | 0.755 | *ENSSSCG00000029291* |
| 182 | 13 | 159460001 | 159550000 | 90 Kb | 389 | 0.840 | *MORC1* |
| 183 | 13 | 159730001 | 159840000 | 110 Kb | 363 | 0.830 | *TRAT1* |
| 184 | 13 | 162730001 | 162840000 | 110 Kb | 397 | 0.781 | *CBLB* |
| 185 | 13 | 162870001 | 162900000 | 30 Kb | 106 | 0.727 | *CBLB* |
| 186 | 13 | 166990001 | 167080000 | 90 Kb | 474 | 0.777 | *ENSSSCG00000028677, 5S_rRNA,SENP7* |
| 187 | 13 | 168150001 | 168180000 | 30 Kb | 141 | 0.850 | *ENSSSCG00000011965, ENSSSCG00000011966* |
| 188 | 13 | 177360001 | 177390000 | 30 Kb | 134 | 0.856 | */* |
| 189 | 13 | 184300001 | 184360000 | 60 Kb | 136 | 0.735 | */* |
| 190 | 13 | 184590001 | 184620000 | 30 Kb | 91 | 0.694 | */* |
| 191 | 13 | 214960001 | 214990000 | 30 Kb | 212 | 0.693 | *TMPRSS2* |
| 192 | 14 | 28010001 | 28060000 | 50 Kb | 514 | 0.887 | *TMEM132C* |
| 193 | 14 | 29690001 | 29720000 | 30 Kb | 267 | 0.784 | */* |
| 194 | 14 | 31000001 | 31060000 | 60 Kb | 304 | 0.901 | *DDX55,EIF2B1, GTF2H3,TCTN2* |
| 195 | 14 | 37560001 | 37590000 | 30 Kb | 232 | 0.763 | *FBXO21* |
| 196 | 14 | 38430001 | 38470000 | 40 Kb | 145 | 0.771 | */* |
| 197 | 14 | 49340001 | 49410000 | 70 Kb | 293 | 0.808 | *EMID1,EWSR1,RHBDD3* |
| 198 | 14 | 49660001 | 49700000 | 40 Kb | 138 | 0.685 | *NF2,NIPSNAP1* |
| 199 | 14 | 49850001 | 49910000 | 60 Kb | 273 | 0.768 | *ENSSSCG00000025186* |
| 200 | 14 | 49970001 | 50020000 | 50 Kb | 171 | 0.832 | *MTMR3* |
| 201 | 14 | 51040001 | 51080000 | 40 Kb | 229 | 0.737 | *RNF185* |
| 202 | 14 | 52720001 | 52900000 | 180 Kb | 761 | 0.750 | *BCR,SPECC1L* |
| 203 | 14 | 69070001 | 69110000 | 40 Kb | 150 | 0.735 | */* |
| 204 | 14 | 86480001 | 86510000 | 30 Kb | 261 | 0.962 | */* |
| 205 | 14 | 87980001 | 88020000 | 40 Kb | 351 | 1.026 | */* |
| 206 | 14 | 88030001 | 88100000 | 70 Kb | 558 | 0.832 | *ZMIZ1* |
| 207 | 14 | 90900001 | 90990000 | 90 Kb | 441 | 0.775 | */* |
| 208 | 14 | 91010001 | 91070000 | 60 Kb | 300 | 0.807 | */* |
| 209 | 14 | 94030001 | 94060000 | 30 Kb | 219 | 0.819 | */* |
| 210 | 14 | 96380001 | 96410000 | 30 Kb | 234 | 0.723 | *ENSSSCG00000010377* |
| 211 | 14 | 125900001 | 125980000 | 80 Kb | 749 | 1.067 | */* |
| 212 | 14 | 126010001 | 126070000 | 60 Kb | 517 | 0.786 | *SORCS3* |
| 213 | 14 | 126080001 | 126120000 | 40 Kb | 326 | 0.742 | *SORCS3* |
| 214 | 14 | 140410001 | 140440000 | 30 Kb | 199 | 0.747 | */* |
| 215 | 15 | 10090001 | 10130000 | 40 Kb | 143 | 0.842 | */* |
| 216 | 15 | 42410001 | 42440000 | 30 Kb | 236 | 0.718 | */* |
| 217 | 15 | 53800001 | 53840000 | 40 Kb | 212 | 0.753 | *FAM149A,SNORA31* |
| 218 | 15 | 55210001 | 55240000 | 30 Kb | 174 | 0.750 | */* |
| 219 | 15 | 55790001 | 55840000 | 50 Kb | 320 | 0.789 | */* |
| 220 | 15 | 56570001 | 56620000 | 50 Kb | 273 | 0.858 | */* |
| 221 | 15 | 56650001 | 56680000 | 30 Kb | 112 | 0.733 | */* |
| 222 | 15 | 61270001 | 61300000 | 30 Kb | 141 | 0.729 | */* |
| 223 | 15 | 61370001 | 61410000 | 40 Kb | 135 | 0.919 | *WRN* |
| 224 | 15 | 65930001 | 65970000 | 40 Kb | 115 | 0.720 | *ENSSSCG00000015857* |
| 225 | 15 | 66090001 | 66140000 | 50 Kb | 299 | 0.954 | *SAP130* |
| 226 | 15 | 66200001 | 66320000 | 120 Kb | 420 | 0.950 | *ENSSSCG00000028633, POLR2D,WDR33* |
| 227 | 15 | 66520001 | 66570000 | 50 Kb | 237 | 0.745 | */* |
| 228 | 15 | 66980001 | 67030000 | 50 Kb | 253 | 0.796 | *FMNL2* |
| 229 | 15 | 67630001 | 67660000 | 30 Kb | 134 | 0.944 | */* |
| 230 | 15 | 67680001 | 67720000 | 40 Kb | 127 | 0.808 | */* |
| 231 | 15 | 67870001 | 67930000 | 60 Kb | 293 | 0.861 | */* |
| 232 | 15 | 68080001 | 68110000 | 30 Kb | 65 | 0.743 | */* |
| 233 | 15 | 68330001 | 68380000 | 50 Kb | 141 | 0.758 | */* |
| 234 | 15 | 68490001 | 68570000 | 80 Kb | 248 | 0.934 | */* |
| 235 | 15 | 69030001 | 69100000 | 70 Kb | 167 | 0.772 | *ENSSSCG00000023896* |
| 236 | 15 | 73400001 | 73430000 | 30 Kb | 141 | 0.855 | *TANC1* |
| 237 | 15 | 73580001 | 73610000 | 30 Kb | 104 | 0.802 | *BAZ2B* |
| 238 | 15 | 73770001 | 73890000 | 120 Kb | 367 | 1.004 | *MARCH7* |
| 239 | 15 | 74070001 | 74160000 | 90 Kb | 325 | 1.042 | *PLA2R1* |
| 240 | 15 | 74250001 | 74300000 | 50 Kb | 244 | 0.830 | *PLA2R1* |
| 241 | 15 | 80650001 | 80700000 | 50 Kb | 205 | 0.906 | *CSRNP3,ENSSSCG00000015909* |
| 242 | 15 | 82530001 | 82590000 | 60 Kb | 199 | 0.720 | */* |
| 243 | 15 | 82920001 | 83140000 | 220 Kb | 619 | 1.115 | */* |
| 244 | 15 | 92330001 | 92360000 | 30 Kb | 111 | 0.765 | */* |
| 245 | 15 | 94940001 | 94970000 | 30 Kb | 125 | 0.712 | */* |
| 246 | 15 | 96360001 | 96400000 | 40 Kb | 100 | 0.753 | */* |
| 247 | 15 | 96410001 | 96500000 | 90 Kb | 313 | 0.751 | */* |
| 248 | 15 | 106740001 | 106780000 | 40 Kb | 91 | 0.784 | */* |
| 249 | 15 | 107550001 | 107600000 | 50 Kb | 186 | 0.686 | */* |
| 250 | 15 | 115780001 | 115820000 | 40 Kb | 323 | 0.780 | *AOX1* |
| 251 | 15 | 131690001 | 131720000 | 30 Kb | 329 | 0.758 | */* |
| 252 | 15 | 150870001 | 150900000 | 30 Kb | 252 | 0.711 | */* |
| 253 | 16 | 14960001 | 15050000 | 90 Kb | 325 | 0.741 | */* |
| 254 | 16 | 21980001 | 22020000 | 40 Kb | 293 | 0.720 | */* |
| 255 | 16 | 51710001 | 51780000 | 70 Kb | 287 | 0.821 | *5S_rRNA,BDP1,SMN1, ENSSSCG00000016969* |
| 256 | 16 | 51960001 | 52020000 | 60 Kb | 451 | 0.815 | *CARTPT* |
| 257 | 16 | 52250001 | 52290000 | 40 Kb | 278 | 0.828 | */* |
| 258 | 16 | 75220001 | 75280000 | 60 Kb | 713 | 0.842 | *ENSSSCG00000022569* |
| 259 | 17 | 40650001 | 40720000 | 70 Kb | 515 | 0.796 | *ENSSSCG00000007240, TM9SF4* |
| 260 | 18 | 38170001 | 38200000 | 30 Kb | 253 | 0.753 | */* |
| 261 | 18 | 41340001 | 41370000 | 30 Kb | 130 | 0.744 | *ANLN* |

**Supplementary Table s6:** List of 237 candidate PSGs.

| **ID** | **Gene symbol** | **Description** | ***F*_ST_** | **XP-EHH** |
| --- | --- | --- | --- | --- |
| 1 | *5S_rRNA* | 5S ribosomal RNA |  | **√** |
| 2 | *ABCB4* | ATP-binding cassette, sub-family B (MDR/TAP), member 4 |  | **√** |
| 3 | *ACSBG2* | Acyl-coa synthetase bubblegum family member 2 |  | **√** |
| 4 | *ACTR2* | ARP2 actin-related protein 2 homolog (yeast) |  | **√** |
| 5 | *ADD1* | Adducin 1 (alpha) |  | **√** |
| 6 | *AMZ1* | Archaelysin family metallopeptidase 1 |  | **√** |
| 7 | *ANKRD31* | Ankyrin repeat domain 31 |  | **√** |
| 8 | *ANLN* | Anillin, actin binding protein |  | **√** |
| 9 | *AOX1* | Aldehyde oxidase 1 |  | **√** |
| 10 | *APOD* | Apolipoprotein D |  | **√** |
| 11 | *ARHGEF26* | Rho guanine nucleotide exchange factor (GEF) 26 |  | **√** |
| 12 | *BARHL2* | Barh-like homeobox 2 |  | **√** |
| 13 | *BAZ2B* | Bromodomain adjacent to zinc finger domain, 2B | **√** | **√** |
| 14 | *BCL2L12* | BCL2-like 12 (proline rich) | **√** |  |
| 15 | *BCR* | Breakpoint cluster region |  | **√** |
| 16 | *BDP1* | B double prime 1, subunit of RNA polymerase III transcription initiation factor IIIB |  | **√** |
| 17 | *BUB1B* | BUB1 mitotic checkpoint serine/threonine kinase B |  | **√** |
| 18 | *C10orf67* | Chromosome 10 open reading frame 67 |  | **√** |
| 19 | *C14ORF21* | Chromosome 14 Open Reading Frame 21 |  | **√** |
| 20 | *C18orf1* | Chromosome 18 Open Reading Frame 1 |  | **√** |
| 21 | *C3ORF75* | Chromosome 3 open reading frame 75 |  | **√** |
| 22 | *C6ORF97* | Chromosome 6 open reading frame 97 |  | **√** |
| 23 | *C7ORF64* | Chromosome 7 open reading frame 64 |  | **√** |
| 24 | *CAPN3* | Calpain 3, (p94) |  | **√** |
| 25 | *CARTPT* | CART prepropeptide |  | **√** |
| 26 | *CATSPERG* | Catsper channel auxiliary subunit gamma |  | **√** |
| 27 | *CBLB* | Cbl proto-oncogene B, E3 ubiquitin protein ligase | **√** | **√** |
| 28 | *CBLN3* | Cerebellin 3 precursor |  | **√** |
| 29 | *CCDC91* | Coiled-coil domain containing 91 | **√** |  |
| 30 | *CDADC1* | Cytidine and dcmp deaminase domain containing 1 |  | **√** |
| 31 | *CIDEB* | Cell death-inducing DFFA-like effector b |  | **√** |
| 32 | *CLCN6* | Chloride channel, voltage-sensitive 6 |  | **√** |
| 33 | *CLGN* | Calmegin | **√** | **√** |
| 34 | *CNDP1* | Carnosine dipeptidase 1 (metallopeptidase M20 family) |  | **√** |
| 35 | *CNDP2* | CNDP dipeptidase 2 (metallopeptidase M20 family) |  | **√** |
| 36 | *COA5* | Cytochrome c oxidase assembly factor 5 | **√** |  |
| 37 | *COL16A1* | Collagen, type XVI, alpha 1 |  | **√** |
| 38 | *COQ3* | Coenzyme Q3 methyltransferase |  | **√** |
| 39 | *CROT* | Carnitine O-octanoyltransferase |  | **√** |
| 40 | *CSRNP3* | Cysteine-serine-rich nuclear protein 3 | **√** | **√** |
| 41 | *CUL5* | Cullin 5 |  | **√** |
| 42 | *CYP4F2* | Cytochrome P450, family 4, subfamily F, polypeptide 2 | **√** |  |
| 43 | *CYSLTR2* | Cysteinyl leukotriene receptor 2 |  | **√** |
| 44 | *DDX55* | DEAD (Asp-Glu-Ala-Asp) box polypeptide 55 |  | **√** |
| 45 | *DHRS1* | Dehydrogenase/reductase (SDR family) member 1 |  | **√** |
| 46 | *DHX36* | DEAH (Asp-Glu-Ala-His) box polypeptide 36 |  | **√** |
| 47 | *DKK3* | Dickkopf WNT signaling pathway inhibitor 3 |  | **√** |
| 48 | *DLG1* | Discs, large homolog 1 (Drosophila) |  | **√** |
| 49 | *DPPA4* | Developmental pluripotency associated 4 | **√** |  |
| 50 | *EIF2B1* | Eukaryotic translation initiation factor 2B, subunit 1 alpha, 26kda |  | **√** |
| 51 | *EIF4E* | Eukaryotic translation initiation factor 4E |  | **√** |
| 52 | *ELMOD2* | ELMO/CED-12 domain containing 2 |  | **√** |
| 53 | *EMID1* | EMI domain containing 1 |  | **√** |
| 54 | *EMR1* | Egf-like module containing, mucin-like, hormone receptor-like 1 |  | **√** |
| 55 | *ENO2* | Enolase 2 (gamma, neuronal) |  | **√** |
| 56 | *ENSSSCG00000001982* | Novel gene | **√** |  |
| 57 | *ENSSSCG00000002950* | Novel gene |  | **√** |
| 58 | *ENSSSCG00000003446* | Novel gene | **√** |  |
| 59 | *ENSSSCG00000004348* | Novel gene |  | **√** |
| 60 | *ENSSSCG00000004849* | Novel gene |  | **√** |
| 61 | *ENSSSCG00000004911* | Novel gene |  | **√** |
| 62 | *ENSSSCG00000005507* | Novel gene | **√** |  |
| 63 | *ENSSSCG00000005509* | Novel gene | **√** |  |
| 64 | *ENSSSCG00000007240* | Novel gene |  | **√** |
| 65 | *ENSSSCG00000007919* | Novel gene | **√** |  |
| 66 | *ENSSSCG00000009054* | Novel gene |  | **√** |
| 67 | *ENSSSCG00000009056* | Novel gene | **√** | **√** |
| 68 | *ENSSSCG00000009422* | Novel gene |  | **√** |
| 69 | *ENSSSCG00000010377* | Novel gene |  | **√** |
| 70 | *ENSSSCG00000011489* | Novel gene | **√** | **√** |
| 71 | *ENSSSCG00000011676* | Novel gene |  | **√** |
| 72 | *ENSSSCG00000011965* | Novel gene |  | **√** |
| 73 | *ENSSSCG00000011966* | Novel gene |  | **√** |
| 74 | *ENSSSCG00000013249* | Novel gene |  | **√** |
| 75 | *ENSSSCG00000013388* | Novel gene |  | **√** |
| 76 | *ENSSSCG00000013434* | Novel gene | **√** |  |
| 77 | *ENSSSCG00000014078* | Novel gene |  | **√** |
| 78 | *ENSSSCG00000014212* | Novel gene |  | **√** |
| 79 | *ENSSSCG00000015023* | Novel gene |  | **√** |
| 80 | *ENSSSCG00000015220* | Novel gene |  | **√** |
| 81 | *ENSSSCG00000015222* | Novel gene |  | **√** |
| 82 | *ENSSSCG00000015316* | Novel gene |  | **√** |
| 83 | *ENSSSCG00000015364* | Novel gene | **√** |  |
| 84 | *ENSSSCG00000015434* | Novel gene | **√** |  |
| 85 | *ENSSSCG00000015857* | Novel gene |  | **√** |
| 86 | *ENSSSCG00000015909* | Novel gene |  | **√** |
| 87 | *ENSSSCG00000016969* | Novel gene |  | **√** |
| 88 | *ENSSSCG00000022569* | Novel gene |  | **√** |
| 89 | *ENSSSCG00000022950* | Novel gene |  | **√** |
| 90 | *ENSSSCG00000023108* | Novel gene | **√** |  |
| 91 | *ENSSSCG00000023154* | Novel gene |  | **√** |
| 92 | *ENSSSCG00000023635* | Novel gene |  | **√** |
| 93 | *ENSSSCG00000023833* | Novel gene |  | **√** |
| 94 | *ENSSSCG00000023896* | Novel gene |  | **√** |
| 95 | *ENSSSCG00000025072* | Novel gene | **√** |  |
| 96 | *ENSSSCG00000025186* | Novel gene |  | **√** |
| 97 | *ENSSSCG00000025882* | Novel gene |  | **√** |
| 98 | *ENSSSCG00000025899* | Novel gene | **√** |  |
| 99 | *ENSSSCG00000026310* | Novel gene | **√** |  |
| 100 | *ENSSSCG00000026403* | Novel gene |  | **√** |
| 101 | *ENSSSCG00000027137* | Novel gene |  | **√** |
| 102 | *ENSSSCG00000027138* | Novel gene | **√** |  |
| 103 | *ENSSSCG00000027556* | Novel gene |  | **√** |
| 104 | *ENSSSCG00000028256* | Novel gene |  | **√** |
| 105 | *ENSSSCG00000028633* | Novel gene |  | **√** |
| 106 | *ENSSSCG00000028677* | Novel gene |  | **√** |
| 107 | *ENSSSCG00000029134* | Novel gene |  | **√** |
| 108 | *ENSSSCG00000029291* | Novel gene |  | **√** |
| 109 | *ENSSSCG00000029344* | Novel gene | **√** |  |
| 110 | *ENSSSCG00000029381* | Novel gene |  | **√** |
| 111 | *ENSSSCG00000029648* | Novel gene | **√** |  |
| 112 | *ENSSSCG00000029680* | Novel gene |  | **√** |
| 113 | *ENSSSCG00000029762* | Novel gene |  | **√** |
| 114 | *ENSSSCG00000029924* | Novel gene | **√** |  |
| 115 | *ENSSSCG00000030382* | Novel gene | **√** |  |
| 116 | *ENSSSCG00000030389* | Novel gene |  | **√** |
| 117 | *EPAS1* | Endothelial PAS domain protein 1 | **√** |  |
| 118 | *EPS15* | Epidermal growth factor receptor pathway substrate 15 |  | **√** |
| 119 | *EWSR1* | EWS RNA-binding protein 1 |  | **√** |
| 120 | *EXD1* | Exonuclease 3'-5' domain containing 1 |  | **√** |
| 121 | *EXOSC10* | Exosome component 10 | **√** |  |
| 122 | *EZH2* | Enhancer of zeste 2 polycomb repressive complex 2 subunit | **√** |  |
| 123 | *F2* | Coagulation factor II (thrombin) |  | **√** |
| 124 | *FAM120A* | Family with sequence similarity 120A | **√** |  |
| 125 | *FAM133B* | Family with sequence similarity 133, member B |  | **√** |
| 126 | *FAM149A* | Family with sequence similarity 149, member A |  | **√** |
| 127 | *FAM98C* | Family with sequence similarity 98, member C |  | **√** |
| 128 | *FBXO21* | F-box protein 21 |  | **√** |
| 129 | *FMNL2* | Formin-like 2 |  | **√** |
| 130 | *FNDC3B* | Fibronectin type III domain containing 3B |  | **√** |
| 131 | *FRAP1* | Mechanistic target of rapamycin (Serine/Threonine Kinase) | **√** |  |
| 132 | *FUCA2* | Fucosidase, alpha-L- 2, plasma |  | **√** |
| 133 | *G2E3* | G2/M-phase specific E3 ubiquitin protein ligase |  | **√** |
| 134 | *GABRR2* | Gamma-aminobutyric acid (GABA) A receptor, rho 2 |  | **√** |
| 135 | *GALNT3* | Polypeptide N-acetylgalactosaminyltransferase 3 | **√** |  |
| 136 | *GCNT3* | Glucosaminyl (N-acetyl) transferase 3, mucin type |  | **√** |
| 137 | *GGN* | Gametogenetin |  | **√** |
| 138 | *GRK7* | G protein-coupled receptor kinase 7 |  | **√** |
| 139 | *GRM3* | Glutamate receptor, metabotropic 3 |  | **√** |
| 140 | *GTF2H3* | General transcription factor IIH, polypeptide 3, 34kda |  | **√** |
| 141 | *HIF1A* | Hypoxia inducible factor 1, alpha subunit (basic helix-loop-helix transcription factor) |  | **√** |
| 142 | *HINT3* | Histidine triad nucleotide binding protein 3 |  | **√** |
| 143 | *HOXC10* | Homeobox C10 | **√** |  |
| 144 | *HS2ST1* | Heparan sulfate 2-O-sulfotransferase 1 |  | **√** |
| 145 | *HTT* | Huntingtin |  | **√** |
| 146 | *IFI30* | Interferon, gamma-inducible protein 30 | **√** |  |
| 147 | *IFT57* | Intraflagellar transport 57 | **√** |  |
| 148 | *INO80* | INO80 complex subunit |  | **√** |
| 149 | *KHNYN* | KH and NYN domain containing |  | **√** |
| 150 | *KIAA0895* | KIAA0895 | **√** |  |
| 151 | *KIAA1324L* | KIAA1324-like |  | **√** |
| 152 | *KIF1B* | Kinesin family member 1B |  | **√** |
| 153 | *KRIT1* | KRIT1, ankyrin repeat containing |  | **√** |
| 154 | *LEF1* | Lymphoid enhancer-binding factor 1 |  | **√** |
| 155 | *LRRC23* | Leucine rich repeat containing 23 |  | **√** |
| 156 | *LRRD1* | Leucine-rich repeats and death domain containing 1 |  | **√** |
| 157 | *MARCH7* | Membrane-associated ring finger (C3HC4) 7, E3 ubiquitin protein ligase |  | **√** |
| 158 | *MBD3* | Methyl-cpg binding domain protein 3 | **√** |  |
| 159 | *METAP1* | Methionyl aminopeptidase 1 |  | **√** |
| 160 | *METT5D1* | Methyltransferase Like 15 |  | **√** |
| 161 | *METTL5* | Methyltransferase like 5 | **√** |  |
| 162 | *MEX3D* | Mex-3 RNA binding family member D | **√** |  |
| 163 | *MFI2* | Antigen p97 (melanoma associated) identified by monoclonal antibodies 133.2 and 96.5 |  | **√** |
| 164 | *MFSD2A* | Major facilitator superfamily domain containing 2A |  | **√** |
| 165 | *MIP* | Major intrinsic protein of lens fiber |  | **√** |
| 166 | *MLNR* | Motilin receptor |  | **√** |
| 167 | *MORC1* | MORC family CW-type zinc finger 1 |  | **√** |
| 168 | *MRPS5* | Mitochondrial ribosomal protein S5 |  | **√** |
| 169 | *MSH4* | Muts homolog 4 | **√** |  |
| 170 | *MTMR3* | Myotubularin related protein 3 |  | **√** |
| 171 | *MUC13* | Mucin 13, cell surface associated | **√** |  |
| 172 | *MUC19* | Mucin 19, oligomeric |  | **√** |
| 173 | *MYCBP* | MYC binding protein |  | **√** |
| 174 | *NAGA* | N-acetylgalactosaminidase, alpha- | **√** |  |
| 175 | *NAV2* | Neuron navigator 2 |  | **√** |
| 176 | *NCOA7* | Nuclear receptor coactivator 7 |  | **√** |
| 177 | *NDUFA6* | NADH dehydrogenase (ubiquinone) 1 alpha subcomplex, 6, 14kda | **√** |  |
| 178 | *NF2* | Neurofibromin 2 (merlin) |  | **√** |
| 179 | *NIPSNAP1* | Nipsnap homolog 1 (C. Elegans) |  | **√** |
| 180 | *NOX4* | NADPH oxidase 4 |  | **√** |
| 181 | *NPPB* | Natriuretic peptide B |  | **√** |
| 182 | *NRG3* | Neuregulin 3 | **√** |  |
| 183 | *NSUN6* | NOP2/Sun domain family, member 6 |  | **√** |
| 184 | *NTRK3* | Neurotrophic tyrosine kinase, receptor, type 3 |  | **√** |
| 185 | *OSBPL1A* | Oxysterol binding protein-like 1A |  | **√** |
| 186 | *OSBPL9* | Oxysterol binding protein-like 9 |  | **√** |
| 187 | *PDE1A* | Phosphodiesterase 1A, calmodulin-dependent | **√** |  |
| 188 | *PDE3B* | Phosphodiesterase 3B, cgmp-inhibited |  | **√** |
| 189 | *PEX1* | Peroxisomal biogenesis factor 1 |  | **√** |
| 190 | *PHF14* | PHD finger protein 14 |  | **√** |
| 191 | *PHF19* | PHD finger protein 19 | **√** |  |
| 192 | *PHF2* | PHD finger protein 2 | **√** |  |
| 193 | *PIK3R2* | Phosphoinositide-3-kinase, regulatory subunit 2 (beta) | **√** |  |
| 194 | *PLA2R1* | Phospholipase A2 receptor 1, 180kda | **√** | **√** |
| 195 | *POLR2D* | Polymerase (RNA) II (DNA directed) polypeptide D |  | **√** |
| 196 | *PPP1R2* | Protein phosphatase 1, regulatory (inhibitor) subunit 2 |  | **√** |
| 197 | *PRMT1* | Protein arginine methyltransferase 1 | **√** |  |
| 198 | *PSMA1* | Proteasome (prosome, macropain) subunit, alpha type, 1 |  | **√** |
| 199 | *PSMA6* | Proteasome (prosome, macropain) subunit, alpha type, 6 |  | **√** |
| 200 | *PSMD8* | Proteasome (prosome, macropain) 26S subunit, non-atpase, 8 |  | **√** |
| 201 | *PTPN23* | Protein tyrosine phosphatase, non-receptor type 23 |  | **√** |
| 202 | *RABGGTA* | Rab geranylgeranyltransferase, alpha subunit |  | **√** |
| 203 | *RAPGEF2* | Rap guanine nucleotide exchange factor (GEF) 2 |  | **√** |
| 204 | *RBFOX1* | RNA binding protein, fox-1 homolog (C. Elegans) 1 |  | **√** |
| 205 | *RBMS2* | RNA binding motif, single stranded interacting protein 2 |  | **√** |
| 206 | *RDX* | Radixin |  | **√** |
| 207 | *RFX4* | Regulatory factor X, 4 (influences HLA class II expression) | **√** | **√** |
| 208 | *RHBDD3* | Rhomboid domain containing 3 |  | **√** |
| 209 | *RIN3* | Ras and Rab interactor 3 |  | **√** |
| 210 | *RNF150* | Ring finger protein 150 |  | **√** |
| 211 | *RNF185* | Ring finger protein 185 |  | **√** |
| 212 | *RNF4* | Ring finger protein 4 |  | **√** |
| 213 | *RNF7* | Ring finger protein 7 |  | **√** |
| 214 | *RRAGC* | Ras-related GTP binding C |  | **√** |
| 215 | *RRAS* | Related RAS viral (r-ras) oncogene homolog | **√** |  |
| 216 | *SAP130* | Sin3A-associated protein, 130kda |  | **√** |
| 217 | *SCAF1* | SR-related CTD-associated factor 1 | **√** |  |
| 218 | *SCAP* | SREBF chaperone |  | **√** |
| 219 | *SCUBE2* | Signal peptide, CUB domain, EGF-like 2 |  | **√** |
| 220 | *SDR39U1* | Short chain dehydrogenase/reductase family 39U, member 1 |  | **√** |
| 221 | *SEC63* | SEC63 homolog (S. Cerevisiae) | **√** |  |
| 222 | *SENP7* | SUMO1/sentrin specific peptidase 7 |  | **√** |
| 223 | *SEPW1* | Selenoprotein W, 1 | **√** |  |
| 224 | *SERGEF* | Secretion regulating guanine nucleotide exchange factor |  | **√** |
| 225 | *SGSM1* | Small G protein signaling modulator 1 | **√** |  |
| 226 | *SGTA* | Small glutamine-rich tetratricopeptide repeat (TPR)-containing, alpha | **√** |  |
| 227 | *SH3GL2* | SH3-domain GRB2-like 2 |  | **√** |
| 228 | *SLC7A13* | Solute carrier family 7 (anionic amino acid transporter), member 13 | **√** |  |
| 229 | *SMN1* | Survival of motor neuron 1, telomeric |  | **√** |
| 230 | *SNORA11* | Small nucleolar RNA, H/ACA box 11 |  | **√** |
| 231 | *SNORA31* | Small nucleolar RNA, H/ACA box 31 |  | **√** |
| 232 | *SNTB1* | Syntrophin, beta 1 (dystrophin-associated protein A1, 59kda, basic component 1) |  | **√** |
| 233 | *SORCS3* | Sortilin-related VPS10 domain containing receptor 3 |  | **√** |
| 234 | *SPECC1L* | Sperm antigen with calponin homology and coiled-coil domains 1-like |  | **√** |
| 235 | *SPINT2* | Serine peptidase inhibitor, Kunitz type, 2 |  | **√** |
| 236 | *SPRED3* | Sprouty-related, EVH1 domain containing 3 |  | **√** |
| 237 | *SPSB2* | Spla/ryanodine receptor domain and SOCS box containing 2 |  | **√** |
| 238 | *SSB* | Sjogren syndrome antigen B (autoantigen La) | **√** |  |
| 239 | *TANC1* | Tetratricopeptide repeat, ankyrin repeat and coiled-coil containing 1 |  | **√** |
| 240 | *TCF12* | Transcription factor 12 |  | **√** |
| 241 | *TCTN2* | Tectonic family member 2 |  | **√** |
| 242 | *TEX2* | Testis expressed 2 |  | **√** |
| 243 | *TGM1* | Transglutaminase 1 |  | **√** |
| 244 | *THADA* | Thyroid adenoma associated |  | **√** |
| 245 | *THOP1* | Thimet oligopeptidase 1 | **√** |  |
| 246 | *THSD7A* | Thrombospondin, type I, domain containing 7A | **√** |  |
| 247 | *TIMELESS* | Timeless circadian clock |  | **√** |
| 248 | *TM9SF4* | Transmembrane 9 superfamily protein member 4 |  | **√** |
| 249 | *TMEM106C* | Transmembrane protein 106C | **√** |  |
| 250 | *TMEM132C* | Transmembrane protein 132C |  | **√** |
| 251 | *TMEM211* | Transmembrane protein 211 | **√** |  |
| 252 | *TMPRSS2* | Transmembrane protease, serine 2 |  | **√** |
| 253 | *TNFSF10* | Tumor necrosis factor (ligand) superfamily, member 10 |  | **√** |
| 254 | *TRAT1* | T cell receptor associated transmembrane adaptor 1 |  | **√** |
| 255 | *TRIM2* | Tripartite motif containing 2 | **√** |  |
| 256 | *TRMT11* | Trna methyltransferase 11 homolog (S. Cerevisiae) |  | **√** |
| 257 | *TTBK2* | Tau tubulin kinase 2 |  | **√** |
| 258 | *TXN* | Thioredoxin |  | **√** |
| 259 | *TXNDC8* | Thioredoxin domain containing 8 (spermatozoa) |  | **√** |
| 260 | *U6* | U6 spliceosomal RNA |  | **√** |
| 261 | *UBIAD1* | Ubia prenyltransferase domain containing 1 |  | **√** |
| 262 | *UIMC1* | Ubiquitin interaction motif containing 1 | **√** |  |
| 263 | *UNC50* | Unc-50 homolog (C. Elegans) | **√** |  |
| 264 | *UQCR11* | Ubiquinol-cytochrome c reductase, complex III subunit XI | **√** |  |
| 265 | *UST* | Uronyl-2-sulfotransferase |  | **√** |
| 266 | *WDR33* | WD repeat domain 33 |  | **√** |
| 267 | *WRN* | Werner syndrome, recq helicase-like |  | **√** |
| 268 | *ZMIZ1* | Zinc finger, MIZ-type containing 1 |  | **√** |
| 269 | *ZNF330* | Zinc finger protein 330 |  | **√** |
| 270 | *ZNF408* | Zinc finger protein 408 |  | **√** |
| 271 | *ZNF420* | Zinc finger protein 420 |  | **√** |
| 272 | *ZNF527* | Zinc finger protein 527 |  | **√** |
| 273 | *ZNF703* | Zinc finger protein 703 | **√** |  |

**Supplementary Table s7:** Classification of physiological functions of some of the PSGs.

| **Physiological Functions** | **Gene No.** | **PSGs** |
| --- | --- | --- |
| Tumor or cancer | 39 | *RRAS,ZNF703,SGSM1,PHF19,EWSR1,MYCBP,PLA2R1,CBLB,BUB1B,SPINT2, SGTA,THOP1,PHF2,HOXC10,PTPN23,TRMT11,LEF1,ZMIZ1,SPRED3,MFSD2A, C6ORF97,THADA,COL16A1,FBXO21,ANLN,TMPRSS2,SCUBE2,RDX,SEPW1, C3ORF75,NCOA7,TM9SF4,PSMD8,FNDC3B,SH3GL2,GCNT3,OSBPL1A,TANC1, ABCB4* |
| Cardiovascular System | 34 | *THSD7A,PIK3R2,TRIM2,BCR,F2,NOX4,KRIT1,TXN,RAPGEF2,PSMA6,APOD, EIF4E,BCL2L12,CYP4F2,UNC50,ARHGEF26,DLG1,NPPB,UBIAD1,CUL5,ADD1, CLCN6,FMNL2,EZH2,METAP1,MFI2,CNDP1,CNDP2,ZNF408,MTMR3,NF2, NTRK3,RABGGTA,NAGA* |
| UV damage or DNA repair | 12 | *UIMC1,EXOSC10,FRAP1,RNF7,WRN,SENP7,ZNF420,SAP130,INO80,PRMT1, MSH4,GTF2H3* |
| Spermatogenesis | 12 | *CLGN,RFX4,MORC1,TXNDC8,GGN,CATSPERG,DPPA4,DHX36,PPP1R2, GALNT3,NRG3,DKK3* |
| Neural development | 11 | *CCDC91,SORCS3,C18orf1,ENO2,GRM3,NAV2,BARHL2,HTT,RBFOX1,TTBK2, NIPSNAP1* |
| Response to hypoxia | 6 | *EPAS1,HIF1A,RNF4,TNFSF10,PDE1A,PDE3B* |
| Mitochondria or respiratory chain | 6 | *UQCR11,NDUFA6,COA5,COQ3,ELMOD2,KIF1B* |
| Immunity | 6 | *IFI30,MUC13,TRAT1,SLC7A13,RHBDD3,SNTB1* |
| Lung development and respiration | 5 | *CYSLTR2,PHF14,RNF150,TIMELESS,SCAP* |
| Apoptosis | 3 | *CSRNP3,CIDEB,FAM120A* |
| Embryo development | 2 | *G2E3,DDX55* |

**Supplementary Table s8:** Related analysis between enrichment ratio and *F*_ST._

| **Annoted types** | **Whole genome** | | | **Selective sweeps** | | |
| --- | --- | --- | --- | --- | --- | --- |
|  | *P* value | Coefficient | △F | *P* value | Coefficient | △F |
| Coding | 0.150 | -0.529 | -0.610 | 0.264 | 3.278 | 1.616 |
| UTR | 0.032* | -0.597 | -0.318 | 0.777 | 0.605 | 0.661 |
| Intron | 0.525 | -0.044 | -0.006 | 0.036* | -0.563 | -0.232 |
| Intergenic | 0.358 | 0.024 | 0.009 | 0.015* | 0.240 | 0.094 |
| Conserved | 2.36E-06** | -1.026 | -0.730 | 0.827 | 0.149 | 0.197 |
| Histone | 3.58E-06** | -0.749 | -0.458 | 0.063 | 0.337 | 0.168 |
| FAIRE | 2.09E-07** | -0.799 | -0.508 | 0.060 | 1.235 | 0.488 |
| DHS | 6.06E-05** | -0.751 | -0.476 | 0.903 | 0.017 | 0.043 |
| TFBS | 6.28E-05** | -0.677 | -0.453 | 0.914 | 0.032 | 0.032 |
| Motif | 0.386 | -0.287 | -0.046 | 0.033* | 2.444 | 1.163 |

Note: △F= Enrichment ratio _maximal_ *_F_*_st_ - Enrichment ratio _minimal_ *_F_*_st_

**Supplementary Table s9:** Predicted Motifs in selected sweep regions of Tibetan pigs.

| ID | Motif Position in Pig | Differentiated SNP | Derived  Allele | Ancestral  Allele | *F*_ST_ | Derived frequency  in Tibetan pigs | Derived frequency  in Control pigs | TF of Motif |
| --- | --- | --- | --- | --- | --- | --- | --- | --- |
| M-1 | Chr1.293009235.293009241 | Chr1:293009236 | C | G | 0.22 | 0.36 | 0 | UAK49 |
| M-2 | Chr1.293013513.293013527 | Chr1:293013518 | A | G | 0.31 | 0.55 | 0.04 | YY1 |
| M-3 | Chr1.293013572.293013590 | Chr1:293013585 | T | C | 0.71 | 0.92 | 0.08 | UAK42 |
| M-4 | Chr2.61358862.61358878 | Chr2:61358867 | G | A | 0.41 | 0.73 | 0.1 | FOXA |
| M-5 | Chr2.61359136.61359150 | Chr2:61359137 | G | A | 0.53 | 0.86 | 0.13 | SP1 |
| **M-6** | **Chr2.****61359252.61359269** | **Chr2:****61359264** | **G** | **A** | **0.40** | **0.81** | **0.18** | **NFE2** |
| M-7 | Chr2.61368472.61368486 | Chr2:61368486 | G | A | 0.32 | 0.78 | 0.21 | ZNF143 |
| M-8 | Chr2.61368473.61368487 | Chr2:61368486 | G | A | 0.32 | 0.78 | 0.21 | CTCF-ext |
| M-9 | Chr2.61368473.61368502 | Chr2:61368486 | G | A | 0.32 | 0.78 | 0.21 | CTCF |
| M-10 | Chr2.61368476.61368503 | Chr2:61368486 | G | A | 0.32 | 0.78 | 0.21 | CREB, CREB-ext |
| M-11 | Chr2.76389700.76389759 | Chr2:76389756 | C | A | 0.23 | 0.41 | 0.02 | SP1 |
| M-12 | Chr2.76391009.76391040 | Chr2:76391012 | G | C | 0.24 | 0.39 | 0 | TCF12 |
| M-13 | Chr2.76391012.76391019 | Chr2:76391012 | G | C | 0.24 | 0.39 | 0 | V_AP4_01 |
| M-14 | Chr2.76391012.76391022 | Chr2:76391012 | G | C | 0.24 | 0.39 | 0 | TCF3 |
| M-15 | Chr3.58229718.58229750 | Chr3:58229731 | A | G | 0.28 | 0.82 | 0.29 | UAK25 |
| **M-16** | **Chr3.100231640.100231653** | **Chr3:100231648 Chr3:100231649** | **T  G** | **C A** | **0.27 0.35** | **0.42 0.71** | **0 0.13** | **HNF4** |
| M-17 | Chr3.100231982.100232000 | Chr3:100231991 Chr3:100232000 | A G | G A | 0.38 0.31 | 0.87 0.85 | 0.26 0.3 | v-Maf |
| **M-18** | **Chr3.100232087.100232104** | **Chr3:100232103** | **A** | **G** | **0.38** | **0.62** | **0.04** | **PRDM1** |
| M-19 | Chr5.13459041.13459079 | Chr5:13459076 | A | G | 0.26 | 0.69 | 0.18 | PU1 |
| M-20 | Chr6.50448899.50448917 | Chr6:50448906 | A | G | 0.16 | 1 | 0.73 | UAK42 |
| M-21 | Chr7.44057016.44057030 | Chr7:44057020 | G | T | 0.32 | 0.85 | 0.29 | ZNF263 |
| M-22 | Chr7.44075290.44075312 | Chr7:44075312 | T | C | 0.23 | 0.7 | 0.22 | UAK42 |
| M-23 | Chr8.91797430.91797468 | Chr8:91797437 | C | T | 0.36 | 0.63 | 0.06 | PU1 |
| M-24 | Chr8.91926762.91926772 | Chr8:91926763 | A | G | 0.18 | 0.31 | 0 | UA9 |
| M-25 | Chr8.91947502.91947518 | Chr8:91947511 | A | C | 0.24 | 0.92 | 0.47 | FOXA |
| M-26 | Chr8.116819514.116819532 | Chr8:116819528 | G | A | 0.22 | 0.36 | 0 | NFE2 |
| M-27 | Chr8.116819516.116819532 | Chr8:116819528 | G | A | 0.22 | 0.36 | 0 | v-Maf |
| M-28 | Chr8.116819516.116819541 | Chr8:116819528 | G | A | 0.22 | 0.36 | 0 | AP1 |
| M-29 | Chr9.89468330.89468340 | Chr9:89468331 | G | A | 0.25 | 0.92 | 0.46 | UAK38 |
| M-30 | Chr9.95585500.95585522 | Chr9:95585511 | T | C | 0.25 | 0.8 | 0.3 | NFY, NFY-UA2, UA2 |
| M-31 | Chr9.95587823.95587837 | Chr9:95587834 | A | C | 0.19 | 0.77 | 0.34 | RFX5 |
| M-32 | Chr9.95588753.95588767 | Chr9:95588767 | G | A | 0.23 | 0.8 | 0.32 | UAK55 |
| M-33 | Chr14.45274372.45274384 | Chr14:45274374 | T | A | 0.19 | 0.63 | 0.2 | ZNF263 |
| M-34 | Chr14.45277960.45277974 | Chr14:45277969 | G | T | 0.19 | 0.6 | 0.18 | CTCF |
| M-35 | Chr14.45780524.45780542 | Chr14:45780526 | T | C | 0.21 | 0.43 | 0.04 | AP2 |
| M-36 | Chr14.45780859.45780873 | Chr14:45780869 | A | G | 0.24 | 0.48 | 0.05 | UAK56 |
| M-37 | Chr14.45780863.45780877 | Chr14:45780869 | A | G | 0.24 | 0.48 | 0.05 | UAK54 |
| M-38 | Chr14.45800186.45800207 | Chr14:45800187 | A | G | 0.27 | 0.5 | 0.04 | v-Maf |
| M-39 | Chr14.45803558.45803571 | Chr14:45803561 | C | T | 0.27 | 0.5 | 0.04 | SP1 |
| M-40 | Chr14.45803559.45803569 | Chr14:45803561 | C | T | 0.27 | 0.5 | 0.04 | ZNF281 |
| M-41 | Chr15.55817984.55817991 | Chr15:55817990 | C | A | 0.17 | 0.84 | 0.45 | SP1 |
| M-42 | Chr15.55826627.55826641 | Chr15:55826638 | T | A | 0.28 | 0.62 | 0.11 | CTCF |
| M-43 | Chr15.73549724.73549736 | Chr15:73549735 | G | A | 0.32 | 0.6 | 0.07 | HNF4 |
| M-44 | Chr15.97306723.97306733 | Chr15:97306730 | C | T | 0.19 | 0.52 | 0.11 | USF |

Note: M-6, M-16 and M-18 were chose for luciferase assay.
